# Is reproductive strategy a key factor in understanding the evolutionary history of Southern Ocean Asteroidea (Echinodermata)?

**DOI:** 10.1002/ece3.5280

**Published:** 2019-07-16

**Authors:** Camille Moreau, Bruno Danis, Quentin Jossart, Marc Eléaume, Chester Sands, Guillaume Achaz, Antonio Agüera, Thomas Saucède

**Affiliations:** ^1^ Marine Biology Lab Université Libre de Bruxelles (ULB) Belgium; ^2^ Biogéosciences, UMR 6282 CNRS Université Bourgogne Franche‐Comté Dijon France; ^3^ Marine Biology Vrije Universiteit Brussel (VUB) Brussels Belgium; ^4^ Institut de Systématique, Evolution, Biodiversité (ISYEB), Muséum national d'Histoire naturelle, CNRS Sorbonne Université Paris France; ^5^ ^5^Natural Environment Research Council British Antarctic Survey Cambridge UK; ^6^ Centre Interdisciplinaire de Recherche en Biologie (CIRB), CNRS INSERM, Collège de France Paris France

**Keywords:** Antarctica, Asteroidea, bipolarity, brooding, Echinodermata, emergence, invertebrate, thermohaline expressway, trans‐Antarctic seaway

## Abstract

Life traits such as reproductive strategy can be determining factors of species evolutionary history and explain the resulting diversity patterns. This can be investigated using phylogeographic analyses of genetic units. In this work, the genetic structure of five asteroid genera with contrasting reproductive strategies (brooding: *Diplasterias*, *Notasterias* and *Lysasterias* versus broadcasting: *Psilaster* and *Bathybiaster*) was investigated in the Southern Ocean. Over 1,400 mtDNA cytochrome C oxidase subunit I (COI) sequences were analysed using five species delineation methods (ABGD, ASAP, mPTP, sGMYC and mGMYC), two phylogenetic reconstructions (ML and BA), and molecular clock calibrations, in order to examine the weight of reproductive strategy in the observed differences among phylogeographic patterns. We hypothesised that brooding species would show higher levels of genetic diversity and species richness along with a clearer geographic structuring than broadcasting species. In contrast, genetic diversity and species richness were not found to be significantly different between brooders and broadcasters, but broadcasters are less spatially structured than brooders supporting our initial hypothesis and suggesting more complex evolutionary histories associated to this reproductive strategy. Broadcasters' phylogeography can be explained by different scenarios including deep‐sea colonisation routes, bipolarity or cosmopolitanism, and sub‐Antarctic emergence for the genus *Bathybiaster*; Antarctic‐ New Zealand faunal exchanges across the Polar Front for the genus *Psilaster*. Brooders' phylogeography could support the previously formulated hypothesis of a past trans‐Antarctic seaway established between the Ross and the Weddell seas during the Plio‐Pleistocene. Our results also show, for the first time, that the Weddell Sea is populated by a mixed asteroid fauna originating from both the East and West Antarctic.

## 1 **INTRODUCTION**

The diversity of marine life in the Southern Ocean (SO) has long been underestimated, and many taxa could be overlooked or misidentified due to the reliance on systematics based solely on morphological characters (Clarke, 2008; Gutt, Sirenko, Smirnov, & Arntz, 2004). The growing awareness of these issues and the lack of representative sampling (Griffiths, Putte, & Danis, 2014) have prompted Antarctic marine biologists to undertake a series of comprehensive census surveys covering a wide range of taxa in the last two decades. International efforts such as the International Polar Year (IPY 2007–2008) and the Census of Antarctic Marine Life (CAML 2005–2010) have been the launching pads for a better assessment of SO biodiversity and its underlying ecological processes. Recent studies that have applied molecular techniques to these SO specimens, exploring diversity, systematics, and phylogeography, have significantly increased our understanding of Antarctic benthic ecosystems (Sands, O'Hara, Barnes, & Martín‐Ledo, 2015). These international SO sampling expeditions have achieved several major objectives, such as the creation of a baseline census of biodiversity (De Broyer & Danis, 2011; Griffiths, Danis, & Clarke, 2011), proposing how evolution has been influenced by the regional geological, climatic, and oceanographic histories (Fraser, Nikula, Spencer, & Waters, 2009; González‐Wevar et al., 2018), and disentangling phylogeographic patterns at lower taxonomic levels to better understand relationships among populations and species on a case‐by‐case strategy (Brasier et al., 2017; Dömel, Melzer, Harder, Mahon, & Leese, 2017). This extensive work led to the discovery and the description of many new species (d'Udekem d'Acoz & Verheye, 2017; Janosik & Halanych, 2010). More importantly, these studies show the frequent discordance existing between traditional (morphology‐based) and molecular (DNA‐based) methods for assessing species diversity (Dömel et al., 2017; Janosik, Mahon, & Halanych, 2011). Cryptic speciation is a documented source of species diversity underestimation, but it is not the only one. A recent study on the most studied sea star species in the SO, *Odontaster validus*, Peck, Clark, and Dunn (2018) showed that polymorphism in some morphological characters could lead to misidentification of this frequently encountered species. Incorrect taxonomic assignments due to the lack of clear identification keys (Allcock & Griffiths, 2014), descriptions of nominal species based on distribution only (Díaz, Féral, David, Saucède, & Poulin, 2011; Saucede et al., 2015), and descriptions based on juvenile specimens (Roberts, Hopcroft, & Hosie, 2014) or on deteriorated specimens due to inappropriate conservation practices are all common limitations in meaningful biodiversity assessments (Meyer, 2016).

Processes that have and continue to drive complex diversity patterns in the SO are far from being fully understood, but the role of certain drivers has been demonstrated in a number of molecular studies. Processes can be extrinsic (e.g., paleogeographic, climatic, oceanographic) or intrinsic (life history traits) (Allcock & Strugnell, 2012; Thatje, 2012), and their combined effects are cumulative, making identification of explanatory processes a difficult endeavor. Life history traits such as reproductive strategies have been proven to shape the genetic structure of species in contrasting ways (Modica, Russini, Fassio, & Oliverio, 2017). Most marine benthic organisms show low to zero mobility during the adult stage. The dispersal capacity of larvae is thus expected to condition population genetic structure (Bowen, Bass, Muss, Carlin, & Robertson, 2006; Purcell, Cowen, Hughes, & Williams, 2006). Larval development (e.g., planktotrophic, lecithotrophic), parental care (brooders vs. broadcasters), and pelagic larval duration (from days to several months; Selkoe & Toonen, 2011) are intrinsic factors that can combine with extrinsic factors, such as oceanographic currents or geological history, to determine the population genetic structure (Hoffman, Clarke, Linse, & Peck, 2011; Jossart et al., 2017).

As a consequence of their high diversity and differentiated functional roles, benthic invertebrates have been the subject of many studies on genetic diversity and connectivity (see Riesgo, Taboada, & Avila, 2015 for a review). Sea stars (Asteroidea, Echinodermata) represent a diversified, abundant, and common ecological group in SO benthic habitats (Jossart, Moreau, Agüera, De Broyer, & Danis, 2015). To date, around 300 species have been recorded from the SO (Moreau, Agüera, Jossart, & Danis, 2015; Moreau et al., 2018) but it is likely that many more remain to be described. Recent studies based on species morphological identification tried to disentangle distribution patterns and biogeographic relationships in sea stars at the scale of the entire SO (Moles et al., 2015; Moreau et al., 2017). These studies described the spatial structure of asteroid assemblages and stressed the major influence of life history traits, among which reproductive strategy appeared to have the most notable effect (Moreau et al., 2017).

Few molecular studies have been performed on SO asteroids, and they are nearly exclusively focused on the common *Odontaster* genus (Janosik & Halanych, 2010; Janosik et al., 2011). The phylogeny and evolutionary history of the Asteroidea in the SO have, however, been discussed in several studies (Mah & Foltz, 2011a, 2011b, 2014; Mah et al., 2015). In their comprehensive studies on the molecular phylogeny of the superorders Valvatacea and Forcipulatacea, Mah and Foltz (2011a) describe some diversification processes in several Antarctic and sub‐Antarctic families (e.g., Odontasteridae, Antarctic Asteriidae) but they did not analyze the lower taxonomic levels (i.e., genera and species).

Brooders and broadcasters have contrasting dispersal capabilities (low‐range dispersal in brooders vs. high range in broadcasters), and brooding taxa are usually hypothesized to display (a) higher genetic diversity, (b) greater species richness, and (c) more genetic differentiation through space than broadcasters (Modica et al., 2017; Purcell et al., 2006). In the present study, we tested these assumptions by investigating the genetic diversity and the phylogeography of five widely distributed asteroid genera across the SO. We selected genera with contrasting reproductive strategies in two distinct clades: the three brooding genera *Diplasterias*, *Lysasterias*, and *Notasterias* in the monophyletic family Asteriidae (Foltz, Bolton, Kelley, Kelley, & Nguyen, 2007; Mah & Foltz, 2011b), and the two broadcasting genera *Psilaster* and *Bathybiaster* in a monophyletic group of the family Astropectinidae (Bosch & Pearse, 1990; Mah & Foltz, 2011a).

## 2 MATERIALS AND METHODS

### 2.1 Comprehensive sampling and DNA sequence compilation

**Figure 1:**
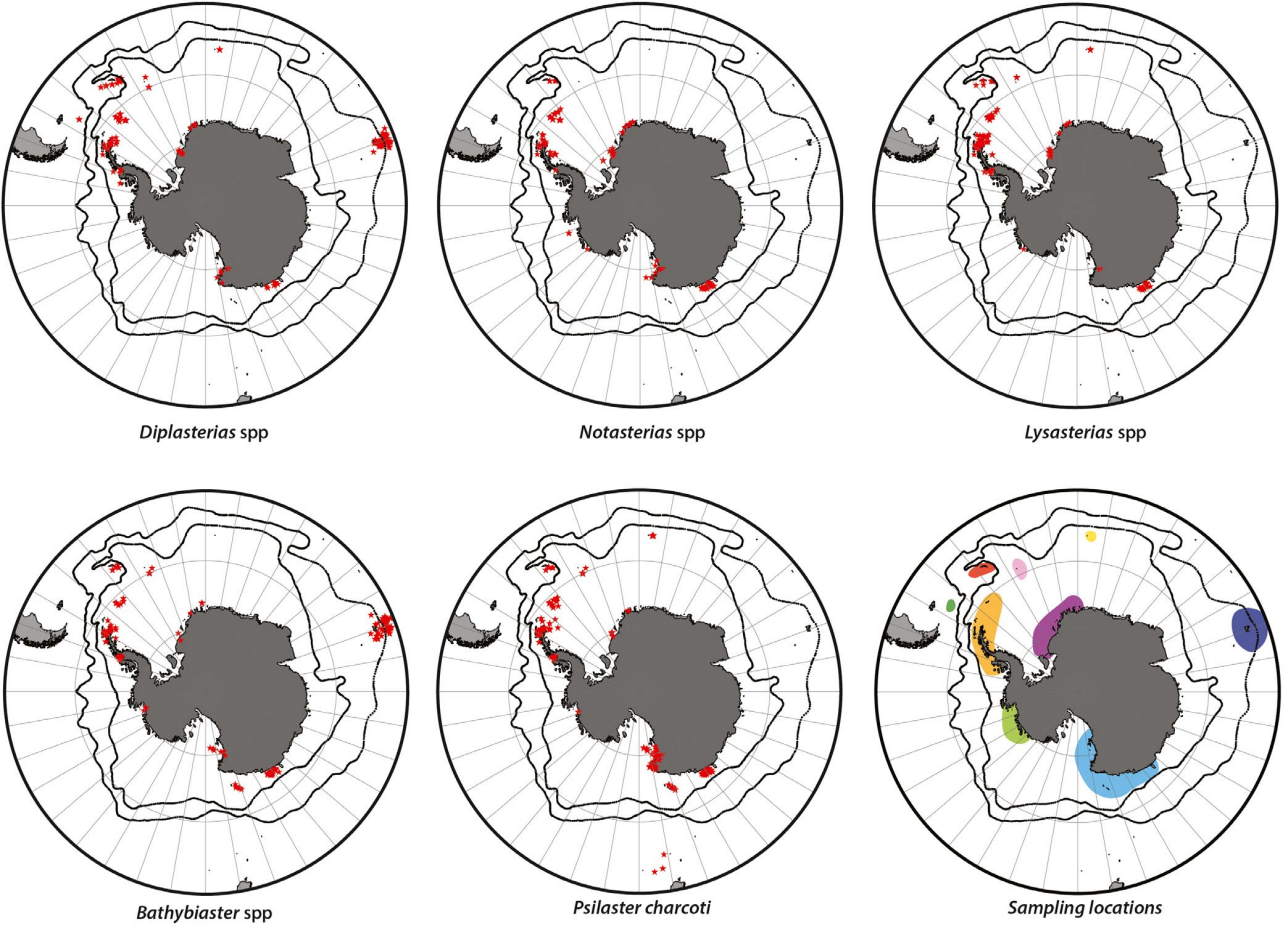
Maps of the SO indicating sample location for each target group (red stars). Sampling locations are labeled: red—South Georgia; pink—South Sandwich Islands; yellow—Bouvet Island; dark blue—Kerguelen Islands; light blue—East Antarctica; light green—Amundsen Sea; orange—Antarctic Peninsula; dark green—Burdwood Bank; and purple—Weddell Sea. Projection: South Pole Stereographic

Studied specimens were sampled during several expeditions in the SO (Figure 1; Appendix S1); all were preserved in 96% ethanol or frozen and identified at species or genus level (either by Christopher Mah or Camille Moreau). Genomic DNA was extracted using either a salting‐out protocol (modified from Sunnucks & Hales, 1996, with larger volumes and incubation at 70°C for 10 min to inhibit protein activity after digestion) or Qiagen DNeasy extraction kits. A fragment (612 nucleotides) of the mitochondrial gene cytochrome c oxidase subunit I (COI) was then amplified using the specific forward primer LCOech1aF1 for the class Asteroidea and the universal HCO2198 reverse primer (Folmer, Black, Hoeh, Lutz, & Vrijenhoek, 1994). COI sequences amplified using the same primers were also obtained through The Barcode of Life Data System (BOLD, Ratnasingham & Hebert, 2007) in both public and private datasets, accounting for 460 of the 1,416 sequences analyzed in this study (Appendix S1). As the Astropectinidae genera are also recorded outside the SO (under different nominal species), all available sequences for these from outside the SO were included within this study (Appendix S1). Reverse and forward sequences were edited and assembled using CodonCode Aligner v6.0.2 and translated using the echinoderm mitochondrial genetic code to ensure the absence of a stop codon. Sequences were aligned using the MUSCLE alignment process (Edgar, 2004). Base compositional heterogeneity was examined using match‐paired tests for symmetry (Ababneh, Jermiin, Ma, & Robinson, 2006) in SeqVis v1.5 (Ho et al., 2006).

### 2.2 Phylogenetic reconstruction

Due to relatively high genetic distances, phylogenetic relationships were reconstructed independently within the Asteriidae and the Astropectinidae. *Coscinasterias muricata* and *Thrissacanthias penicillatus* were used as outgroups, respectively, following previous phylogenetic studies (Mah & Foltz, 2011a, 2011b). Maximum likelihood (ML) and Bayesian analyses (BA) were used to construct the trees using only unique haplotypes. ML reconstructions were generated using a codon partitioned model and the GTR + G substitution model in RAxML v 8.1.2 (Stamatakis, 2014) through the raxmlGUI interface (Silvestro & Michalak, 2012). To assess branch support, 10 runs were realized with 1,000 thorough bootstraps each. The PartitionFinder v2 software (Lanfear, Frandsen, Wright, Senfeld, & Calcott, 2016) was used for the BA reconstructions on the CIPRES Science Gateway (Miller, Pfeiffer, & Schwartz, 2010) to select for best‐fit partitioning schemes and models of evolution. An XML file was created with BEAUti v1.8.4 (Drummond, Suchard, Xie, & Rambaut, 2012) using a partition for each codon position as specified by PartitionFinder v2, a strict clock model with a lognormal prior for “clock.rate” such that the median reflected a universal divergence time of echinoderm COI of 3.1%–3.5% per Myr (McCartney, Keller, & Lessios, 2000), a Markov chain Monte Carlo run of 20 × 10^6^ generations sampling every 1,000 trees, and a Yule process speciation prior. The XML file was used through the software BEAST v1.8.4 on the CIPRES Science Gateway (Miller et al., 2010) to reconstruct time‐calibrated trees. Tracer v1.6 allowed us to ensure an appropriate effective sampling size (ESS > 200) as recommended by the software documentation. TreeAnnotator v1.8.4 calculated a consensus tree which was visualized using FigTree v1.4.3 (http://tree.bio.ed.ac.uk/software/figtree/).

### 2.3 Species delineation

Several single‐locus methods of species delineation were used to delineate and explore diversity among the studied genera. Two of these methods, the Generalized Mixed Yule Coalescent (GMYC—Pons et al., 2006; Fujisawa & Barraclough, 2013) and the multirate Poisson Tree Processes (mPTP—Kapli et al., 2017), are tree‐based methods requiring an ultrametric tree for the former and a maximum likelihood tree for the latter. Both single (sGMYC)‐ and multiple‐threshold (mGMYC) models were investigated using the R package SPLITS (Ezard, Fujisawa, & Barraclough, 2009), and the ultrametric tree was obtained using BEAST during the phylogenetic reconstruction. The online web service (available at http://mptp.h-its.org) was used for mPTP with the ML tree constructed using RAxML. We also performed a distance‐based analysis using the Automatic Barcode Gap Discovery (ABGD—Puillandre, Lambert, Brouillet, & Achaz, 2012) on the online server (http://wwwabi.snv.jussieu.fr/public/abgd/abgdweb.html) with default settings for the prior range (0.001, 0.1), K80‐corrected distances, and a value of 1.5 for the relative gap width (X). We also used the newly developed ASAP method (http://wwwabi.snv.jussieu.fr/public/asap/; N. Puillandre et al., in prep.) with default settings and K80‐corrected distances. For subsequent analyses, we have selected the method giving the smallest number of delineated entities as recommended to avoid false interpretation (Carstens, Pelletier, Reid, & Satler, 2013). All molecular diversity indexes were calculated using the DnaSP v6 software (Rozas et al., 2017) for each previously delineated species. Differences in haplotype and nucleotide diversity between brooders and broadcasters were tested using a Wilcoxon–Mann–Whitney test (R Core Team, 2018). Only calculations for sample sizes ≥25 individuals were discussed as suggested by Goodall‐Copestake, Tarling, and Murphy (2012). Intra‐ and interspecific genetic distances within each genus were calculated using MEGA v7.0.18 (Kumar, Stecher, & Tamura, 2016) and the Kimura 2‐parameter model. Haplotype networks were generated using a TCS network method (Clement, Snell, Walker, Posada, & Crandall, 2002) inferred in the software PopART (http://popart.otago.ac.nz). The genus *Psilaster* being recovered as polyphyletic, we focus on the SO nominal species *Psilaster charcoti*.

## 3 RESULTS

In total, 1,416 sequences of 612bp were included in the analyses (Appendix S1, Table 1, Figure 1), varying from 224 for *Lysasterias* to 318 for *Notasterias*. Neither stop codons nor significant evidence for base heterogeneity were observed.

**Table 1:** Molecular diversity statistics for each delineated species. Statistics for taxonomic groups with *n* < 25 are not represented. *n*: number of sequences. *π*: nucleotide diversity. *H*: haplotype diversity

Taxonomic group	*n*	*π*	*n* of haplotype	*H*	Segregating sites	Mean intraspecific distance
Brooders						
*Lysasterias* sp1	81	0.01000 ± 0.00062	16	0.831 ± 0.027	25	0.0103 ± 0.0069
*Lysasterias* sp2	78	0.01146 ± 0.00093	12	0.840 ± 0.019	26	0.0118 ± 0.0092
*Lysasterias* sp3	56	0.00825 ± 0.00095	13	0.765 ± 0.055	21	0.0085 ± 0.0073
*Notasterias* sp1	236	0.00561 ± 0.00032	23	0.733 ± 0.027	31	0.0057 ± 0.0044
*Notasterias* sp2	80	0.01108 ± 0.00109	14	0.718 ± 0.037	31	0.0114 ± 0.0096
*Diplasterias* sp1	105	0.01262 ± 0.00030	21	0.909 ± 0.013	32	0.0130 ± 0.0069
*Diplasterias* sp2	116	0.01547 ± 0.00097	19	0.849 ± 0.021	36	0.0161 ± 0.0123
*Diplasterias meridionalis*	75	0.00239 ± 0.00023	13	0.777 ± 0.031	11	0.0024 ± 0.0019
Broadcasters						
*Bathybiaster *sp1	133	0.00347 ± 0.00042	25	0.790 ± 0.023	29	0.0035 ± 0.0039
*Bathybiaster *sp2	110	0.00557 ± 0.00054	28	0.835 ± 0.026	34	0.0057 ± 0.0051
*Psilaster charcoti* clade 1	92	0.01457 ± 0.00069	29	0.924 ± 0.015	50	0.0151 ± 0.0085
*Psilaster charcoti* clade 2	55	0.00272 ± 0.00028	12	0.734 ± 0.052	12	0.0027 ± 0.0020
*Psilaster charcoti* clade 3	148	0.00160 ± 0.00026	19	0.394 ± 0.052	17	0.0016 ± 0.0025

### 3.1 Species delineation

**Figure 2:**
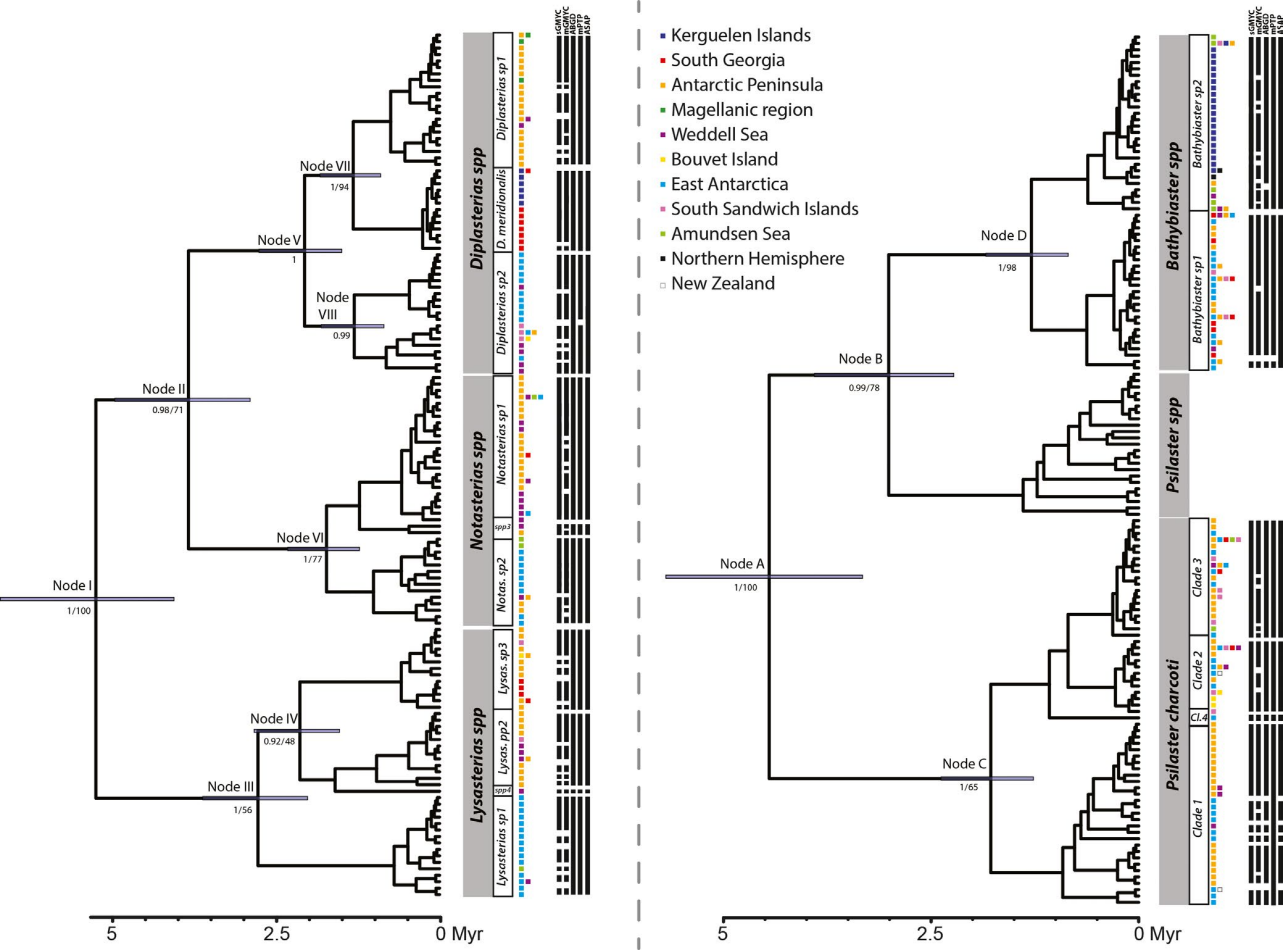
Bayesian chronograms of partitioned COI sequences derived from the brooding (left) and broadcasting (right) groups of interest. The distribution of uncertainty of node placement is indicated around each of the main nodes. Timescale is expressed in millions of years. Posterior probabilities and bootstrap values are provided under the main nodes. No value was indicated if bootstrap was <45%. Colored patches indicate sampling locations. Results for each species delineation method are reported as black bars representing the delineated units

**Figure 3:**
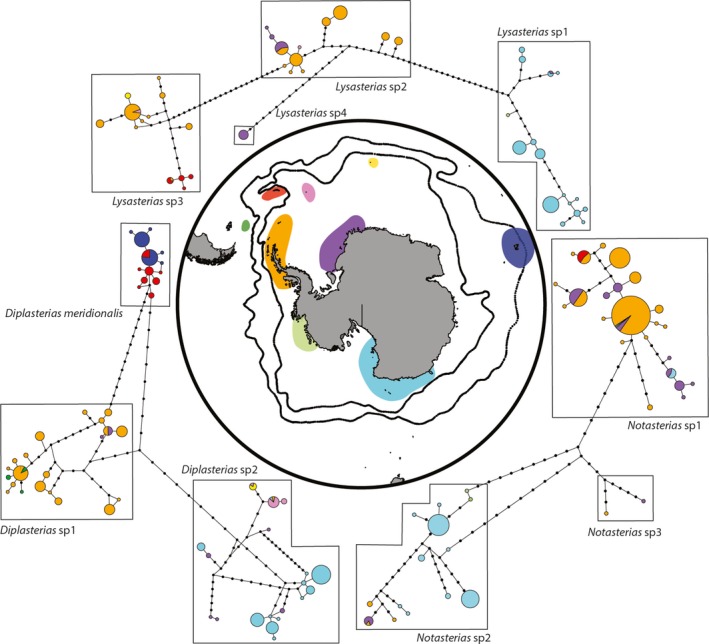
Brooders statistical parsimony network indicating genetic relationships with regards to sampling locations. Delineated clades are represented, and color code follows the central map: red—South Georgia; pink—South Sandwich Islands; yellow—Bouvet Island; dark blue—Kerguelen Islands; light blue—East Antarctica; light green—Amundsen Sea; orange—Antarctic Peninsula; dark green—Burdwood Bank; and purple—Weddell Sea. Projection: South Pole Stereographic

**Figure 4:**
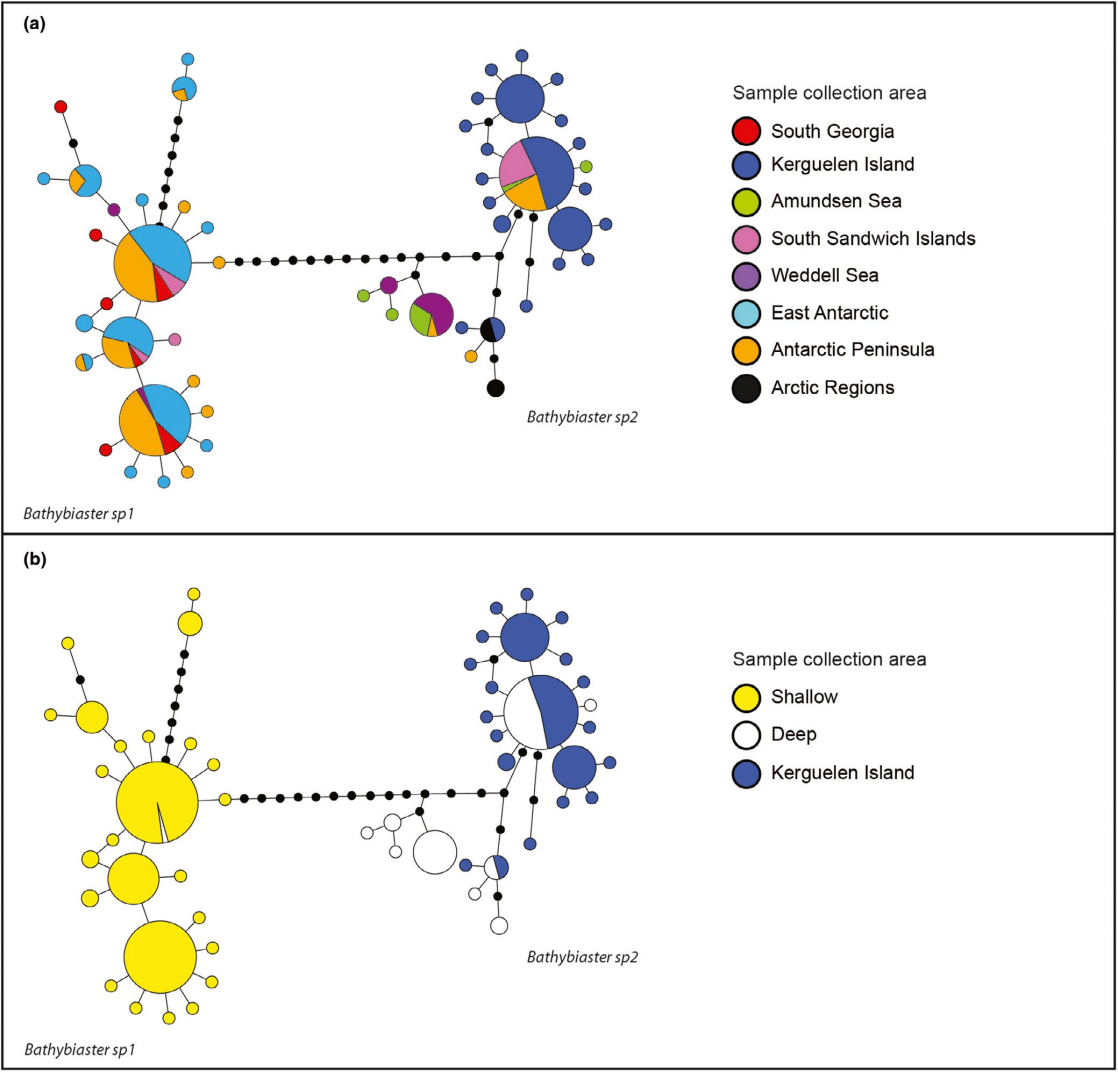
*Bathybiaster* statistical parsimony network indicating genetic relationships with regards to (a) sampling geographic locations and (b) sampling depths and Kerguelen Island (shallow and deep). Delineated clades are represented, and color code follows the appended legend

Species delineation applied to all genera shows contrasting results dependent upon the method applied (Figures 2, 3, 4; Table 2). Overall, the mGMYC method delineated the highest number of entities (79), while mPTP was the most conservative (19). Following a conservative approach, we based our work on the following delineated entities: two for *Bathybiaster*, three for *Diplasterias* and *Notasterias*, and four for *P. charcoti* and *Lysasterias*. These results are in line with haplotype networks and phylogenetic groupings (Figures 2, 3, 4; Appendix S2). Among the brooding entities, only one matches with an identified morphological species: *Diplasterias meridionalis*. In broadcasters, one delineated entity of the genus *Bathybiaster* comprises specimens from the Arctic identified as *Bathybiaster vexillifer* and *Psilaster andromeda*.

**Table 2:** Number of species delineated by the different species delineation methods

Taxonomic group	ABGD	sGMYC	mGMYC	mPTP	ASAP
Brooders					
*Lysasterias*	4	17	17	4	4
*Diplasterias*	3	14	15	4	3
*Notasterias*	3	4	18	3	3
Broadcasters					
*Psilaster charcoti*	9	9	17	4	9
*Bathybiaster*	4	3	12	3	2
Total	27	47	79	19	30

Genetic diversity of the delineated entities ranged from 0.00160 (*P. charcoti*—clade 3) to 0.01547 (*Diplasterias *sp2) for nucleotide diversity and from 0.394 (*P. charcoti*—clade 3) to 0.924 (*P. charcoti*—clade 1) for haplotype diversity (Table 1). The number of segregating sites varied from 11 (*D. meridionalis*) to 50 (*P. charcoti*—clade 1). Mean intraspecific distances varied from 0.16% (*P. charcoti*—clade 3) to 1.6% for *Diplasterias* sp2 (Table 1), while interspecific distances ranged from 2% between clades 2 and 3 (P. charcoti) to 6.8% between *Lysasterias* sp1 and *Lysasterias* sp4 (Appendix S2).

Haplotype and nucleotide diversity are not significantly different between brooders and broadcasters (Wilcoxon–Mann–Whitney tests; *p*‐values: 0.1709 and 0.9433, respectively).

### 3.2 Phylogenies and divergence time estimates

In total, 578 sequences in broadcasters and 838 in brooders were used for ML and BA reconstructions. Both methods give congruent results for broadcasters with similar taxonomic groupings and high node supports (Figure 2). Node support was higher using the BA method for brooders. Branching patterns were, however, identical in all reconstructions.

The monophyly of the genus *Psilaster* is not supported by our analysis, but SO *Psilaster* representatives are monophyletic (i.e., the nominal species *P. charcoti*). The genus *Bathybiaster* is monophyletic and includes specimens from the SO and from the Northern Hemisphere (Figure 2). *Psilaster* specimens collected outside the SO (*Psilaster acuminatus* from New Zealand and Australia, *Psilaster andromeda* from Sweden, and *Psilaster pectinatus* from the Arctic Ocean) are retrieved as monophyletic in a sister clade to *Bathybiaster*. The monophyly of each brooding genus is supported, but one subclade only matches with the morphological taxonomy: the species *D. meridionalis*. All other subclades are composed of specimens belonging to distinct morphospecies questioning current taxonomy at the species level.

Divergence time estimates suggest numerous divergence events both in brooders and in broadcasters over the last 5 Myr and, particularly, over the last 2.5 Myr (Figure 2). Results also indicate that members of the Asteriidae under study diverged from its Pan‐tropical outgroup around 21 Myr ago (Appendix S3). Main divergence events (Figure 2, Appendix S3) in *P. charcoti* and *Bathybiaster* occurred around 1.6 ± 0.8 Myr ago (Node C and D in Figure 2). In brooders, divergence time estimates (Figure 2, Appendix S3) between East Antarctic subclades and those of the Antarctic Peninsula all fall within the same time range of 2.1 ± 1.2 Ma (Nodes III, V, and VI in Figure 2). This is also in line with the time range computed for broadcasters.

### 3.3 Phylogeographic patterns

All brooders display clear geographic patterns, with a distinction between the East Antarctic and the Antarctic Peninsula (Figures 2, 3; Appendix S2). In *Diplasterias*, *D. meridionalis* displays a specific distribution as it is shared between South Georgia and the Kerguelen Plateau. *Diplasterias* sp1 includes specimens from the Antarctic Peninsula along with five specimens from the Magellanic region and five from the Weddell Sea. The last subclade (*Diplasterias* sp2) is mainly composed of specimens from the East Antarctic among which seven specimens from the Weddell Sea, 14 from the South Sandwich Islands, four from Bouvet Island, and one from the South Orkney Islands. For *Notasterias* (Table 2, Figures 2, 3; Appendix S2), one subclade (*Notasterias* sp1) mainly contains specimens from the Antarctic Peninsula and the Weddell Sea in addition with one sample from the Amundsen Sea, seven from South Georgia, and five from East Antarctica. Specimens belonging to the second subclade (*Notasterias* sp2) are mainly from East Antarctica (66 specimens) along with two from the Amundsen Sea, four from the Antarctic Peninsula, and five from the Weddell Sea. The remaining subclade (*Notasterias* sp3) only contains two deep‐sea specimens from the Scotia Arc (3,800 m) and the Weddell Sea (2,100 m). Within the genus *Lysasterias* (Table 2, Figures 2, 3; Appendix S2), the first entity (*Lysasterias* sp1) nearly exclusively contains specimens from East Antarctica with one from the Weddell Sea. The second entity (*Lysasterias* sp2) mainly contains specimens from the Antarctic Peninsula together with one specimen from the South Sandwich Islands and nine from the Weddell Sea. The third entity is mainly composed of specimens from the Antarctic Peninsula together with nine specimens from South Georgia, three from Bouvet Island, and two from the South Sandwich Islands. Finally, the last entity (*Lysasterias* sp4) is composed of nine specimens and is endemic to the Weddell Sea.

Broadcasters show contrasting biogeographic patterns. *P. charcoti* displays a complex haplotype network with subclade 4 endemic to Adélie Land and the three others circumpolar in distribution with two of them containing specimens collected off New Zealand (Appendix S2). Subclades of the genus *Bathybiaster* are mainly differentiated according to depth: *Bathybiaster* sp1 gathers specimens from the Antarctic shelf and the shallows of the Scotia Arc, along with a deep specimen from the South Sandwich Islands (Figure 4; Appendix S2). *Bathybiaster* sp2 is composed of representatives from the Antarctic slope, deep troughs, and canyons along with all specimens from the Kerguelen plateau and slope (Figure 4; Appendix S2).

## 4 DISCUSSION

### 4.1 Species richness, genetic diversity, and taxonomic implications

We found that genetic diversity did not significantly differ between brooders and broadcasters. This may reflect the presence of species complexes, related to recurrent disturbances. Recent glacial and interglacial cycles could have promoted high rates of differentiation in SO species (O'Hara, Hugall, Woolley, Bribiesca‐Contreras, & Bax, 2019), regardless of their dispersal capabilities. Discordances between the different delineation methods were detected in all groups. This had already been highlighted in recent studies (Blair & Bryson, 2017; Kekkonen, Mutanen, Kaila, Nieminen, & Hebert, 2015) and great care should be taken when interpreting the results, as further investigations are needed. At best, these methods should be considered as a first step toward subsequent and more integrative taxonomic works (Kekkonen et al., 2015), and therefore, a conservative approach is recommended (Carstens et al., 2013). Genetic diversity among the delineated species falls into the range of intraspecific values obtained in other taxa for COI mtDNA (Goodall‐Copestake et al., 2012), supporting this approach. Similarly, the measured interspecific (2%–6.8%) distances are within the range obtained in previous studies for sea stars (Foltz et al., 2013; Janosik et al., 2011). DNA barcoding has proven to be an efficient method to differentiate echinoderm species (Ward, Holmes, & O'Hara, 2008) with ~98% of the 191 studied species being distinguished based on their COI barcodes. Rapid diversification can, however, make intra‐ and interspecies genetic distances difficult to interpret using species delineation methods. The clear discrepancy between current taxonomy and our results emphasizes the urgent need for a complete reassessment of SO asteroid taxonomy based on multiple genes and an integrative taxonomic approach at both species and genus levels.

### 4.2 Dispersal capacity and biogeographic patterns

Several mechanisms have been proposed to account for the unusually high proportion of brooding species in the SO, and the evolutionary success of Antarctic brooders is now widely recognized within the community of Antarctic biologists (Poulin, Palma, & Féral, 2002). While adaptation to polar environmental conditions is not considered a determining mechanism (Pearse, Mooi, Lockhart, & Brandt, 2009; Poulin et al., 2002), reproductive strategy has been hypothesized as the main factor structuring the evolution and the diversity of SO benthic life (Raupach et al., 2010; Thatje, 2012). Whether they are based on morphology (Moreau et al., 2017) or genetic markers (Hoffman et al., 2011), most studies agree that biogeographic structures are more pronounced in brooders than in broadcasters due to contrasting dispersal capacities.

Most of the results obtained in the present study are in line with this expected biogeographic pattern, with the prevalence of clear spatial structures in brooders. This is in agreement with our previous work describing the SO asteroid biogeographic patterns and demonstrating the importance of life history traits to understanding the structure of spatial distributions (Moreau et al., 2017). Our results support the differentiation of species between the Antarctic Peninsula and the East Antarctic. The past collapse of the West Antarctic Ice Sheet (Bamber, Riva, Vermeersen, & LeBrocq, 2009; Pollard & DeConto, 2009) and the putative existence of a subsequent trans‐Antarctic seaway separating the West and the East Antarctic have been proposed as possible mechanisms leading to such patterns (Barnes & Hillenbrand, 2010; Linse, Griffiths, Barnes, & Clarke, 2006; Pierrat, Saucède, Brayard, & David, 2013). The role of the Weddell Sea Gyre (Linse et al., 2006), environmental dissimilarities, and contrasting glacial histories have also been proposed as possible explanations (Anderson, Shipp, Lowe, Wellner, & Mosola, 2002). Interestingly, in brooding genera, haplotypes of specimens from the Weddell Sea are present in both the East Antarctic and the Antarctic Peninsula clades. Recent faunal exchanges between the two regions could explain the occurrence in the Weddell Sea of a mixed fauna of East Antarctic and Antarctic Peninsula origin as the Weddell Sea is located in between these two regions.

Divergence dates suggest that a vicariance event occurred ~2 Myr ago, while the last collapse of the West Antarctic ice sheet is hypothesized to have occurred more recently, in the last 1.1 Myr (Pollard & DeConto, 2009; Scherer et al., 2008). This last dating is in line with previous work on SO asteroids (Janosik et al., 2011). Contrasting divergence dating between phylogeographic studies is not uncommon and absolute ages should be taken with caution when using a molecular clock (Thomas, Welch, Woolfit, & Bromham, 2006), given the uncertainties in molecular divergence rates, especially when a single locus is used and when fossil calibrations are not available. However, similar divergence dates were obtained for all brooding genera, which strongly suggests the effect of a common and significant event. Unfortunately, only a few abyssal samples were available for study (only two for the entity *Notasterias* sp3). They are strongly divergent from all other *Notasterias* specimens, suggesting the absence of population mixing and independent evolution of populations of *Notasterias* both on the continental shelf and in the deep sea.

In contrast to the marked genetic structures described above, exceptions have been in certain brooders due to the effect of unusual dispersal vectors reported (Cumming, Nikula, Spencer, & Waters, 2014; Díaz et al., 2011). This is also the case in this study, as the brooding species *D. meridionalis* shares genetic units between South Georgia and the Kerguelen Islands, which suggests an ongoing connectivity between the two distant regions. This pattern could be explained by kelp rafting via the Antarctic Circumpolar Current (ACC), which has previously been found in other SO brooding echinoderms (O'hara, 1998) and taxa such as in isopods and mollusks (González‐Wevar et al., 2018; Leese, Agrawal, & Held, 2010). Kelp rafting was suggested as a viable dispersal vector for shallow‐water species living in macro‐algal beds (O'hara, 1998), which is the case of *D. meridionalis*.

Genetic structures in broadcasters under study are quite different from the patterns observed in brooders. The studied broadcasters show circumpolar structures that suggest the prevalence of gene flow across the SO promoted by higher larval dispersal capacities. Geographic patterns in *P. charcoti*, with several circumpolar entities, suggest the existence of past refugia during past glacial maxima and subsequent dispersal centers (Hemery et al., 2012). Unfortunately, little is known about asteroid larval biology in the SO, apart from a handful of well‐studied species (Agüera, Collard, Jossart, Moreau, & Danis, 2015; Pearse, McClintock, & Bosch, 1991; Peck, Souster, & Clark, 2013; Souster, Morley, & Peck, 2018). Major differences exist between the genetic structure of *Psilaster* and *Bathybiaster*: (a) Clades of *P. charcoti* are found to have a greatly fragmented pattern, potentially as a result of lower dispersal capacity than representatives of *B. loripes* (found at continental shelf depths), but this could also reflect a longer evolutionary history as these clades are older (Figure 2), (b) some specimens of *P. charcoti* occur on both sides of the PF, and (c) *Bathybiaster* sp2 shows a potential bipolar distribution (sensu Darling et al., 2000) and likely corresponds to the nominal species *B. vexillifer*. The occurrence of shared haplotypes between the Northern and Southern Hemispheres in *Bathybiaster* sp2 suggests the existence of recent gene flow between the two hemispheres. Morphological similarities between North American and South African specimens had already been indicated in previous work on the echinoderm fauna of South Africa (Clark, 1923). This could be the result of a deep‐sea dispersal route, a scenario coined the “thermohaline expressway” by Strugnell, Rogers, Prodöhl, Collins, and Allcock (2008), but a wide, cosmopolitan distribution of the species is probably the most reasonable hypothesis. Unfortunately, no tropical deep‐sea samples were available for study. *Bathybiaster* sp2 is recorded on the Kerguelen Plateau around the Kerguelen Islands at shallow depths, giving credit to a possible sub‐Antarctic emergence scenario and colonization of the Kerguelen Plateau from the deep sea (Aronson et al., 2007; Díaz et al., 2011).

Interpretation of the present results is limited by the use of a single mtDNA locus. The observed patterns could be due to mechanisms such as adaptive introgression, demographic disparities, or sex‐biased asymmetries arising from the sole use of mtDNA (Toews & Brelsford, 2012). Furthermore, if data derived from COI analyses reflect a long‐term effect of contrasting dispersal capacities, other fast‐evolving nuclear markers such as microsatellites or SNPs (RAD‐Seq data) will be needed in the future to investigate these recent and ongoing processes. Nevertheless, the causal relationship between genetic patterns and dispersal capacities of asteroids can be linked to past climatic and geological events and give some clues to the upstream drivers of species evolution.

### 4.3 The role of past climate change

The SO has been partially isolated for nearly 40 million years since the first opening of the Drake Passage, which led to the onset of the ACC and subsequent cooling of the ocean when the ACC intensified. However, isolation of the SO was reduced several times during periods of climate warming and the decreasing ACC intensity (Dalziel et al., 2013; Lagabrielle, Goddéris, Donnadieu, Malavieille, & Suarez, 2009). In a recent review of key stages in the evolution of the Antarctic marine fauna, Crame (2018) emphasizes the influence of past climate on the current distribution of modern fauna. At the scale of the Cenozoic (c. 65 Myr), he points out that the succession of several major extinction and radiation events are linked to important drops in seawater temperature. The fossil record of SO sea stars is very poor, but it suggests that the extinction of an older asteroid fauna was followed by the diversification of the Antarctic Asteriidae and of other modern Antarctic forcipulataceans (Mah & Foltz, 2011b). The origin of the Antarctic Asteriidae has been dated to 21 Ma in this study, suggesting a diversification of the family starting in the Miocene after the initial opening of the Drake passage (c. 34 Ma) and before the full establishment of the ACC (c. 14 Ma) (Lawver & Gahagan, 2003). The global phylogeny of Astropectinidae, reconstructed by Mah and Foltz (2011a), does not show any regional pattern, which makes any speculation as to the origin of *Bathybiaster* and *Psilaster* in the SO problematic. Our results, however, suggest a recent evolutionary history of broadcasting genera in the SO (c. 5 Myr). At a more recent timescale, all the studied groups show a high diversification rate over the last ~2.5 Myr, suggesting that recent climate events could account for these observed patterns. Naish et al. (2009) estimated that at least 38 distinct glacial cycles occurred over the last 5 Myr. These glacial cycles are believed to have been major drivers of species differentiation as hypothesized by the “Antarctic diversity pump” hypothesis (Clarke & Crame, 1989, 1992) and even promoting evolutionary radiations of the benthic fauna (Allcock, 2005; Raupach, Malyutina, Brandt, & Wägele, 2007; Wilson, Schrödl, & Halanych, 2009). These major environmental changes could have also resulted in cryptic speciation in brooding species and broadcasting species with limited larval dispersal capabilities, by the maintenance of genetic differentiation at local scales (Thatje, 2012). Finally, the ACC hypothesis predicts the “existence of many species in clades of varied divergence times, at a wide range of depths, but with highest diversity downstream of the Drake Passage, in the Scotia Arc and Weddell Sea” (sic. Pearse et al., 2009). This hypothesis could explain some of the diversification patterns we have observed in asteroids. Nevertheless, the apparent correlation with past geological and climatic events should be taken with great care due to the lack of fossil records and uncertainties in calibration of the molecular clock also known to be taxon‐ and climate regime‐dependent (Gillooly, Allen, West, & Brown, 2005).

### 4.4 Conclusion and future prospects

The observed genetic patterns of SO sea stars highlight the relevance of considering life history traits to understand spatial patterns of genetic diversity. Our results suggest that reproductive strategy could have conditioned the spatial structuring of genetic diversity, but with no apparent effect on the level of genetic diversity. Molecular results also indicate that an in‐depth taxonomic revision of the group is needed based on an integrative taxonomy approach (combining genetic and morphological data). The increasing development of genetic surveys in biodiversity conservation and management plans (Goodall‐Copestake et al., 2012) stresses the need for robust estimates of species diversity. Species complexes are frequent in the SO (Hemery et al., 2012), and estimates of species diversity based on morphology can lead to under‐ or alternatively, overestimating “true” biological diversity. In that context, multilocus approaches are essential to detect evolutionary processes within species. Finally, the apparent bipolar or cosmopolitan distribution of the species *B. vexillifer* should be further investigated using additional specimens and multiple molecular markers. This will bring new insights on a potential deep‐sea route connecting the two polar oceans and the relevance of biogeographic scenarios such as the thermohaline expressway (Strugnell et al., 2008) and the sub‐Antarctic emergence (Díaz et al., 2011). The presence of *B. loripes* on the continental shelf could be the result of a past colonization from the deep sea (Strugnell et al., 2011), which potentially challenges the established paradigm of an isolated SO benthic diversity (Clarke, Aronson, Crame, Gili, & Blake, 2004).

## CONFLICT OF INTEREST

None declared.

## AUTHOR CONTRIBUTIONS

C.M., Q.J., B.D., and T.S. conceived and designed the experiments; C.M., Q.J., C.S., and G.A. performed the analyses; C.M., Q.J., A.A., M.E., B.D., and T.S. collected the data; and C.M., B.D., Q.J., M.E., C.S., G.A., A.A., and T.S. wrote the manuscript.

## Data Availability

All data and metadata used in this study are publicly available on BOLD Systems (www.boldsystems.org) under the Dataset Code DS‐ASTROULB as a compilation of the following projects: SEAST; EAR; DSPEC; CHEC; SOA; SWEMA; TNBA; TCTNB; ODTNB; AIIIS; SORK; NZEC; and NZECA.
