# Supplementary material for: Is reproductive strategy a key factor in understanding the evolutionary history of Southern Ocean Asteroidea (Echinodermata)?
Source: Ecol Evol. 2019 Jul 16;9(15):8465–78. doi: 10.1002/ece3.5280 (PMC6686340; doi:10.1002/ece3.5280)
Supplement: Supplementary file 1 [file ECE3-9-8465-s001.docx]

*Journal of Biogeography*

**SUPPORTING INFORMATION**

**Reproductive strategy helps resolving the evolutionary history of Southern Ocean Asteroidea (Echinodermata)**

Camille Moreau, Bruno Danis, Quentin Jossart, Marc Eléaume, Chester Sands, Guillaume Achaz, Antonio Agüera, and Thomas Saucède

Appendix S1 Associated metadata for each COI sequence together with the Barcode of Life Data System ID. Locations are displayed in decimal degrees and as region. Abbreviations: Museo Nazionale dell’Antartide (MNA); Museum National d’Histoire Naturelle (MNHN); California Academy of Sciences (CAS); Ross Sea (Ross); Kerguelen Island (Ker) ; Elephant Island (Eleph) ; South Georgia (SG) ; Bouvet Island (Bouv) ; South Shetland (Sshet) ; South Orkneys (Sork) ; South Sandwich Islands (Ssand); ADélie Land (AdL); Weddell Sea (WS); Eastern Part of the Antarctic Peninsula (AntPenEast); Shag Rocks (Shag); Bellingshausen Sea (Belli); Balleny Islands (Bal); Magellanic (Mag); Amundsen Sea (Amund); Australia (Aus); New Zealand (NZ).

| **BOLD_ID** | **Sequence_code** | **Cruise/Expedition** | **ID** | **Identified by** | **Museum_voucher** | **Year** | **Lat** | **Long** | **Region** | **Depth** |
| --- | --- | --- | --- | --- | --- | --- | --- | --- | --- | --- |
| **Dip003** | **Dip003** | **XXV** | **Diplasterias brucei** | **Camille Moreau** | **MNA_2719** | **2009** | **-74.69** | **164.10** | **Ross** | **20-30** |
| **Dip004** | **Dip004** | **XXV** | **Diplasterias brucei** | **Camille Moreau** | **MNA_2719** | **2009** | **-74.69** | **164.10** | **Ross** | **20-30** |
| **Dip005** | **Dip005** | **XXV** | **Diplasterias brucei** | **Camille Moreau** | **MNA_2719** | **2009** | **-74.69** | **164.10** | **Ross** | **20-30** |
| **Dip006** | **Dip006** | **XXV** | **Diplasterias brucei** | **Camille Moreau** | **MNA_2719** | **2009** | **-74.69** | **164.10** | **Ross** | **20-30** |
| **Dip007** | **Dip007** | **XXV** | **Diplasterias brucei** | **Camille Moreau** | **MNA_2719** | **2009** | **-74.69** | **164.10** | **Ross** | **20-30** |
| **Dip008** | **Dip008** | **XXV** | **Diplasterias brucei** | **Camille Moreau** | **MNA_2719** | **2009** | **-74.69** | **164.10** | **Ross** | **20-30** |
| **TNBA032-15** | **Dip010** | **XXV** | **Diplasterias brucei** | **Camille Moreau** | **MNA_2741** | **2009** | **-74.69** | **164.10** | **Ross** | **20-30** |
| **TNBA020-15** | **Dip011** | **XXV** | **Diplasterias brucei** | **Camille Moreau** | **MNA_2900** | **2010** | **-74.70** | **164.12** | **Ross** | **20** |
| **Dip012** | **Dip012** | **XXV** | **Diplasterias brucei** | **Camille Moreau** | **MNA_2901** | **2010** | **-74.70** | **164.12** | **Ross** | **20** |
| **TCTNB051-15** | **Dip013** | **XXV** | **Diplasterias brucei** | **Camille Moreau** | **MNA_3402** | **2009** |  |  | **Ross** | **30** |
| **TNBA019-15** | **Dip014** | **XXVII** | **Diplasterias brucei** | **Camille Moreau** | **MNA_3432** | **2012** | **-74.70** | **164.13** | **Ross** |  |
| **Dip017** | **Dip017** | **XXVII** | **Diplasterias brucei** | **Camille Moreau** | **MNA_3512** |  |  |  | **Ross** |  |
| **Dip019** | **Dip019** | **XXVII** | **Diplasterias brucei** | **Camille Moreau** | **MNA_3698.1** | **2012** | **-74.70** | **164.13** | **Ross** |  |
| **Dip020** | **Dip020** | **XXVII** | **Diplasterias brucei** | **Camille Moreau** | **MNA_3698.2** | **2012** | **-74.70** | **164.13** | **Ross** |  |
| **Dip021** | **Dip021** | **XXVII** | **Diplasterias brucei** | **Camille Moreau** | **MNA_3698.3** | **2012** | **-74.70** | **164.13** | **Ross** |  |
| **Dip023** | **Dip023** | **XXVII** | **Diplasterias brucei** | **Camille Moreau** | **MNA_3698.5** | **2012** | **-74.70** | **164.13** | **Ross** |  |
| **Dip025** | **Dip025** | **XXVII** | **Diplasterias brucei** | **Camille Moreau** | **MNA_3698.7** | **2012** | **-74.70** | **164.13** | **Ross** |  |
| **Dip026** | **Dip026** | **XXVII** | **Diplasterias brucei** | **Camille Moreau** | **MNA_3791** | **2012** | **-74.71** | **164.15** | **Ross** |  |
| **Dip027** | **Dip027** | **XXVII** | **Diplasterias brucei** | **Camille Moreau** | **MNA_3818.1** | **2012** | **-74.71** | **164.15** | **Ross** |  |
| **Dip028** | **Dip028** | **XXVII** | **Diplasterias brucei** | **Camille Moreau** | **MNA_3818.2** | **2012** | **-74.71** | **164.15** | **Ross** |  |
| **Dip029** | **Dip029** | **XXVII** | **Diplasterias brucei** | **Camille Moreau** | **MNA_3885** | **2012** | **-74.71** | **164.15** | **Ross** |  |
| **TCTNB059-15** | **Dip030** | **XXVII** | **Diplasterias brucei** | **Camille Moreau** | **MNA_3895** |  |  |  | **Ross** | **25** |
| **Dip016** | **Dip016** | **XXVII** | **Diplasterias brucei** | **Camille Moreau** | **MNA_4292** |  |  |  | **Ross** |  |
| **Dip015** | **Dip015** | **XXVII** | **Diplasterias brucei** | **Camille Moreau** | **MNA_4293** | **2012** | **-74.70** | **164.06** | **Ross** |  |
| **Dip018** | **Dip018** | **XXVII** | **Diplasterias brucei** | **Camille Moreau** | **MNA_4294** |  |  |  | **Ross** |  |
| **TCTNB054-15** | **Lys008** | **XXVIII** | **Lysasterias sp** | **Camille Moreau** | **MNA_5753** | **2013** |  |  | **Ross** | **78** |
| **Dip126** | **Dip126** | **Proteker II** | **Undet** | **Camille Moreau** |  | **2013** | **-48.41** | **69.04** | **Ker** | **51** |
| **Dip430** | **Dip430** | **Proteker I** | **Undet** | **Camille Moreau** |  | **2011** | **-49.23** | **69.56** | **Ker** | **15** |
| **Dip122** | **Dip122** | **Proteker II** | **Undet** | **Camille Moreau** |  | **2013** | **-49.23** | **69.56** | **Ker** | **17** |
| **Dip127** | **Dip127** | **Proteker II** | **Undet** | **Camille Moreau** |  | **2013** | **-49.23** | **69.56** | **Ker** | **17** |
| **Lys337** | **Lys337** | **CASIZ** | **Lysasterias perrieri** | **Christopher L. Mah** | **CAS 163016** | **2002** | **-61.02** | **-55.98** | **Eleph** | **338** |
| **Lys335** | **Lys335** | **CASIZ** | **Lysasterias belgicae** | **Christopher L. Mah** | **CAS 173600** | **2004** | **-53.77** | **-38.22** | **SG** | **205** |
| **Lys343** | **Lys343** | **CASIZ** | **Lysasterias perrieri** | **Christopher L. Mah** | **CAS 174630** | **2004** | **-54.36** | **3.17** | **Bouv** | **455** |
| **Lys336** | **Lys336** | **CASIZ** | **Lysasterias perrieri** | **Christopher L. Mah** | **CAS 174636** | **2004** | **-54.36** | **3.17** | **Bouv** | **441** |
| **Dip421** | **Dip421** | **CASIZ** | **Diplasterias brucei** | **Christopher L. Mah** | **CAS 174668** | **2006** | **-62.83** | **-57.46** | **Bransfield** | **132** |
| **Lys347** | **Lys347** | **CASIZ** | **Lysasterias perrieri** | **Christopher L. Mah** | **CAS 174678** | **2006** | **-62.25** | **-59.43** | **Sshet** | **762** |
| **Lys340** | **Lys340** | **CASIZ** | **Lysasterias perrieri** | **Christopher L. Mah** | **CAS 174691** | **2006** | **-62.41** | **-54.22** | **Bransfield** | **415** |
| **Dip420** | **Dip420** | **CASIZ** | **Diplasterias brucei** | **Christopher L. Mah** | **CAS 175996** | **2006** | **-62.65** | **-55.61** | **Bransfield** | **177** |
| **Lys329** | **Lys329** | **CASIZ** | **Lysasterias sp** | **Christopher L. Mah** | **CAS 176003** | **2006** | **-62.52** | **-55.99** | **Bransfield** | **229** |
| **Lys332** | **Lys332** | **CASIZ** | **Lysasterias sp** | **Christopher L. Mah** | **CAS 180746** | **2009** | **-60.77** | **-46.27** | **Sork** | **146** |
| **Lys330** | **Lys330** | **CASIZ** | **Lysasterias sp** | **Christopher L. Mah** | **CAS 180747** | **2009** | **-60.60** | **-44.76** | **Sork** | **105** |
| **Lys326** | **Lys326** | **CASIZ** | **Lysasterias sp** | **Christopher L. Mah** | **CAS 180751** | **2009** | **-60.61** | **-45.15** | **Sork** | **92** |
| **Lys327** | **Lys327** | **CASIZ** | **Lysasterias sp** | **Christopher L. Mah** | **CAS 180769** | **2009** | **-61.01** | **-44.90** | **Sork** | **265** |
| **Lys323** | **Lys323** | **CASIZ** | **Lysasterias sp** | **Christopher L. Mah** | **CAS 180770** | **2009** | **-61.05** | **-46.82** | **Sork** | **772** |
| **Lys346** | **Lys346** | **CASIZ** | **Lysasterias perrieri** | **Christopher L. Mah** | **CAS 174595b** | **2004** | **-57.05** | **-26.74** | **Ssand** | **127** |
| **Lys341** | **Lys341** | **CASIZ** | **Lysasterias perrieri** | **Christopher L. Mah** | **CAS 174595c** | **2004** | **-57.05** | **-26.74** | **Ssand** | **127** |
| **Lys318** | **Lys318** | **CASIZ** | **Lysasterias sp** | **Christopher L. Mah** | **CAS 175977a** | **2006** | **-62.15** | **-54.58** | **Bransfield** | **778** |
| **Lys325** | **Lys325** | **CASIZ** | **Lysasterias sp** | **Christopher L. Mah** | **CAS 175977b** | **2006** | **-62.15** | **-54.58** | **Bransfield** | **778** |
| **Lys319** | **Lys319** | **CASIZ** | **Lysasterias sp** | **Christopher L. Mah** | **CAS 175984a** | **2006** | **-62.26** | **-60.79** | **Sshet** | **290** |
| **Lys328** | **Lys328** | **CASIZ** | **Lysasterias sp** | **Christopher L. Mah** | **CAS 175984b** | **2006** | **-62.26** | **-60.79** | **Sshet** | **290** |
| **Lys320** | **Lys320** | **CASIZ** | **Lysasterias sp** | **Christopher L. Mah** | **CAS 180767a** | **2009** | **-63.02** | **-52.37** | **TipPen** | **628** |
| **Lys324** | **Lys324** | **CASIZ** | **Lysasterias sp** | **Christopher L. Mah** | **CAS 180767b** | **2009** | **-63.02** | **-52.37** | **TipPen** | **628** |
| **ASTAN332-10** |  | **CEAMARC** | **Juvenile** | **Christopher L. Mah** | **MNHN IE_2009_6874** | **2008** |  |  | **AdL** |  |
| **ASTAN348-10** |  | **CEAMARC** | **Juvenile** | **Christopher L. Mah** | **MNHN IE_2009_7189** | **2008** |  |  | **AdL** |  |
| **ASTAN387-10** |  | **CEAMARC** | **Juvenile** | **Christopher L. Mah** | **MNHN IE_2009_7247** | **2008** |  |  | **AdL** |  |
| **ASTAN397-10** |  | **CEAMARC** | **Juvenile** | **Christopher L. Mah** | **MNHN IE_2009_7258** | **2008** |  |  | **AdL** |  |
| **Not317** | **Not317** | **CEAMARC** | **Notasterias sp** | **Christopher L. Mah** | **MNHN IE-2005-9021** | **2008** |  |  | **AdL** |  |
| **Not318** | **Not318** | **CEAMARC** | **Notasterias sp** | **Christopher L. Mah** | **MNHN IE-2005-9022** | **2008** |  |  | **AdL** |  |
| **Not319** | **Not319** | **CEAMARC** | **Notasterias sp** | **Christopher L. Mah** | **MNHN IE-2005-9023** | **2008** |  |  | **AdL** |  |
| **Not304** | **Not304** | **CEAMARC** | **Notasterias brachiata** | **Christopher L. Mah** | **MNHN IE-2009-6748** | **2008** | **-66.34** | **140.03** | **AdL** | **509.9** |
| **ASTAN307-10** | **Bat036** | **CEAMARC** | **Bathybiaster loripes** | **Christopher L. Mah** | **MNHN IE-2009-6757** | **2008** | **-66.41** | **140.51** | **AdL** | **971.5** |
| **Lys234** | **Lys234** | **CEAMARC** | **Lysasterias adeliae** | **Christopher L. Mah** | **MNHN IE-2009-6760** | **2008** | **-66.39** | **140.43** | **AdL** | **791.5** |
| **Lys228** | **Lys228** | **CEAMARC** | **Lysasterias adeliae** | **Christopher L. Mah** | **MNHN IE-2009-6772** | **2008** | **-66.33** | **140.65** | **AdL** | **164.9** |
| **ASTAN326-10** | **Psi069** | **CEAMARC** | **Psilaster charcoti** | **Christopher L. Mah** | **MNHN IE-2009-6778** | **2008** | **-65.99** | **139.31** | **AdL** | **472** |
| **ASTAN332-10** | **Psi045** | **CEAMARC** | **Psilaster charcoti** | **Christopher L. Mah** | **MNHN IE-2009-6784** | **2008** | **-65.44** | **139.32** | **AdL** | **1231** |
| **Not344** | **Not344** | **CEAMARC** | **Notasterias armata** | **Christopher L. Mah** | **MNHN IE-2009-6788** | **2008** | **-65.44** | **139.32** | **AdL** | **1231** |
| **ASTAN001-10** | **Bat019** | **CEAMARC** | **Bathybiaster loripes** | **Christopher L. Mah** | **MNHN IE-2009-6789** | **2008** | **-66.42** | **140.49** | **AdL** | **1017.8** |
| **ASTAN002-10** | **Bat017** | **CEAMARC** | **Bathybiaster loripes** | **Christopher L. Mah** | **MNHN IE-2009-6790** | **2007** | **-66.87** | **142.68** | **AdL** | **396.4** |
| **ASTAN003-10** | **Psi145** | **CEAMARC** | **Psilaster charcoti** | **Christopher L. Mah** | **MNHN IE-2009-6791** | **2007** | **-66.01** | **142.68** | **AdL** | **432.8** |
| **ASTAN004-10** | **Psi139** | **CEAMARC** | **Bathybiaster loripes** | **Christopher L. Mah** | **MNHN IE-2009-6792** | **2007** | **-66.87** | **142.68** | **AdL** | **396.4** |
| **Not291** | **Not291** | **CEAMARC** | **Notasterias bongraini** | **Christopher L. Mah** | **MNHN IE-2009-6793** | **2007** | **-66.75** | **145.53** | **AdL** | **526.2** |
| **ASTAN008-10** | **Bat045** | **CEAMARC** | **Bathybiaster loripes** | **Christopher L. Mah** | **MNHN IE-2009-6799** | **2008** | **-66.54** | **144.65** | **AdL** | **574.9** |
| **Not274** | **Not274** | **CEAMARC** | **Notasterias armata** | **Christopher L. Mah** | **MNHN IE-2009-6801** | **2008** | **-66.54** | **144.65** | **AdL** | **574.9** |
| **Lys233** | **Lys233** | **CEAMARC** | **Lysasterias joffrei** | **Christopher L. Mah** | **MNHN IE-2009-6803** | **2008** | **-66.54** | **144.65** | **AdL** | **574.9** |
| **ASTAN026-10** | **Bat048** | **CEAMARC** | **Bathybiaster loripes** | **Christopher L. Mah** | **MNHN IE-2009-6818** | **2007** | **-66.01** | **142.72** | **AdL** | **433.28** |
| **ASTAN041-10** | **Psi050** | **CEAMARC** | **Psilaster charcoti** | **Christopher L. Mah** | **MNHN IE-2009-6835** | **2007** | **-66.57** | **143.38** | **AdL** | **810.4** |
| **Psi051** | **Psi051** | **CEAMARC** | **Psilaster charcoti** | **Christopher L. Mah** | **MNHN IE-2009-6836** | **2007** | **-66.57** | **143.38** | **AdL** | **810.4** |
| **Not346** | **Not346** | **CEAMARC** | **Notasterias stolophora** | **Christopher L. Mah** | **MNHN IE-2009-6837** | **2007** | **-66.75** | **145.53** | **AdL** | **526.2** |
| **Lys247** | **Lys247** | **CEAMARC** | **Lysasterias cf. lactea** | **Christopher L. Mah** | **MNHN IE-2009-6852** | **2007** | **-66.75** | **144.96** | **AdL** | **660.9** |
| **Not321** | **Not321** | **CEAMARC** | **Notasterias armata** | **Christopher L. Mah** | **MNHN IE-2009-6853** | **2007** | **-66.75** | **144.96** | **AdL** | **660.9** |
| **Not350** | **Not350** | **CEAMARC** | **Notasterias armata** | **Christopher L. Mah** | **MNHN IE-2009-6854** | **2007** | **-66.75** | **144.96** | **AdL** | **660.9** |
| **Lys256** | **Lys256** | **CEAMARC** | **Lysasterias cf. lactea** | **Christopher L. Mah** | **MNHN IE-2009-6857** | **2007** | **-66.75** | **144.96** | **AdL** | **660.9** |
| **Psi052** | **Psi052** | **CEAMARC** | **Psilaster charcoti** | **Christopher L. Mah** | **MNHN IE-2009-6863** | **2007** | **-66.75** | **144.96** | **AdL** | **660.9** |
| **Bat033** | **Bat033** | **CEAMARC** | **Bathybiaster loripes** | **Christopher L. Mah** | **MNHN IE-2009-6864** | **2007** | **-66.75** | **144.96** | **AdL** | **660.9** |
| **ASTAN064-10** | **Bat050** | **CEAMARC** | **Bathybiaster loripes** | **Christopher L. Mah** | **MNHN IE-2009-6865** | **2007** | **-66.00** | **143.72** | **AdL** | **425.6** |
| **ASTAN065-10** | **Bat049** | **CEAMARC** | **Bathybiaster loripes** | **Christopher L. Mah** | **MNHN IE-2009-6866** | **2007** | **-66.55** | **142.96** | **AdL** | **866.6** |
| **Dip312** | **Dip312** | **CEAMARC** | **Diplasterias brucei** | **Christopher L. Mah** | **MNHN IE-2009-6879** | **2007** | **-67.05** | **145.15** | **AdL** | **1266.6** |
| **ASTAN086-10** | **Psi049** | **CEAMARC** | **Psilaster charcoti** | **Christopher L. Mah** | **MNHN IE-2009-6895** | **2007** | **-66.05** | **142.76** | **AdL** | **451.76** |
| **ASTAN097-10** | **Bat055** | **CEAMARC** | **Bathybiaster loripes** | **Christopher L. Mah** | **MNHN IE-2009-6912** | **2007** | **-66.00** | **143.72** | **AdL** | **425.6** |
| **Bat056** | **Bat056** | **CEAMARC** | **Bathybiaster loripes** | **Christopher L. Mah** | **MNHN IE-2009-6913** | **2007** | **-66.00** | **143.72** | **AdL** | **425.6** |
| **Bat024** | **Bat024** | **CEAMARC** | **Bathybiaster loripes** | **Christopher L. Mah** | **MNHN IE-2009-6916** | **2007** | **-66.34** | **143.04** | **AdL** | **683.6** |
| **ASTAN105-10** | **Bat020** | **CEAMARC** | **Bathybiaster loripes** | **Christopher L. Mah** | **MNHN IE-2009-6925** | **2007** | **-66.56** | **142.39** | **AdL** | **383.9** |
| **Not279** | **Not279** | **CEAMARC** | **Notasterias armata** | **Christopher L. Mah** | **MNHN IE-2009-6927** | **2007** | **-66.56** | **142.39** | **AdL** | **383.9** |
| **Not307** | **Not307** | **CEAMARC** | **Notasterias brachiata** | **Christopher L. Mah** | **MNHN IE-2009-6935** | **2007** | **-66.75** | **145.21** | **AdL** | **597.2** |
| **ASTAN115-10** | **Bat051** | **CEAMARC** | **Bathybiaster loripes** | **Christopher L. Mah** | **MNHN IE-2009-6936** | **2007** | **-66.75** | **145.21** | **AdL** | **597.2** |
| **ASTAN117-10** | **Psi053** | **CEAMARC** | **Psilaster charcoti** | **Christopher L. Mah** | **MNHN IE-2009-6939** | **2007** | **-66.55** | **142.96** | **AdL** | **866.6** |
| **Psi054** | **Psi054** | **CEAMARC** | **Psilaster charcoti** | **Christopher L. Mah** | **MNHN IE-2009-6940** | **2007** | **-66.55** | **142.96** | **AdL** | **866.6** |
| **Psi055** | **Psi055** | **CEAMARC** | **Psilaster charcoti** | **Christopher L. Mah** | **MNHN IE-2009-6940** | **2007** | **-66.55** | **142.96** | **AdL** | **866.6** |
| **ASTAN122-10** | **Bat034** | **CEAMARC** | **Bathybiaster loripes** | **Christopher L. Mah** | **MNHN IE-2009-6946** | **2007** | **-66.01** | **142.68** | **AdL** | **432.8** |
| **ASTAN126-10** | **Psi070** | **CEAMARC** | **Psilaster charcoti** | **Christopher L. Mah** | **MNHN IE-2009-6952** | **2007** | **-66.01** | **142.68** | **AdL** | **432.8** |
| **ASTAN128-10** | **Psi067** | **CEAMARC** | **Psilaster charcoti** | **Christopher L. Mah** | **MNHN IE-2009-6955** | **2007** | **-66.01** | **142.68** | **AdL** | **432.8** |
| **Psi073** | **Psi073** | **CEAMARC** | **Psilaster charcoti** | **Christopher L. Mah** | **MNHN IE-2009-6971** | **2008** | **-66.54** | **143.99** | **AdL** | **786.9** |
| **Psi075** | **Psi075** | **CEAMARC** | **Psilaster charcoti** | **Christopher L. Mah** | **MNHN IE-2009-6971** | **2008** | **-66.54** | **143.99** | **AdL** | **786.9** |
| **Psi076** | **Psi076** | **CEAMARC** | **Psilaster charcoti** | **Christopher L. Mah** | **MNHN IE-2009-6971** | **2008** | **-66.54** | **143.99** | **AdL** | **786.9** |
| **Not336** | **Not336** | **CEAMARC** | **Notasterias armata** | **Christopher L. Mah** | **MNHN IE-2009-6981** | **2008** | **-66.54** | **144.97** | **AdL** | **440.7** |
| **ASTAN149-10** | **Psi072** | **CEAMARC** | **Psilaster charcoti** | **Christopher L. Mah** | **MNHN IE-2009-6982** | **2007** | **-66.32** | **143.65** | **AdL** | **570** |
| **Not281** | **Not281** | **CEAMARC** | **Notasterias bongraini** | **Christopher L. Mah** | **MNHN IE-2009-6985** | **2007** | **-66.32** | **143.65** | **AdL** | **570** |
| **ASTAN164-10** | **Bat052** | **CEAMARC** | **Bathybiaster loripes** | **Christopher L. Mah** | **MNHN IE-2009-7004** | **2007** | **-66.33** | **143.36** | **AdL** | **702.04** |
| **Bat058** | **Bat058** | **CEAMARC** | **Psilaster charcoti** | **Christopher L. Mah** | **MNHN IE-2009-7005** | **2007** | **-66.33** | **143.36** | **AdL** | **702.04** |
| **Bat059** | **Bat059** | **CEAMARC** | **Psilaster charcoti** | **Christopher L. Mah** | **MNHN IE-2009-7005** | **2007** | **-66.33** | **143.36** | **AdL** | **702.04** |
| **Bat060** | **Bat060** | **CEAMARC** | **Psilaster charcoti** | **Christopher L. Mah** | **MNHN IE-2009-7005** | **2007** | **-66.33** | **143.36** | **AdL** | **702.04** |
| **Bat061** | **Bat061** | **CEAMARC** | **Psilaster charcoti** | **Christopher L. Mah** | **MNHN IE-2009-7005** | **2007** | **-66.33** | **143.36** | **AdL** | **702.04** |
| **Bat062** | **Bat062** | **CEAMARC** | **Psilaster charcoti** | **Christopher L. Mah** | **MNHN IE-2009-7005** | **2007** | **-66.33** | **143.36** | **AdL** | **702.04** |
| **Psi077** | **Psi077** | **CEAMARC** | **Psilaster charcoti** | **Christopher L. Mah** | **MNHN IE-2009-7005** | **2007** | **-66.33** | **143.36** | **AdL** | **702.04** |
| **Psi078** | **Psi078** | **CEAMARC** | **Psilaster charcoti** | **Christopher L. Mah** | **MNHN IE-2009-7005** | **2007** | **-66.33** | **143.36** | **AdL** | **702.04** |
| **Psi079** | **Psi079** | **CEAMARC** | **Psilaster charcoti** | **Christopher L. Mah** | **MNHN IE-2009-7005** | **2007** | **-66.33** | **143.36** | **AdL** | **702.04** |
| **Lys258** | **Lys258** | **CEAMARC** | **Lysasterias cf. lactea** | **Christopher L. Mah** | **MNHN IE-2009-7014** | **2007** | **-66.55** | **142.64** | **AdL** | **138.7** |
| **Lys262** | **Lys262** | **CEAMARC** | **Lysasterias joffrei** | **Christopher L. Mah** | **MNHN IE-2009-7027** | **2007** | **-66.00** | **142.95** | **AdL** | **464.8** |
| **Lys263** | **Lys263** | **CEAMARC** | **Lysasterias joffrei** | **Christopher L. Mah** | **MNHN IE-2009-7027** | **2007** | **-66.00** | **142.95** | **AdL** | **464.8** |
| **Psi062** | **Psi062** | **CEAMARC** | **Bathybiaster loripes** | **Christopher L. Mah** | **MNHN IE-2009-7052** | **2008** |  |  | **AdL** |  |
| **Psi063** | **Psi063** | **CEAMARC** | **Bathybiaster loripes** | **Christopher L. Mah** | **MNHN IE-2009-7053** | **2008** |  |  | **AdL** |  |
| **Bat046** | **Bat046** | **CEAMARC** | **Bathybiaster loripes** | **Christopher L. Mah** | **MNHN IE-2009-7058** | **2008** | **-66.32** | **143.30** | **AdL** | **692.6** |
| **Bat063** | **Bat063** | **CEAMARC** | **Psilaster charcoti** | **Christopher L. Mah** | **MNHN IE-2009-7059** | **2008** | **-66.32** | **143.30** | **AdL** | **692.6** |
| **Bat064** | **Bat064** | **CEAMARC** | **Psilaster charcoti** | **Christopher L. Mah** | **MNHN IE-2009-7059** | **2008** | **-66.32** | **143.30** | **AdL** | **692.6** |
| **Psi080** | **Psi080** | **CEAMARC** | **Psilaster charcoti** | **Christopher L. Mah** | **MNHN IE-2009-7059** | **2008** | **-66.32** | **143.30** | **AdL** | **692.6** |
| **Lys231** | **Lys231** | **CEAMARC** | **Lysasterias cf. lactea** | **Christopher L. Mah** | **MNHN IE-2009-7065** | **2008** | **-65.82** | **142.96** | **AdL** | **774.9** |
| **Psi046** | **Psi046** | **CEAMARC** | **Psilaster charcoti** | **Christopher L. Mah** | **MNHN IE-2009-7076** | **2008** | **-66.32** | **144.31** | **AdL** | **451.9** |
| **Not345** | **Not345** | **CEAMARC** | **Notasterias bongraini** | **Christopher L. Mah** | **MNHN IE-2009-7081** | **2008** | **-66.00** | **142.01** | **AdL** | **244.57** |
| **Bat047** | **Bat047** | **CEAMARC** | **Bathybiaster loripes** | **Christopher L. Mah** | **MNHN IE-2009-7087** | **2008** | **-66.32** | **144.31** | **AdL** | **451.9** |
| **Not320** | **Not320** | **CEAMARC** | **Notasterias armata** | **Christopher L. Mah** | **MNHN IE-2009-7091** | **2008** | **-66.32** | **144.64** | **AdL** | **420.3** |
| **Bat040** | **Bat040** | **CEAMARC** | **Bathybiaster loripes** | **Christopher L. Mah** | **MNHN IE-2009-7103** | **2008** | **-66.74** | **144.31** | **AdL** | **903.6** |
| **Bat041** | **Bat041** | **CEAMARC** | **Bathybiaster loripes** | **Christopher L. Mah** | **MNHN IE-2009-7104** | **2008** | **-66.74** | **144.31** | **AdL** | **903.6** |
| **Bat039** | **Bat039** | **CEAMARC** | **Bathybiaster loripes** | **Christopher L. Mah** | **MNHN IE-2009-7110** | **2008** | **-66.52** | **140.00** | **AdL** | **175.9** |
| **Lys244** | **Lys244** | **CEAMARC** | **Lysasterias joffrei** | **Christopher L. Mah** | **MNHN IE-2009-7111** | **2008** | **-66.52** | **140.00** | **AdL** | **175.9** |
| **Not273** | **Not273** | **CEAMARC** | **Notasterias bongraini** | **Christopher L. Mah** | **MNHN IE-2009-7112** | **2008** | **-66.32** | **143.98** | **AdL** | **504.3** |
| **Lys236** | **Lys236** | **CEAMARC** | **Lysasterias cf. lactea** | **Christopher L. Mah** | **MNHN IE-2009-7120** | **2008** | **-66.32** | **143.98** | **AdL** | **504.3** |
| **Lys230** | **Lys230** | **CEAMARC** | **Lysasterias joffrei** | **Christopher L. Mah** | **MNHN IE-2009-7122** | **2008** | **-66.32** | **143.98** | **AdL** | **504.3** |
| **ASTAN255-10** | **Bat021** | **CEAMARC** | **Bathybiaster loripes** | **Christopher L. Mah** | **MNHN IE-2009-7124** | **2008** | **-66.32** | **143.98** | **AdL** | **504.3** |
| **Bat022** | **Bat022** | **CEAMARC** | **Bathybiaster loripes** | **Christopher L. Mah** | **MNHN IE-2009-7125** | **2008** | **-66.32** | **143.98** | **AdL** | **504.3** |
| **Not323** | **Not323** | **CEAMARC** | **Notasterias armata** | **Christopher L. Mah** | **MNHN IE-2009-7138** | **2008** | **-65.87** | **143.00** | **AdL** | **430.2** |
| **Not292** | **Not292** | **CEAMARC** | **Notasterias armata** | **Christopher L. Mah** | **MNHN IE-2009-7149** | **2008** | **-66.14** | **143.30** | **AdL** | **534.1** |
| **Bat044** | **Bat044** | **CEAMARC** | **Bathybiaster loripes** | **Christopher L. Mah** | **MNHN IE-2009-7157** | **2008** | **-66.54** | **145.29** | **AdL** | **403.5** |
| **Lys250** | **Lys250** | **CEAMARC** | **Lysasterias cf. lactea** | **Christopher L. Mah** | **MNHN IE-2009-7169** | **2008** | **-66.32** | **143.63** | **AdL** | **566.4** |
| **Not331** | **Not331** | **CEAMARC** | **Notasterias armata** | **Christopher L. Mah** | **MNHN IE-2009-7170** | **2008** | **-66.32** | **143.63** | **AdL** | **566.4** |
| **Dip185** | **Dip185** | **CEAMARC** | **Diplasterias sp** | **Christopher L. Mah** | **MNHN IE-2009-7179** | **2008** | **-66.34** | **141.27** | **AdL** | **206.7** |
| **Psi143** | **Psi143** | **CEAMARC** | **Psilaster charcoti** | **Christopher L. Mah** | **MNHN IE-2009-7189** | **2008** | **-66.34** | **141.27** | **AdL** | **206.7** |
| **Lys232** | **Lys232** | **CEAMARC** | **Lysasterias cf. lactea** | **Christopher L. Mah** | **MNHN IE-2009-7207** | **2008** | **-66.00** | **141.35** | **AdL** | **232.6** |
| **Psi142** | **Psi142** | **CEAMARC** | **Psilaster charcoti** | **Christopher L. Mah** | **MNHN IE-2009-7247** | **2008** | **-66.56** | **141.26** | **AdL** | **170.2** |
| **Lys238** | **Lys238** | **CEAMARC** | **Lysasterias joffrei** | **Christopher L. Mah** | **MNHN IE-2009-7266** | **2008** | **-66.32** | **144.31** | **AdL** | **451.9** |
| **ASTAN413-10** | **Bat013** | **CEAMARC** | **Bathybiaster loripes** | **Christopher L. Mah** | **MNHN IE-2009-7275** | **2008** | **-66.32** | **144.31** | **AdL** | **451.9** |
| **Bat016** | **Bat016** | **CEAMARC** | **Bathybiaster loripes** | **Christopher L. Mah** | **MNHN IE-2009-7276** | **2008** | **-66.32** | **144.31** | **AdL** | **451.9** |
| **Not285** | **Not285** | **CEAMARC** | **Notasterias bongraini** | **Christopher L. Mah** | **MNHN IE-2009-7282** | **2008** | **-66.34** | **140.45** | **AdL** | **443.7** |
| **Not286** | **Not286** | **CEAMARC** | **Notasterias bongraini** | **Christopher L. Mah** | **MNHN IE-2009-7283** | **2008** | **-66.34** | **140.45** | **AdL** | **443.7** |
| **Not343** | **Not343** | **CEAMARC** | **Notasterias armata** | **Christopher L. Mah** | **MNHN IE-2009-7287** | **2008** | **-65.51** | **139.36** | **AdL** | **398.4** |
| **Lys229** | **Lys229** | **CEAMARC** | **Lysasterias joffrei** | **Christopher L. Mah** | **MNHN IE-2009-7289** | **2008** | **-65.51** | **139.36** | **AdL** | **398.4** |
| **ASTAN426-10** | **Bat023** | **CEAMARC** | **Bathybiaster loripes** | **Christopher L. Mah** | **MNHN IE-2009-7292** | **2008** | **-65.51** | **139.36** | **AdL** | **398** |
| **Psi140** | **Psi140** | **CEAMARC** | **Psilaster charcoti** | **Christopher L. Mah** | **MNHN IE-2009-7296** | **2008** | **-65.71** | **140.60** | **AdL** | **423.9** |
| **Not289** | **Not289** | **CEAMARC** | **Notasterias bongraini** | **Christopher L. Mah** | **MNHN IE-2009-7308** | **2008** | **-65.71** | **140.60** | **AdL** | **423.9** |
| **Lys248** | **Lys248** | **CEAMARC** | **Lysasterias cf. lactea** | **Christopher L. Mah** | **MNHN IE-2009-7309** | **2008** | **-65.71** | **140.60** | **AdL** | **423.9** |
| **Not264bis** | **Not264bis** | **CEAMARC** | **Notasterias sp** | **Christopher L. Mah** | **MNHN IE-2009-7332** | **2008** | **-65.46** | **139.307988** | **AdL** | **815** |
| **Not310** | **Not310** | **CEAMARC** | **Notasterias armata** | **Christopher L. Mah** | **MNHN IE-2009-7337** | **2008** | **-66.17** | **139.93** | **AdL** | **149.9** |
| **Not311** | **Not311** | **CEAMARC** | **Notasterias armata** | **Christopher L. Mah** | **MNHN IE-2009-7338** | **2008** | **-66.17** | **139.93** | **AdL** | **149.9** |
| **Psi144** | **Psi144** | **CEAMARC** | **Bathybiaster loripes** | **Christopher L. Mah** | **MNHN IE-2009-7349** | **2008** | **-65.99** | **139.31** | **AdL** | **472** |
| **Not333** | **Not333** | **CEAMARC** | **Notasterias stolophora** | **Christopher L. Mah** | **MNHN IE-2009-7352** | **2008** | **-65.99** | **139.31** | **AdL** | **472.4** |
| **Not347** | **Not347** | **CEAMARC** | **Notasterias bongraini** | **Christopher L. Mah** | **MNHN IE-2009-7355** | **2008** | **-65.99** | **139.99** | **AdL** | **192.1** |
| **Not283** | **Not283** | **CEAMARC** | **Notasterias armata** | **Christopher L. Mah** | **MNHN IE-2009-7364** | **2008** | **-65.70** | **140.49** | **AdL** | **820.9** |
| **Not284** | **Not284** | **CEAMARC** | **Notasterias armata** | **Christopher L. Mah** | **MNHN IE-2009-7365** | **2008** | **-65.70** | **140.49** | **AdL** | **820.9** |
| **Lys252** | **Lys252** | **CEAMARC** | **Lysasterias cf. lactea** | **Christopher L. Mah** | **MNHN IE-2009-7374** | **2008** | **-65.91** | **143.97** | **AdL** | **370.4** |
| **Lys253** | **Lys253** | **CEAMARC** | **Lysasterias cf. lactea** | **Christopher L. Mah** | **MNHN IE-2009-7375** | **2008** | **-65.91** | **143.97** | **AdL** | **370.4** |
| **Lys237** | **Lys237** | **CEAMARC** | **Lysasterias cf. lactea** | **Christopher L. Mah** | **MNHN IE-2009-7378** | **2008** | **-65.91** | **143.97** | **AdL** | **370.4** |
| **ASTAN508-10** | **Psi186** | **CEAMARC** | **Psilaster charcoti** | **Christopher L. Mah** | **MNHN IE-2009-7403** | **2008** | **-66.00** | **142.31** | **AdL** | **233.52** |
| **Lys251** | **Lys251** | **CEAMARC** | **Lysasterias cf. lactea** | **Christopher L. Mah** | **MNHN IE-2009-7418** | **2008** | **-66.52** | **140.00** | **AdL** | **175.9** |
| **Not305** | **Not305** | **CEAMARC** | **Notasterias brachiata** | **Christopher L. Mah** | **MNHN IE-2009-8012** | **2008** | **-66.34** | **140.03** | **AdL** | **509.9** |
| **Bat037** | **Bat037** | **CEAMARC** | **Bathybiaster loripes** | **Christopher L. Mah** | **MNHN IE-2009-8017** | **2008** | **-66.41** | **140.51** | **AdL** | **971.5** |
| **ASTAN546-10** | **Psi047** | **CEAMARC** | **Psilaster charcoti** | **Christopher L. Mah** | **MNHN IE-2009-8018** | **2008** | **-66.41** | **140.51** | **AdL** | **971.5** |
| **Psi048** | **Psi048** | **CEAMARC** | **Psilaster charcoti** | **Christopher L. Mah** | **MNHN IE-2009-8019** | **2008** | **-66.41** | **140.51** | **AdL** | **971.5** |
| **Not301** | **Not301** | **CEAMARC** | **Notasterias brachiata** | **Christopher L. Mah** | **MNHN IE-2009-8030** | **2008** | **-66.33** | **140.65** | **AdL** | **164.9** |
| **Not314** | **Not314** | **CEAMARC** | **Notasterias armata** | **Christopher L. Mah** | **MNHN IE-2009-8039** | **2008** | **-66.33** | **140.65** | **AdL** | **164.9** |
| **Psi141** | **Psi141** | **CEAMARC** | **Psilaster charcoti** | **Christopher L. Mah** | **MNHN IE-2009-8040** | **2008** | **-65.99** | **139.31** | **AdL** | **472** |
| **Psi064** | **Psi064** | **CEAMARC** | **Bathybiaster loripes** | **Christopher L. Mah** | **MNHN IE-2009-9008** | **2008** |  |  | **AdL** |  |
| **Psi065** | **Psi065** | **CEAMARC** | **Bathybiaster loripes** | **Christopher L. Mah** | **MNHN IE-2009-9009** | **2008** |  |  | **AdL** |  |
| **Psi066** | **Psi066** | **CEAMARC** | **Bathybiaster loripes** | **Christopher L. Mah** | **MNHN IE-2009-9010** | **2008** |  |  | **AdL** |  |
| **Bat014** | **Bat014** | **CEAMARC** | **Bathybiaster loripes** | **Christopher L. Mah** | **MNHN IE-2009-9011** | **2008** | **-66.32** | **144.31** | **AdL** | **451.9** |
| **Bat015** | **Bat015** | **CEAMARC** | **Bathybiaster loripes** | **Christopher L. Mah** | **MNHN IE-2009-9012** | **2008** | **-66.32** | **144.31** | **AdL** | **451.9** |
| **Bat026** | **Bat026** | **CEAMARC** | **Bathybiaster loripes** | **Christopher L. Mah** | **MNHN IE-2009-9013** | **2008** | **-66.41** | **140.51** | **AdL** | **971.5** |
| **Bat025** | **Bat025** | **CEAMARC** | **Bathybiaster loripes** | **Christopher L. Mah** | **MNHN IE-2009-9014** | **2008** | **-66.41** | **140.51** | **AdL** | **971.5** |
| **Bat027** | **Bat027** | **CEAMARC** | **Bathybiaster loripes** | **Christopher L. Mah** | **MNHN IE-2009-9015** | **2008** | **-66.41** | **140.51** | **AdL** | **971.5** |
| **Bat028** | **Bat028** | **CEAMARC** | **Bathybiaster loripes** | **Christopher L. Mah** | **MNHN IE-2009-9016** | **2008** | **-66.41** | **140.51** | **AdL** | **971.5** |
| **Bat043** | **Bat043** | **CEAMARC** | **Bathybiaster loripes** | **Christopher L. Mah** | **MNHN IE-2009-9018** | **2008** |  |  | **AdL** |  |
| **Bat038** | **Bat038** | **CEAMARC** | **Bathybiaster loripes** | **Christopher L. Mah** | **MNHN IE-2009-9019** | **2008** |  |  | **AdL** |  |
| **SOA261-12** | **Bat205** | **POKER II** | **Bathybiaster loripes** | **Christopher L. Mah** | **MNHN IE-2011-1009** | **2011** | **-50.38** | **71.40** | **Ker** | **626** |
| **Dip361** | **Dip361** | **POKER II** | **Diplasterias meridionalis** | **Christopher L. Mah** | **MNHN IE-2011-1106** | **2011** | **-48.94** | **70.77** | **Ker** | **80** |
| **SOA332-12** | **Bat203** | **POKER II** | **Bathybiaster loripes** | **Christopher L. Mah** | **MNHN IE-2011-1172** | **2011** | **-47.81** | **67.47** | **Ker** | **394** |
| **SOA335-12** | **Bat196** | **POKER II** | **Bathybiaster loripes** | **Christopher L. Mah** | **MNHN IE-2011-1178** | **2011** | **-47.65** | **67.91** | **Ker** | **250** |
| **SOA337-12** | **Bat194** | **POKER II** | **Bathybiaster loripes** | **Christopher L. Mah** | **MNHN IE-2011-1183** | **2011** | **-47.72** | **68.68** | **Ker** | **217** |
| **SOA345-12** | **Dip366** | **POKER II** | **Diplasterias meridionalis** | **Christopher L. Mah** | **MNHN IE-2011-1195** | **2011** | **-48.35** | **67.68** | **Ker** | **211** |
| **Dip360** | **Dip360** | **POKER II** | **Diplasterias meridionalis** | **Christopher L. Mah** | **MNHN IE-2011-1208** | **2011** | **-49.10** | **70.89** | **Ker** | **110** |
| **SOA455-12** | **Bat161** | **POKER II** | **Bathybiaster loripes** | **Christopher L. Mah** | **MNHN IE-2011-124** | **2011** | **-48.13** | **69.11** | **Ker** | **171** |
| **SOA456-12** | **Bat182** | **POKER II** | **Bathybiaster loripes** | **Christopher L. Mah** | **MNHN IE-2011-125** | **2011** | **-48.13** | **69.11** | **Ker** | **171** |
| **SOA457-12** | **Dip330** | **POKER II** | **Diplasterias meridionalis** | **Christopher L. Mah** | **MNHN IE-2011-126** | **2011** | **-48.13** | **69.11** | **Ker** | **171** |
| **SOA384-12** | **Bat200** | **POKER II** | **Bathybiaster loripes** | **Christopher L. Mah** | **MNHN IE-2011-1277** | **2011** | **-48.84** | **70.11** | **Ker** | **103** |
| **SOA387-12** | **Bat197** | **POKER II** | **Bathybiaster loripes** | **Christopher L. Mah** | **MNHN IE-2011-1280** | **2011** | **-48.84** | **70.11** | **Ker** | **103** |
| **SOA391-12** | **Dip372** | **POKER II** | **Diplasterias meridionalis** | **Christopher L. Mah** | **MNHN IE-2011-1308** | **2011** | **-47.91** | **67.13** | **Ker** | **494** |
| **SOA394-12** | **Bat201** | **POKER II** | **Bathybiaster loripes** | **Christopher L. Mah** | **MNHN IE-2011-1312** | **2011** | **-47.91** | **67.13** | **Ker** | **494** |
| **SOA395-12** | **Bat273** | **POKER II** | **Bathybiaster loripes** | **Christopher L. Mah** | **MNHN IE-2011-1316** | **2011** | **-48.71** | **70.96** | **Ker** | **191** |
| **SOA397-12** | **Dip358** | **POKER II** | **Diplasterias meridionalis** | **Christopher L. Mah** | **MNHN IE-2011-1318** | **2011** | **-48.71** | **70.96** | **Ker** | **191** |
| **SOA400-12** | **Bat207** | **POKER II** | **Bathybiaster loripes** | **Christopher L. Mah** | **MNHN IE-2011-1324** | **2011** | **-47.91** | **67.13** | **Ker** | **494** |
| **SOA497-12** | **Dip427** | **POKER II** | **Diplasterias meridionalis** | **Christopher L. Mah** | **MNHN IE-2011-1332** | **2011** | **-49.91** | **68.38** | **Ker** | **172** |
| **SOA468-12** | **Bat190** | **POKER II** | **Bathybiaster loripes** | **Christopher L. Mah** | **MNHN IE-2011-145** | **2011** | **-49.45** | **72.00** | **Ker** | **746** |
| **SOA470-12** | **Bat183** | **POKER II** | **Bathybiaster loripes** | **Christopher L. Mah** | **MNHN IE-2011-154** | **2011** | **-50.46** | **72.60** | **Ker** | **544** |
| **SOA471-12** | **Bat179** | **POKER II** | **Bathybiaster loripes** | **Christopher L. Mah** | **MNHN IE-2011-163** | **2011** | **-50.90** | **72.34** | **Ker** | **516** |
| **SOA473-12** | **Bat172** | **POKER II** | **Bathybiaster loripes** | **Christopher L. Mah** | **MNHN IE-2011-172** | **2011** | **-47.80** | **71.24** | **Ker** | **216** |
| **SOA474-12** | **Bat174** | **POKER II** | **Bathybiaster loripes** | **Christopher L. Mah** | **MNHN IE-2011-173** | **2011** | **-47.80** | **71.24** | **Ker** | **216** |
| **SOA415-12** | **Dip327** | **POKER II** | **Diplasterias meridionalis** | **Christopher L. Mah** | **MNHN IE-2011-21** | **2011** | **-47.82** | **70.03** | **Ker** | **161** |
| **SOA482-12** | **Bat185** | **POKER II** | **Bathybiaster loripes** | **Christopher L. Mah** | **MNHN IE-2011-213** | **2011** | **-50.27** | **72.62** | **Ker** | **527** |
| **SOA491-12** | **Dip347** | **POKER II** | **Diplasterias meridionalis** | **Christopher L. Mah** | **MNHN IE-2011-224** | **2011** | **-49.61** | **70.63** | **Ker** | **124** |
| **SOA502-12** | **Dip317** | **POKER II** | **Diplasterias meridionalis** | **Christopher L. Mah** | **MNHN IE-2011-254** | **2011** | **-50.33** | **69.32** | **Ker** | **220** |
| **SOA512-12** | **Dip325a** | **POKER II** | **Diplasterias meridionalis** | **Christopher L. Mah** | **MNHN IE-2011-295** | **2011** | **-49.46** | **70.82** | **Ker** | **119** |
| **SOA513-12** | **Bat274** | **POKER II** | **Bathybiaster loripes** | **Christopher L. Mah** | **MNHN IE-2011-310** | **2011** | **-49.96** | **70.60** | **Ker** | **279** |
| **SOA521-12** | **Bat178** | **POKER II** | **Bathybiaster loripes** | **Christopher L. Mah** | **MNHN IE-2011-327** | **2011** | **-49.80** | **67.86** | **Ker** | **218** |
| **SOA417-12** | **Bat163** | **POKER II** | **Bathybiaster loripes** | **Christopher L. Mah** | **MNHN IE-2011-34** | **2011** | **-48.56** | **70.54** | **Ker** | **113** |
| **SOA418-12** | **Bat165** | **POKER II** | **Bathybiaster loripes** | **Christopher L. Mah** | **MNHN IE-2011-35** | **2011** | **-48.56** | **70.54** | **Ker** | **113** |
| **SOA531-12** | **Bat181** | **POKER II** | **Bathybiaster loripes** | **Christopher L. Mah** | **MNHN IE-2011-352** | **2011** | **-48.23** | **71.07** | **Ker** | **347** |
| **SOA535-12** | **Bat177** | **POKER II** | **Bathybiaster loripes** | **Christopher L. Mah** | **MNHN IE-2011-357** | **2011** | **-47.67** | **72.05** | **Ker** | **601** |
| **SOA420-12** | **Dip328** | **POKER II** | **Diplasterias meridionalis** | **Christopher L. Mah** | **MNHN IE-2011-37** | **2011** | **-48.56** | **70.54** | **Ker** | **113** |
| **SOA549-12** | **Dip428** | **POKER II** | **Diplasterias meridionalis** | **Christopher L. Mah** | **MNHN IE-2011-383** | **2011** | **-47.76** | **72.09** | **Ker** | **562** |
| **SOA548-12** | **Bat160** | **POKER II** | **Bathybiaster loripes** | **Christopher L. Mah** | **MNHN IE-2011-384** | **2011** | **-47.76** | **72.09** | **Ker** | **562** |
| **SOA552-12** | **Bat159** | **POKER II** | **Bathybiaster loripes** | **Christopher L. Mah** | **MNHN IE-2011-390** | **2011** | **-47.74** | **71.07** | **Ker** | **192** |
| **SOA553-12** | **Dip349** | **POKER II** | **Diplasterias meridionalis** | **Christopher L. Mah** | **MNHN IE-2011-393** | **2011** | **-47.52** | **71.01** | **Ker** | **199** |
| **SOA563-12** | **Dip341** | **POKER II** | **Diplasterias meridionalis** | **Christopher L. Mah** | **MNHN IE-2011-412** | **2011** | **-47.61** | **71.85** | **Ker** | **462** |
| **SOA566-12** | **Bat187** | **POKER II** | **Bathybiaster loripes** | **Christopher L. Mah** | **MNHN IE-2011-418** | **2011** | **-47.43** | **70.84** | **Ker** | **198** |
| **SOA002-12** | **Dip320** | **POKER II** | **Diplasterias meridionalis** | **Christopher L. Mah** | **MNHN IE-2011-437** | **2008** | **-47.19** | **66.12** | **Ker** | **650** |
| **SOA010-12** | **Bat175** | **POKER II** | **Bathybiaster loripes** | **Christopher L. Mah** | **MNHN IE-2011-453** | **2011** | **-48.14** | **70.56** | **Ker** | **142** |
| **SOA012-12** | **Bat189** | **POKER II** | **Bathybiaster loripes** | **Christopher L. Mah** | **MNHN IE-2011-459** | **2011** | **-47.42** | **66.80** | **Ker** | **266** |
| **SOA025-12** | **Bat166** | **POKER II** | **Bathybiaster loripes** | **Christopher L. Mah** | **MNHN IE-2011-483** | **2011** | **-46.96** | **70.45** | **Ker** | **524** |
| **SOA027-12** | **Dip351** | **POKER II** | **Diplasterias meridionalis** | **Christopher L. Mah** | **MNHN IE-2011-485** | **2011** | **-47.03** | **70.47** | **Ker** | **414** |
| **SOA028-12** | **Bat192** | **POKER II** | **Bathybiaster loripes** | **Christopher L. Mah** | **MNHN IE-2011-486** | **2011** | **-47.03** | **70.47** | **Ker** | **414** |
| **SOA030-12** | **Dip331** | **POKER II** | **Diplasterias meridionalis** | **Christopher L. Mah** | **MNHN IE-2011-488** | **2011** | **-48.44** | **70.60** | **Ker** | **120** |
| **SOA042-12** | **Bat110** | **POKER II** | **Bathybiaster loripes** | **Christopher L. Mah** | **MNHN IE-2011-505** | **2011** | **-47.83** | **68.39** | **Ker** | **223** |
| **SOA045-12** | **Dip238** | **POKER II** | **Diplasterias meridionalis** | **Christopher L. Mah** | **MNHN IE-2011-508** | **2011** | **-47.83** | **68.39** | **Ker** | **223** |
| **Dip231** | **Dip231** | **POKER II** | **Diplasterias meridionalis** | **Christopher L. Mah** | **MNHN IE-2011-509** | **2011** | **-47.83** | **68.39** | **Ker** | **223** |
| **SOA429-12** | **Dip336** | **POKER II** | **Diplasterias meridionalis** | **Christopher L. Mah** | **MNHN IE-2011-51** | **2011** | **-48.98** | **69.97** | **Ker** | **98** |
| **SOA049-12** | **Dip235** | **POKER II** | **Diplasterias meridionalis** | **Christopher L. Mah** | **MNHN IE-2011-516** | **2011** | **-47.59** | **68.61** | **Ker** | **217** |
| **SOA052-12** | **Bat122** | **POKER II** | **Bathybiaster loripes** | **Christopher L. Mah** | **MNHN IE-2011-521** | **2011** | **-47.59** | **68.61** | **Ker** | **217** |
| **SOA055-12** | **Bat115** | **POKER II** | **Bathybiaster loripes** | **Christopher L. Mah** | **MNHN IE-2011-528** | **2011** | **-46.39** | **67.89** | **Ker** | **831** |
| **SOA059-12** | **Bat111** | **POKER II** | **Bathybiaster loripes** | **Christopher L. Mah** | **MNHN IE-2011-534** | **2011** | **-46.97** | **70.66** | **Ker** | **943** |
| **SOA062-12** | **Dip229** | **POKER II** | **Diplasterias meridionalis** | **Christopher L. Mah** | **MNHN IE-2011-541** | **2011** | **-47.24** | **70.74** | **Ker** | **310** |
| **SOA063-12** | **Bat116** | **POKER II** | **Bathybiaster loripes** | **Christopher L. Mah** | **MNHN IE-2011-542** | **2011** | **-47.24** | **70.74** | **Ker** | **310** |
| **SOA066-12** | **Bat112** | **POKER II** | **Bathybiaster loripes** | **Christopher L. Mah** | **MNHN IE-2011-546** | **2011** | **-47.18** | **70.62** | **Ker** | **228** |
| **SOA069-12** | **Bat114** | **POKER II** | **Bathybiaster loripes** | **Christopher L. Mah** | **MNHN IE-2011-551** | **2011** | **-47.60** | **70.27** | **Ker** | **161** |
| **SOA070-12** | **Bat118** | **POKER II** | **Bathybiaster loripes** | **Christopher L. Mah** | **MNHN IE-2011-552** | **2011** | **-47.60** | **70.27** | **Ker** | **161** |
| **SOA077-12** | **Bat109** | **POKER II** | **Bathybiaster loripes** | **Christopher L. Mah** | **MNHN IE-2011-561** | **2011** | **-47.24** | **70.65** | **Ker** | **190** |
| **SOA078-12** | **Dip233** | **POKER II** | **Diplasterias meridionalis** | **Christopher L. Mah** | **MNHN IE-2011-562** | **2011** | **-47.24** | **70.65** | **Ker** | **190** |
| **Dip230** | **Dip230** | **POKER II** | **Diplasterias meridionalis** | **Christopher L. Mah** | **MNHN IE-2011-563** | **2011** | **-47.24** | **70.65** | **Ker** | **190** |
| **SOA079-12** | **Dip236** | **POKER II** | **Diplasterias meridionalis** | **Christopher L. Mah** | **MNHN IE-2011-566** | **2011** | **-47.65** | **70.48** | **Ker** | **160** |
| **SOA080-12** | **Bat117** | **POKER II** | **Bathybiaster loripes** | **Christopher L. Mah** | **MNHN IE-2011-567** | **2011** | **-47.65** | **70.48** | **Ker** | **160** |
| **SOA081-12** | **Bat275** | **POKER II** | **Bathybiaster loripes** | **Christopher L. Mah** | **MNHN IE-2011-568** | **2011** | **-47.65** | **70.48** | **Ker** | **160** |
| **SOA082-12** | **Bat121** | **POKER II** | **Bathybiaster loripes** | **Christopher L. Mah** | **MNHN IE-2011-569** | **2011** | **-47.69** | **70.17** | **Ker** | **160** |
| **SOA083-12** | **Bat113** | **POKER II** | **Bathybiaster loripes** | **Christopher L. Mah** | **MNHN IE-2011-570** | **2011** | **-47.69** | **70.17** | **Ker** | **160** |
| **SOA086-12** | **Bat119** | **POKER II** | **Bathybiaster loripes** | **Christopher L. Mah** | **MNHN IE-2011-580** | **2011** | **-51.37** | **70.25** | **Ker** | **760** |
| **SOA405-12** | **Dip334** | **POKER II** | **Diplasterias meridionalis** | **Christopher L. Mah** | **MNHN IE-2011-6** | **2011** | **-48.24** | **68.56** | **Ker** | **165** |
| **SOA431-12** | **Dip333** | **POKER II** | **Diplasterias meridionalis** | **Christopher L. Mah** | **MNHN IE-2011-62** | **2011** | **-48.08** | **70.48** | **Ker** | **145** |
| **SOA102-12** | **Bat130** | **POKER II** | **Bathybiaster loripes** | **Christopher L. Mah** | **MNHN IE-2011-631** | **2011** | **-50.45** | **71.61** | **Ker** | **596** |
| **SOA104-12** | **Bat141** | **POKER II** | **Bathybiaster loripes** | **Christopher L. Mah** | **MNHN IE-2011-645** | **2011** | **-47.27** | **67.15** | **Ker** | **519** |
| **SOA106-12** | **Bat142** | **POKER II** | **Bathybiaster loripes** | **Christopher L. Mah** | **MNHN IE-2011-655** | **2011** | **-47.29** | **67.67** | **Ker** | **347** |
| **SOA107-12** | **Bat131** | **POKER II** | **Bathybiaster loripes** | **Christopher L. Mah** | **MNHN IE-2011-656** | **2011** | **-47.29** | **67.67** | **Ker** | **347** |
| **SOA108-12** | **Bat129** | **POKER II** | **Bathybiaster loripes** | **Christopher L. Mah** | **MNHN IE-2011-657** | **2011** | **-47.29** | **67.67** | **Ker** | **347** |
| **SOA109-12** | **Bat144** | **POKER II** | **Bathybiaster loripes** | **Christopher L. Mah** | **MNHN IE-2011-658** | **2011** | **-47.29** | **67.67** | **Ker** | **347** |
| **SOA110-12** | **Bat123** | **POKER II** | **Bathybiaster loripes** | **Christopher L. Mah** | **MNHN IE-2011-659** | **2011** | **-47.29** | **67.67** | **Ker** | **347** |
| **SOA433-12** | **Bat173** | **POKER II** | **Bathybiaster loripes** | **Christopher L. Mah** | **MNHN IE-2011-66** | **2011** | **-48.08** | **70.48** | **Ker** | **145** |
| **SOA111-12** | **Bat143** | **POKER II** | **Bathybiaster loripes** | **Christopher L. Mah** | **MNHN IE-2011-660** | **2011** | **-47.29** | **67.67** | **Ker** | **347** |
| **SOA122-12** | **Bat136** | **POKER II** | **Bathybiaster loripes** | **Christopher L. Mah** | **MNHN IE-2011-673** | **2011** | **-48.07** | **67.60** | **Ker** | **355** |
| **SOA127-12** | **Bat134** | **POKER II** | **Bathybiaster loripes** | **Christopher L. Mah** | **MNHN IE-2011-683** | **2011** | **-49.92** | **71.22** | **Ker** | **615** |
| **SOA131-12** | **Dip429** | **POKER II** | **Diplasterias meridionalis** | **Christopher L. Mah** | **MNHN IE-2011-691** | **2011** | **-47.27** | **67.83** | **Ker** | **305** |
| **SOA132-12** | **Bat140** | **POKER II** | **Bathybiaster loripes** | **Christopher L. Mah** | **MNHN IE-2011-692** | **2011** | **-47.27** | **67.83** | **Ker** | **305** |
| **SOA133-12** | **Bat127** | **POKER II** | **Bathybiaster loripes** | **Christopher L. Mah** | **MNHN IE-2011-693** | **2011** | **-47.27** | **67.83** | **Ker** | **305** |
| **SOA406-12** | **Bat186** | **POKER II** | **Bathybiaster loripes** | **Christopher L. Mah** | **MNHN IE-2011-7** | **2011** | **-48.24** | **68.56** | **Ker** | **165** |
| **Dip348** | **Dip348** | **POKER II** | **Diplasterias meridionalis** | **Christopher L. Mah** | **MNHN IE-2011-72** | **2011** | **-48.05** | **69.16** | **Ker** | **172** |
| **SOA146-12** | **Bat135** | **POKER II** | **Bathybiaster loripes** | **Christopher L. Mah** | **MNHN IE-2011-740** | **2011** | **-47.33** | **66.95** | **Ker** | **518** |
| **SOA147-12** | **Bat128** | **POKER II** | **Bathybiaster loripes** | **Christopher L. Mah** | **MNHN IE-2011-741** | **2011** | **-47.33** | **66.95** | **Ker** | **518** |
| **SOA153-12** | **Bat137** | **POKER II** | **Bathybiaster loripes** | **Christopher L. Mah** | **MNHN IE-2011-760** | **2011** | **-47.59** | **68.61** | **Ker** | **217** |
| **SOA157-12** | **Bat126** | **POKER II** | **Bathybiaster loripes** | **Christopher L. Mah** | **MNHN IE-2011-765** | **2011** | **-47.31** | **68.33** | **Ker** | **208** |
| **SOA159-12** | **Dip244** | **POKER II** | **Diplasterias meridionalis** | **Christopher L. Mah** | **MNHN IE-2011-767** | **2011** | **-47.31** | **68.33** | **Ker** | **208** |
| **SOA161-12** | **Bat125** | **POKER II** | **Bathybiaster loripes** | **Christopher L. Mah** | **MNHN IE-2011-770** | **2011** | **-49.83** | **64.65** | **Ker** | **322** |
| **SOA165-12** | **Bat139** | **POKER II** | **Bathybiaster loripes** | **Christopher L. Mah** | **MNHN IE-2011-774** | **2011** | **-46.49** | **67.80** | **Ker** | **739** |
| **SOA166-12** | **Bat132** | **POKER II** | **Bathybiaster loripes** | **Christopher L. Mah** | **MNHN IE-2011-775** | **2011** | **-46.49** | **67.80** | **Ker** | **739** |
| **SOA169-12** | **Bat124** | **POKER II** | **Bathybiaster loripes** | **Christopher L. Mah** | **MNHN IE-2011-783** | **2011** | **-47.03** | **67.96** | **Ker** | **427** |
| **SOA181-12** | **Dip250** | **POKER II** | **Diplasterias meridionalis** | **Christopher L. Mah** | **MNHN IE-2011-807** | **2011** | **-47.44** | **69.21** | **Ker** | **313** |
| **SOA187-12** | **Dip246** | **POKER II** | **Diplasterias meridionalis** | **Christopher L. Mah** | **MNHN IE-2011-816** | **2011** | **-47.75** | **69.62** | **Ker** | **170** |
| **SOA205-12** | **Bat149** | **POKER II** | **Bathybiaster loripes** | **Christopher L. Mah** | **MNHN IE-2011-853** | **2011** | **-47.53** | **69.65** | **Ker** | **182** |
| **SOA218-12** | **Bat146** | **POKER II** | **Bathybiaster loripes** | **Christopher L. Mah** | **MNHN IE-2011-878** | **2011** | **-49.21** | **67.82** | **Ker** | **197** |
| **Not295** | **Not295** | **CEAMARC** | **Notasterias sp** | **Christopher L. Mah** | **MNHN IE-2013-1729** | **2008** |  |  | **AdL** |  |
| **Not282** | **Not282** | **CEAMARC** | **Notasterias sp** | **Christopher L. Mah** | **MNHN IE-2013-1732** | **2007** |  |  | **AdL** |  |
| **Not290** | **Not290** | **CEAMARC** | **Notasterias sp** | **Christopher L. Mah** | **MNHN IE-2013-1733** | **2008** |  |  | **AdL** |  |
| **BASEC282-12** | **Lys053** | **JR275** | **Lysasterias sp** | **Christopher L. Mah** | **MNHN IE-2013-221** | **2012** | **-75.76** | **-30.45** | **WS** | **428** |
| **Not163** | **Not163** | **ANT XXVII/3 (CAMBIO)** | **Notasterias armata** | **Christopher L. Mah** | **MNHN IE-2013-224** | **2011** | **-65.56** | **-61.62** | **AntPenEast** | **324** |
| **Not164** | **Not164** | **ANT XXVII/3 (CAMBIO)** | **Notasterias armata** | **Christopher L. Mah** | **MNHN IE-2013-224** | **2011** | **-65.56** | **-61.62** | **AntPenEast** | **324** |
| **BASEC287-12** | **Not162** | **JR275** | **Notasterias bongraini** | **Christopher L. Mah** | **MNHN IE-2013-229** | **2012** | **-75.75** | **-31.25** | **WS** | **585** |
| **Dip175** | **Dip175** | **JR262** | **Diplasterias brandti** | **Christopher L. Mah** | **MNHN IE-2013-241** | **2011** | **-60.93** | **-45.23** | **Sork** | **280** |
| **Dip176** | **Dip176** | **JR262** | **Diplasterias brandti** | **Christopher L. Mah** | **MNHN IE-2013-241** | **2011** | **-60.93** | **-45.23** | **Sork** | **280** |
| **BASEC263-12** | **Lys052** | **JR275** | **Lysasterias sp** | **Christopher L. Mah** | **MNHN IE-2013-244** | **2012** | **-76.32** | **-32.88** | **WS** | **780** |
| **BASEC264-12** | **Lys059** | **JR275** | **Lysasterias sp** | **Christopher L. Mah** | **MNHN IE-2013-245** | **2012** | **-76.32** | **-32.88** | **WS** | **780** |
| **Lys051** | **Lys051** | **JR262** | **Lysasterias sp** | **Christopher L. Mah** | **MNHN IE-2013-250b** | **2011** |  |  | **SG** |  |
| **Dip154** | **Dip154** | **ANT XXVII/3 (CAMBIO)** | **Diplasterias brucei** | **Christopher L. Mah** | **MNHN IE-2013-254** | **2011** | **-61.20** | **-45.76** | **Sork** | **324** |
| **Lys049** | **Lys049** | **JR262** | **Lysasterias sp** | **Christopher L. Mah** | **MNHN IE-2013-259** | **2011** | **-54.56** | **-39.03** | **SG** | **205** |
| **Dip167** | **Dip167** | **JR262** | **Diplasterias meridionalis** | **Christopher L. Mah** | **MNHN IE-2013-260** | **2011** | **-54.56** | **-39.03** | **SG** | **205** |
| **Dip168** | **Dip168** | **JR262** | **Diplasterias meridionalis** | **Christopher L. Mah** | **MNHN IE-2013-260** | **2011** | **-54.56** | **-39.03** | **SG** | **205** |
| **Dip169** | **Dip169** | **JR262** | **Diplasterias meridionalis** | **Christopher L. Mah** | **MNHN IE-2013-260** | **2011** | **-54.56** | **-39.03** | **SG** | **205** |
| **Psi044** | **Psi044** | **ANT XXVII/3 (CAMBIO)** | **Psilaster charcoti** | **Christopher L. Mah** | **MNHN IE-2013-261** | **2011** | **-54.50** | **3.23** | **Bouv** | **264** |
| **Dip155** | **Dip155** | **JR262** | **Diplasterias meridionalis** | **Christopher L. Mah** | **MNHN IE-2013-266** | **2011** | **-54.55** | **-39.03** | **SG** | **205** |
| **Dip174** | **Dip174** | **JR262** | **Lysasterias sp** | **Christopher L. Mah** | **MNHN IE-2013-267** | **2011** | **-54.55** | **-39.03** | **SG** | **205** |
| **Not165** | **Not165** | **JR262** | **Notasterias bongraini** | **Christopher L. Mah** | **MNHN IE-2013-271** | **2011** | **-54.98** | **-35.76** | **SG** | **140** |
| **Bat011** | **Bat011** | **JR262** | **Bathybiaster loripes** | **Christopher L. Mah** | **MNHN IE-2013-274** | **2011** | **-54.29** | **-36.08** | **SG** | **135** |
| **Not168** | **Not168** | **ANT XXVII/3 (CAMBIO)** | **Notasterias bongraini** | **Christopher L. Mah** | **MNHN IE-2013-275** | **2011** | **-66.20** | **-60.16** | **AntPenEast** | **356** |
| **Not169** | **Not169** | **ANT XXVII/3 (CAMBIO)** | **Notasterias bongraini** | **Christopher L. Mah** | **MNHN IE-2013-275** | **2011** | **-66.20** | **-60.16** | **AntPenEast** | **356** |
| **Not170** | **Not170** | **ANT XXVII/3 (CAMBIO)** | **Notasterias bongraini** | **Christopher L. Mah** | **MNHN IE-2013-275** | **2011** | **-66.20** | **-60.16** | **AntPenEast** | **356** |
| **Not171** | **Not171** | **ANT XXVII/3 (CAMBIO)** | **Notasterias bongraini** | **Christopher L. Mah** | **MNHN IE-2013-275** | **2011** | **-66.20** | **-60.16** | **AntPenEast** | **356** |
| **Not172** | **Not172** | **ANT XXVII/3 (CAMBIO)** | **Notasterias bongraini** | **Christopher L. Mah** | **MNHN IE-2013-275** | **2011** | **-66.20** | **-60.16** | **AntPenEast** | **356** |
| **Lys047** | **Lys047** | **ANT XXVII/3 (CAMBIO)** | **Lysasterias perrieri** | **Christopher L. Mah** | **MNHN IE-2013-279** | **2011** | **-66.20** | **-60.16** | **AntPenEast** | **356** |
| **Lys048** | **Lys048** | **ANT XXVII/3 (CAMBIO)** | **Lysasterias perrieri** | **Christopher L. Mah** | **MNHN IE-2013-279** | **2011** | **-66.20** | **-60.16** | **AntPenEast** | **356** |
| **Dip160** | **Dip160** | **ANT XXVII/3 (CAMBIO)** | **Diplasterias meridionalis** | **Christopher L. Mah** | **MNHN IE-2013-283** | **2011** | **-53.41** | **-42.68** | **Shag** | **309** |
| **Psi042** | **Psi042** | **JR262** | **Psilaster charcoti** | **Christopher L. Mah** | **MNHN IE-2013-290** | **2011** | **-55.04** | **-36.15** | **SG** | **160** |
| **Dip173** | **Dip173** | **JR262** | **Diplasterias meridionalis** | **Christopher L. Mah** | **MNHN IE-2013-294** | **2011** | **-54.56** | **-39.02** | **SG** | **205** |
| **Bat086** | **Bat086** | **JR262** | **Bathybiaster loripes** | **Christopher L. Mah** | **MNHN IE-2013-297** | **2011** | **-54.52** | **-38.85** | **SG** | **230** |
| **Lys054** | **Lys054** | **JR262** | **Lysasterias perrieri** | **Christopher L. Mah** | **MNHN IE-2013-299** | **2011** | **-60.72** | **-45.49** | **Sork** | **280** |
| **Dip162** | **Dip162** | **JR262** | **Diplasterias brandti** | **Christopher L. Mah** | **MNHN IE-2013-300** | **2011** | **-54.66** | **-35.01** | **SG** | **230** |
| **Dip163** | **Dip163** | **JR262** | **Diplasterias brandti** | **Christopher L. Mah** | **MNHN IE-2013-300** | **2011** | **-54.66** | **-35.01** | **SG** | **230** |
| **Dip164** | **Dip164** | **JR262** | **Diplasterias brandti** | **Christopher L. Mah** | **MNHN IE-2013-300** | **2011** | **-54.66** | **-35.01** | **SG** | **230** |
| **BASEC275-12** | **Dip171** | **JR275** | **Diplasterias brucei** | **Christopher L. Mah** | **MNHN IE-2013-304** | **2012** | **-75.26** | **-30.26** | **WS** | **418** |
| **Psi038** | **Psi038** | **ANT XXVII/3 (CAMBIO)** | **Psilaster charcoti** | **Christopher L. Mah** | **MNHN IE-2013-307** | **2011** | **-62.30** | **-58.68** | **Sshet** | **452** |
| **Dip157** | **Dip157** | **ANT XXVII/3 (CAMBIO)** | **Diplasterias brandti** | **Christopher L. Mah** | **MNHN IE-2013-315** | **2011** | **-70.82** | **-10.54** | **WS** | **283** |
| **Lys056** | **Lys056** | **ANT XXVII/3 (CAMBIO)** | **Lysasterias perrieri** | **Christopher L. Mah** | **MNHN IE-2013-325** | **2011** | **-70.84** | **-10.59** | **WS** | **268** |
| **Dip149** | **Dip149** | **ANT XXVII/3 (CAMBIO)** | **Diplasterias brucei** | **Christopher L. Mah** | **MNHN IE-2013-337** | **2011** | **-70.86** | **-10.59** | **WS** | **224** |
| **Psi040** | **Psi040** | **JR275** | **Psilaster charcoti** | **Christopher L. Mah** | **MNHN IE-2013-351** | **2012** | **-74.37** | **-28.11** | **WS** | **2052** |
| **BASEC430-12** | **Psi041** | **JR275** | **Psilaster charcoti** | **Christopher L. Mah** | **MNHN IE-2013-352** | **2012** | **-74.37** | **-28.11** | **WS** | **2052** |
| **Psi043** | **Psi043** | **JR275** | **Psilaster charcoti** | **Christopher L. Mah** | **MNHN IE-2013-355** | **2012** | **-74.37** | **-28.11** | **WS** | **2052** |
| **Psi039** | **Psi039** | **JR275** | **Psilaster charcoti** | **Christopher L. Mah** | **MNHN IE-2013-356** | **2012** | **-74.37** | **-28.11** | **WS** | **2052** |
| **Psi249** | **Psi249** | **JR275** | **Psilaster charcoti** | **Camille Moreau** | **MNHN IE-2013-356** | **2012** |  |  | **WS** |  |
| **Not166** | **Not166** | **ANT XXVII/3 (CAMBIO)** | **Notasterias bongraini** | **Christopher L. Mah** | **MNHN IE-2013-367** | **2011** | **-66.21** | **-60.16** | **AntPenEast** | **381** |
| **Dip153** | **Dip153** | **JR275** | **Diplasterias brucei** | **Christopher L. Mah** | **MNHN IE-2013-379** | **2012** | **-76.17** | **-27.80** | **WS** | **549** |
| **Dip172** | **Dip172** | **JR262** | **Diplasterias meridionalis** | **Christopher L. Mah** | **MNHN IE-2013-386** | **2011** | **-54.66** | **-34.97** | **SG** | **270** |
| **Dip156** | **Dip156** | **JR262** | **Diplasterias meridionalis** | **Christopher L. Mah** | **MNHN IE-2013-387** | **2011** | **-54.52** | **-38.85** | **SG** | **230** |
| **Bat012** | **Bat012** | **JR262** | **Bathybiaster loripes** | **Christopher L. Mah** | **MNHN IE-2013-388** | **2011** | **-54.52** | **-38.85** | **SG** | **230** |
| **Dip151** | **Dip151** | **JR262** | **Diplasterias meridionalis** | **Christopher L. Mah** | **MNHN IE-2013-391** | **2011** | **-53.83** | **-40.99** | **Shag** | **165** |
| **Lys312** | **Lys312** | **JR230** | **Lysasterias sp** | **Christopher L. Mah** | **MNHN IE-2013-403** | **2009** | **-67.08** | **-69.41** | **Belli** | **461.46** |
| **Bat153** | **Bat153** | **JR230** | **Bathybiaster loripes** | **Christopher L. Mah** | **MNHN IE-2013-404** | **2009** | **-67.83** | **-70.84** | **Belli** | **588.98** |
| **Bat154** | **Bat154** | **JR230** | **Bathybiaster loripes** | **Christopher L. Mah** | **MNHN IE-2013-406** | **2009** | **-67.17** | **-69.43** | **Belli** | **442.62** |
| **Bat156** | **Bat156** | **JR230** | **Bathybiaster loripes** | **Christopher L. Mah** | **MNHN IE-2013-418** | **2009** | **-67.54** | **-70.21** | **Belli** | **523** |
| **Dip318b** | **Dip318b** | **JR275** | **Diplasterias brucei** | **Christopher L. Mah** | **MNHN IE-2013-430** | **2012** | **-75.24** | **-29.00** | **WS** | **392** |
| **Bat152** | **Bat152** | **ANT XXVII/3 (CAMBIO)** | **Bathybiaster loripes** | **Christopher L. Mah** | **MNHN IE-2013-433** | **2011** | **-64.91** | **-60.61** | **AntPenEast** | **214** |
| **Not367** | **Not367** | **JR275** | **Notasterias bongraini** | **Christopher L. Mah** | **MNHN IE-2013-436** | **2012** | **-75.76** | **-30.44** | **WS** | **429** |
| **Not368** | **Not368** | **JR275** | **Notasterias bongraini** | **Christopher L. Mah** | **MNHN IE-2013-436** | **2012** | **-75.76** | **-30.44** | **WS** | **429** |
| **Not366** | **Not366** | **JR275** | **Notasterias armata** | **Christopher L. Mah** | **MNHN IE-2013-451** | **2012** | **-76.32** | **-32.87** | **WS** | **780** |
| **BASEC327-12** | **Lys304** | **JR275** | **Lysasterias perrieri** | **Christopher L. Mah** | **MNHN IE-2013-453** | **2012** | **-76.32** | **-32.87** | **WS** | **780** |
| **Not381** | **Not381** | **JR262** | **Notasterias bongraini** | **Christopher L. Mah** | **MNHN IE-2013-458** | **2011** | **-54.40** | **-37.37** | **SG** | **190** |
| **Not383** | **Not383** | **JR262** | **Notasterias bongraini** | **Christopher L. Mah** | **MNHN IE-2013-458** | **2011** | **-54.40** | **-37.37** | **SG** | **190** |
| **Not361** | **Not361** | **ANT XXVII/3 (CAMBIO)** | **Notasterias bongraini** | **Christopher L. Mah** | **MNHN IE-2013-459** | **2011** | **-65.96** | **-60.47** | **AntPenEast** | **202** |
| **Psi152** | **Psi152** | **ANT XXVII/3 (CAMBIO)** | **Bathybiaster loripes** | **Christopher L. Mah** | **MNHN IE-2013-472** | **2011** | **-54.47** | **3.19** | **Bouv** | **250** |
| **Not378** | **Not378** | **ANT XXVII/3 (CAMBIO)** | **Notasterias bongraini** | **Christopher L. Mah** | **MNHN IE-2013-496** | **2011** | **-64.93** | **-60.56** | **AntPenEast** | **324** |
| **Not379** | **Not379** | **ANT XXVII/3 (CAMBIO)** | **Notasterias bongraini** | **Christopher L. Mah** | **MNHN IE-2013-496** | **2011** | **-64.93** | **-60.56** | **AntPenEast** | **324** |
| **Not380** | **Not380** | **ANT XXVII/3 (CAMBIO)** | **Notasterias bongraini** | **Christopher L. Mah** | **MNHN IE-2013-496** | **2011** | **-64.93** | **-60.56** | **AntPenEast** | **324** |
| **Psi149** | **Psi149** | **ANT XXVII/3 (CAMBIO)** | **Psilaster charcoti** | **Christopher L. Mah** | **MNHN IE-2013-497** | **2011** | **-62.30** | **-58.67** | **Sshet** | **872** |
| **Psi148** | **Psi148** | **ANT XXVII/3 (CAMBIO)** | **Psilaster charcoti** | **Christopher L. Mah** | **MNHN IE-2013-499** | **2011** | **-62.30** | **-58.68** | **Sshet** | **459** |
| **Not359** | **Not359** | **ANT XXVII/3 (CAMBIO)** | **Notasterias sp** | **Christopher L. Mah** | **MNHN IE-2013-504** | **2011** | **-70.84** | **-10.60** | **WS** | **252** |
| **Not369** | **Not369** | **ANT XXVII/3 (CAMBIO)** | **Notasterias bongraini** | **Christopher L. Mah** | **MNHN IE-2013-505** | **2011** | **-70.84** | **-10.60** | **WS** | **252** |
| **Not370** | **Not370** | **ANT XXVII/3 (CAMBIO)** | **Notasterias bongraini** | **Christopher L. Mah** | **MNHN IE-2013-505** | **2011** | **-70.84** | **-10.60** | **WS** | **252** |
| **Lys308** | **Lys308** | **JR275** | **Lysasterias perrieri** | **Christopher L. Mah** | **MNHN IE-2013-517** | **2012** | **-75.26** | **-31.13** | **WS** | **604** |
| **Not373** | **Not373** | **JR275** | **Notasterias bongraini** | **Christopher L. Mah** | **MNHN IE-2013-519** | **2012** | **-75.26** | **-31.13** | **WS** | **604** |
| **Not374** | **Not374** | **JR275** | **Notasterias bongraini** | **Christopher L. Mah** | **MNHN IE-2013-519** | **2012** | **-75.26** | **-31.13** | **WS** | **604** |
| **Not375** | **Not375** | **JR275** | **Notasterias bongraini** | **Christopher L. Mah** | **MNHN IE-2013-519** | **2012** | **-75.26** | **-31.13** | **WS** | **604** |
| **Bat281** | **Bat281** | **ACE_2017** | **Bathybiaster loripes** | **Camille Moreau** | **MNHN IE-2013-522** | **2017** | **-66.15** | **162.20** | **Bal** |  |
| **Psi153** | **Psi153** | **JR275** | **Psilaster charcoti** | **Christopher L. Mah** | **MNHN IE-2013-523** | **2012** | **-74.51** | **-28.75** | **WS** | **1558** |
| **Psi154** | **Psi154** | **JR275** | **Psilaster charcoti** | **Christopher L. Mah** | **MNHN IE-2013-523** | **2012** | **-74.51** | **-28.75** | **WS** | **1558** |
| **Bat157** | **Bat157** | **JR275** | **Bathybiaster loripes** | **Christopher L. Mah** | **MNHN IE-2013-525** | **2012** | **-74.51** | **-28.75** | **WS** | **1558** |
| **Dip323b** | **Dip323b** | **ANT XXVII/3 (CAMBIO)** | **Diplasterias brucei** | **Christopher L. Mah** | **MNHN IE-2013-531** | **2011** | **-70.79** | **-10.67** | **WS** | **630** |
| **Dip324b** | **Dip324b** | **ANT XXVII/3 (CAMBIO)** | **Diplasterias brucei** | **Christopher L. Mah** | **MNHN IE-2013-531** | **2011** | **-70.79** | **-10.67** | **WS** | **630** |
| **Psi151** | **Psi151** | **ANT XXVII/3 (CAMBIO)** | **Psilaster charcoti** | **Christopher L. Mah** | **MNHN IE-2013-535** | **2011** | **-70.79** | **-10.67** | **WS** | **630** |
| **Lys311** | **Lys311** | **ANT XXVII/3 (CAMBIO)** | **Lysasterias perrieri** | **Christopher L. Mah** | **MNHN IE-2013-539** | **2011** | **-70.84** | **-10.59** | **WS** | **252** |
| **Not377** | **Not377** | **ANT XXVII/3 (CAMBIO)** | **Notasterias bongraini** | **Christopher L. Mah** | **MNHN IE-2013-545** | **2011** | **-70.84** | **-10.60** | **WS** | **248** |
| **Bat155** | **Bat155** | **JR262** | **Bathybiaster loripes** | **Christopher L. Mah** | **MNHN IE-2013-555** | **2011** | **-54.52** | **-38.85** | **SG** | **230** |
| **Bat158** | **Bat158** | **ANT XXVII/3 (CAMBIO)** | **Bathybiaster loripes** | **Christopher L. Mah** | **MNHN IE-2013-559** | **2011** | **-61.18** | **-45.73** | **Sork** | **338** |
| **Bat094** | **Bat094** | **ANT XXVII/3 (CAMBIO)** | **Bathybiaster loripes** | **Christopher L. Mah** | **MNHN IE-2013-566** | **2011** | **-61.15** | **-44.00** | **Sork** | **389** |
| **Dip322b** | **Dip322b** | **JR262** | **Diplasterias meridionalis** | **Christopher L. Mah** | **MNHN IE-2013-598** | **2011** | **-54.66** | **-34.95** | **SG** | **290** |
| **Not371** | **Not371** | **ANT XXVII/3 (CAMBIO)** | **Notasterias bongraini** | **Christopher L. Mah** | **MNHN IE-2013-599** | **2011** | **-65.93** | **-60.33** | **AntPenEast** | **429** |
| **Not372** | **Not372** | **ANT XXVII/3 (CAMBIO)** | **Notasterias bongraini** | **Christopher L. Mah** | **MNHN IE-2013-599** | **2011** | **-65.93** | **-60.33** | **AntPenEast** | **429** |
| **Lys036** | **Lys036** | **REVOLTA1** | **Lysasterias sp** | **Christopher L. Mah** | **MNHN IE-2013-60** | **2010** | **-66.64** | **140.03** | **AdL** | **85** |
| **Lys037** | **Lys037** | **REVOLTA1** | **Lysasterias sp** | **Christopher L. Mah** | **MNHN IE-2013-60** | **2010** | **-66.64** | **140.03** | **AdL** | **85** |
| **Lys038** | **Lys038** | **REVOLTA1** | **Lysasterias sp** | **Christopher L. Mah** | **MNHN IE-2013-60** | **2010** | **-66.64** | **140.03** | **AdL** | **85** |
| **Lys040** | **Lys040** | **REVOLTA1** | **Lysasterias sp** | **Christopher L. Mah** | **MNHN IE-2013-60** | **2010** | **-66.64** | **140.03** | **AdL** | **85** |
| **BASEC402-12** | **Not360** | **JR275** | **Notasterias bongraini** | **Christopher L. Mah** | **MNHN IE-2013-626** | **2012** | **-74.67** | **-29.42** | **WS** | **587** |
| **BASEC401-12** | **Not365** | **JR275** | **Notasterias armata** | **Christopher L. Mah** | **MNHN IE-2013-627** | **2012** | **-74.67** | **-29.42** | **WS** | **587** |
| **BASEC403-12** | **Not362** | **JR275** | **Notasterias bongraini** | **Christopher L. Mah** | **MNHN IE-2013-638** | **2012** | **-74.67** | **-29.42** | **WS** | **587** |
| **BASEC404-12** | **Not363** | **JR275** | **Notasterias armata** | **Christopher L. Mah** | **MNHN IE-2013-639** | **2012** | **-74.67** | **-29.42** | **WS** | **587** |
| **BASEC377-12** | **Psi150** | **JR275** | **Psilaster charcoti** | **Christopher L. Mah** | **MNHN IE-2013-648** | **2012** | **-74.50** | **-28.74** | **WS** | **1580** |
| **BASEC348-12** | **Lys309** | **JR275** | **Lysasterias perrieri** | **Christopher L. Mah** | **MNHN IE-2013-652** | **2012** | **-76.20** | **-31.86** | **WS** | **576** |
| **Lys310** | **Lys310** | **JR275** | **Lysasterias perrieri** | **Christopher L. Mah** | **MNHN IE-2013-653** | **2012** | **-76.20** | **-31.86** | **WS** | **576** |
| **Dip206** | **Dip206** | **JR275** | **Diplasterias brucei** | **Christopher L. Mah** | **MNHN IE-2013-666** | **2012** | **-76.17** | **-27.80** | **WS** | **545** |
| **Dip207** | **Dip207** | **JR262** | **Diplasterias meridionalis** | **Christopher L. Mah** | **MNHN IE-2013-669** | **2011** | **-54.56** | **-39.02** | **SG** | **205** |
| **Dip208** | **Dip208** | **JR262** | **Diplasterias meridionalis** | **Christopher L. Mah** | **MNHN IE-2013-669** | **2011** | **-54.56** | **-39.02** | **SG** | **205** |
| **Dip209** | **Dip209** | **JR262** | **Diplasterias meridionalis** | **Christopher L. Mah** | **MNHN IE-2013-669** | **2011** | **-54.56** | **-39.02** | **SG** | **205** |
| **Dip205** | **Dip205** | **JR262** | **Diplasterias meridionalis** | **Christopher L. Mah** | **MNHN IE-2013-670** | **2011** | **-54.56** | **-39.02** | **SG** | **205** |
| **BASEC250-12** | **Psi122** | **JR275** | **Psilaster charcoti** | **Christopher L. Mah** | **MNHN IE-2013-680** | **2012** | **-75.25** | **-29.03** | **WS** | **390** |
| **Lys175** | **Lys175** | **JR262** | **Lysasterias sp** | **Christopher L. Mah** | **MNHN IE-2013-689** | **2011** | **-55.00** | **-37.29** | **SG** | **145** |
| **Not226** | **Not226** | **JR262** | **Notasterias bongraini** | **Christopher L. Mah** | **MNHN IE-2013-693** | **2011** | **-54.40** | **-37.38** | **SG** | **175** |
| **Not227** | **Not227** | **JR262** | **Notasterias bongraini** | **Christopher L. Mah** | **MNHN IE-2013-693** | **2011** | **-54.40** | **-37.38** | **SG** | **175** |
| **Not228** | **Not228** | **JR262** | **Notasterias bongraini** | **Christopher L. Mah** | **MNHN IE-2013-693** | **2011** | **-54.40** | **-37.38** | **SG** | **175** |
| **Not225** | **Not225** | **JR262** | **Notasterias armata** | **Christopher L. Mah** | **MNHN IE-2013-696** | **2011** | **-55.17** | **-35.48** | **SG** | **130** |
| **Lys179** | **Lys179** | **JR262** | **Lysasterias perrieri** | **Christopher L. Mah** | **MNHN IE-2013-698** | **2011** | **-55.17** | **-35.48** | **SG** | **130** |
| **Lys173** | **Lys173** | **JR275** | **Lysasterias perrieri** | **Christopher L. Mah** | **MNHN IE-2013-700** | **2012** | **-75.25** | **-29.03** | **WS** | **390** |
| **BASEC358-12** | **Not233** | **JR275** | **Notasterias sp** | **Christopher L. Mah** | **MNHN IE-2013-706** | **2012** | **-75.25** | **-29.03** | **WS** | **390** |
| **BASEC356-12** | **Not235** | **JR275** | **Notasterias sp** | **Christopher L. Mah** | **MNHN IE-2013-708** | **2012** | **-75.25** | **-29.03** | **WS** | **390** |
| **Lys171** | **Lys171** | **JR275** | **Lysasterias sp** | **Christopher L. Mah** | **MNHN IE-2013-709** | **2012** | **-76.33** | **-32.90** | **WS** | **779** |
| **BASEC331-12** | **Not229** | **JR275** | **Notasterias sp** | **Christopher L. Mah** | **MNHN IE-2013-710** | **2012** | **-76.33** | **-32.90** | **WS** | **779** |
| **BASEC333-12** | **Lys178** | **JR275** | **Lysasterias sp** | **Christopher L. Mah** | **MNHN IE-2013-712** | **2012** | **-76.33** | **-32.90** | **WS** | **779** |
| **BASEC334-12** | **Lys174** | **JR275** | **Lysasterias sp** | **Christopher L. Mah** | **MNHN IE-2013-713** | **2012** | **-76.33** | **-32.90** | **WS** | **779** |
| **BASEC336-12** | **Lys172** | **JR275** | **Lysasterias sp** | **Christopher L. Mah** | **MNHN IE-2013-715** | **2012** | **-76.33** | **-32.90** | **WS** | **779** |
| **Bat096** | **Bat096** | **ANT XXVII/3 (CAMBIO)** | **Bathybiaster loripes** | **Christopher L. Mah** | **MNHN IE-2013-718** | **2011** | **-61.16** | **-44.04** | **Sork** | **354** |
| **Lys170** | **Lys170** | **ANT XXVII/3 (CAMBIO)** | **Lysasterias perrieri** | **Christopher L. Mah** | **MNHN IE-2013-724** | **2011** | **-54.48** | **3.19** | **Bouv** | **300** |
| **Psi120** | **Psi120** | **ANT XXVII/3 (CAMBIO)** | **Psilaster charcoti** | **Christopher L. Mah** | **MNHN IE-2013-726** | **2011** | **-54.48** | **3.19** | **Bouv** | **300** |
| **Psi121** | **Psi121** | **ANT XXVII/3 (CAMBIO)** | **Psilaster charcoti** | **Christopher L. Mah** | **MNHN IE-2013-726** | **2011** | **-54.48** | **3.19** | **Bouv** | **300** |
| **Not232** | **Not232** | **JR275** | **Notasterias armata** | **Christopher L. Mah** | **MNHN IE-2013-730** | **2012** | **-75.27** | **-31.16** | **WS** | **614** |
| **BASEC363-12** | **Not231** | **JR275** | **Notasterias armata** | **Christopher L. Mah** | **MNHN IE-2013-731** | **2012** | **-75.27** | **-31.16** | **WS** | **614** |
| **Dip204** | **Dip204** | **ANT XXVII/3 (CAMBIO)** | **Diplasterias meridionalis** | **Christopher L. Mah** | **MNHN IE-2013-736** | **2011** | **-53.41** | **-42.67** | **Shag** | **315** |
| **Lys177** | **Lys177** | **JR262** | **Lysasterias sp** | **Christopher L. Mah** | **MNHN IE-2013-737** | **2011** | **-55.04** | **-36.16** | **SG** | **160** |
| **Dip203** | **Dip203** | **JR262** | **Diplasterias meridionalis** | **Christopher L. Mah** | **MNHN IE-2013-738** | **2011** | **-55.04** | **-36.16** | **SG** | **160** |
| **Dip211** | **Dip211** | **JR262** | **Diplasterias meridionalis** | **Christopher L. Mah** | **MNHN IE-2013-739** | **2011** | **-54.28** | **-36.08** | **SG** | **125** |
| **Lys180** | **Lys180** | **ANT XXVII/3 (CAMBIO)** | **Lysasterias perrieri** | **Christopher L. Mah** | **MNHN IE-2013-744** | **2011** | **-70.84** | **-10.59** | **WS** | **268** |
| **Lys181** | **Lys181** | **ANT XXVII/3 (CAMBIO)** | **Lysasterias perrieri** | **Christopher L. Mah** | **MNHN IE-2013-744** | **2011** | **-70.84** | **-10.59** | **WS** | **268** |
| **Not230** | **Not230** | **ANT XXVII/3 (CAMBIO)** | **Notasterias bongraini** | **Christopher L. Mah** | **MNHN IE-2013-753** | **2011** | **-70.84** | **-10.59** | **WS** | **268** |
| **Dip451** | **Dip451** | **JR144** | **Diplasterias sp** |  | **MNHN IE-2013-8346** | **2006** |  |  | **Mag** |  |
| **Bat097** | **Bat097** | **ANT XXVII/3 (CAMBIO)** | **Bathybiaster loripes** | **Christopher L. Mah** | **MNHN IE-2013-963** | **2011** | **-61.16** | **-44.04** | **Sork** | **354** |
| **Bat098** | **Bat098** | **ANT XXVII/3 (CAMBIO)** | **Bathybiaster loripes** | **Christopher L. Mah** | **MNHN IE-2013-964** | **2011** | **-61.16** | **-44.04** | **Sork** | **354** |
| **Bat087** | **Bat087** | **JR262** | **Bathybiaster loripes** | **Christopher L. Mah** | **MNHN IE-2013-967** | **2011** | **-54.52** | **-38.85** | **SG** | **230** |
| **Bat088** | **Bat088** | **JR262** | **Bathybiaster loripes** | **Christopher L. Mah** | **MNHN IE-2013-968** | **2011** | **-54.52** | **-38.85** | **SG** | **230** |
| **Bat089** | **Bat089** | **JR262** | **Bathybiaster loripes** | **Christopher L. Mah** | **MNHN IE-2013-969** | **2011** | **-54.52** | **-38.85** | **SG** | **230** |
| **Bat090** | **Bat090** | **JR262** | **Bathybiaster loripes** | **Christopher L. Mah** | **MNHN IE-2013-970** | **2011** | **-54.52** | **-38.85** | **SG** | **230** |
| **Bat091** | **Bat091** | **JR262** | **Bathybiaster loripes** | **Christopher L. Mah** | **MNHN IE-2013-971** | **2011** | **-54.52** | **-38.85** | **SG** | **230** |
| **Bat095** | **Bat095** | **ANT XXVII/3 (CAMBIO)** | **Bathybiaster loripes** | **Christopher L. Mah** | **MNHN IE-2013-972** | **2011** |  |  |  |  |
| **Bat099** | **Bat099** | **ANT XXVII/3 (CAMBIO)** | **Bathybiaster loripes** | **Christopher L. Mah** | **MNHN IE-2013-973** | **2011** |  |  |  |  |
| **Bat100** | **Bat100** | **ANT XXVII/3 (CAMBIO)** | **Bathybiaster loripes** | **Christopher L. Mah** | **MNHN IE-2013-974** | **2011** |  |  |  |  |
| **Bat101** | **Bat101** | **ANT XXVII/3 (CAMBIO)** | **Bathybiaster loripes** | **Christopher L. Mah** | **MNHN IE-2013-975** | **2011** |  |  |  |  |
| **Bat102** | **Bat102** | **ANT XXVII/3 (CAMBIO)** | **Bathybiaster loripes** | **Christopher L. Mah** | **MNHN IE-2013-976** | **2011** |  |  |  |  |
| **Bat103** | **Bat103** | **ANT XXVII/3 (CAMBIO)** | **Bathybiaster loripes** | **Christopher L. Mah** | **MNHN IE-2013-977** | **2011** |  |  |  |  |
| **Bat104** | **Bat104** | **ANT XXVII/3 (CAMBIO)** | **Bathybiaster loripes** | **Christopher L. Mah** | **MNHN IE-2013-978** | **2011** |  |  |  |  |
| **AIIIS004-15** | **Dip095** | **ANDEEP-III** | **Diplasterias brucei** | **Camille Moreau** |  | **2005** | **-71.31** | **-14.01** | **WS** | **1036** |
| **AIIIS005-15** | **Dip096** | **ANDEEP-III** | **Diplasterias brucei** | **Camille Moreau** |  | **2005** | **-71.31** | **-14.01** | **WS** | **1036** |
| **AIIIS010-15** | **Bat223** | **ANDEEP-III** | **Bathybiaster loripes** | **Camille Moreau** |  | **2005** | **-63.35** | **-64.61** | **Sshet** | **2124** |
| **AIIIS011-15** | **Bat224** | **ANDEEP-III** | **Bathybiaster loripes** | **Camille Moreau** |  | **2005** | **-63.35** | **-64.61** | **Sshet** | **2124** |
| **AIIIS012-15** | **Bat225** | **ANDEEP-III** | **Bathybiaster loripes** | **Camille Moreau** |  | **2005** | **-63.35** | **-64.61** | **Sshet** | **2124** |
| **AIIIS014-15** | **Psi198** | **ANDEEP-III** | **Psilaster charcoti** | **Camille Moreau** |  | **2005** | **-63.35** | **-64.61** | **Sshet** | **2124** |
| **AIIIS015-15** | **Psi199** | **ANDEEP-III** | **Psilaster charcoti** | **Camille Moreau** |  | **2005** | **-63.35** | **-64.61** | **Sshet** | **2124** |
| **AIIIS018-15** | **Psi197** | **ANDEEP-III** | **Psilaster charcoti** | **Camille Moreau** |  | **2005** | **-63.35** | **-64.61** | **Sshet** | **2124** |
| **AIIIS022-15** | **Bat228** | **ANDEEP-III** | **Bathybiaster loripes** | **Camille Moreau** |  | **2005** | **-71.17** | **-14.09** | **WS** | **2147** |
| **AIIIS054-15** | **Bat226** | **ANDEEP-III** | **Bathybiaster loripes** | **Camille Moreau** |  | **2005** | **-69.38** | **-5.25** | **WS** | **1822** |
| **AIIIS061-15** | **Bat277** | **ANDEEP-III** | **Bathybiaster loripes** | **Camille Moreau** |  | **2005** | **-69.38** | **-5.25** | **WS** | **1822** |
| **AIIIS063-15** | **Bat268** | **ANDEEP-III** | **Bathybiaster loripes** | **Camille Moreau** |  | **2005** | **-69.38** | **-5.25** | **WS** | **1822** |
| **AIIIS064-15** | **Bat263** | **ANDEEP-III** | **Bathybiaster loripes** | **Camille Moreau** |  | **2005** | **-69.38** | **-5.25** | **WS** | **1822** |
| **AIIIS065-15** | **Bat264** | **ANDEEP-III** | **Bathybiaster loripes** | **Camille Moreau** |  | **2005** | **-69.38** | **-5.25** | **WS** | **1822** |
| **AIIIS067-15** | **Not025** | **ANDEEP-III** | **Notasterias pedicellaris** | **Camille Moreau** |  | **2005** | **-71.17** | **-14.09** | **WS** | **2147** |
| **AIIIS069-15** | **Psi196** | **ANDEEP-III** | **Psilaster charcoti** | **Camille Moreau** |  | **2005** | **-63.62** | **-50.75** | **TipPen** | **2617** |
| **AIIIS098-15** | **Lys004** | **ANDEEP-SYSTCO** | **Lysasterias sp** | **Camille Moreau** |  | **2008** | **-70.40** | **-8.33** | **WS** | **602** |
| **AIIIS144-15** | **Not037** | **ANDEEP-SYSTCO** | **Notasterias sp** | **Camille Moreau** |  | **2008** | **-70.40** | **-8.33** | **WS** | **602** |
| **AIIIS145-15** | **Not038** | **ANDEEP-SYSTCO** | **Notasterias sp** | **Camille Moreau** |  | **2008** | **-70.40** | **-8.33** | **WS** | **602** |
| **AIIIS146-15** | **Not039** | **ANDEEP-SYSTCO** | **Notasterias sp** | **Camille Moreau** |  | **2008** | **-70.40** | **-8.33** | **WS** | **602** |
| **AIIIS147-15** | **Not040** | **ANDEEP-SYSTCO** | **Notasterias sp** | **Camille Moreau** |  | **2008** | **-70.40** | **-8.33** | **WS** | **602** |
| **AIIIS148-15** | **Not041** | **ANDEEP-SYSTCO** | **Notasterias sp** | **Camille Moreau** |  | **2008** | **-70.40** | **-8.33** | **WS** | **602** |
| **ASTAN353-10** |  | **CEAMARC** | **Odinella nutrix** | **Christopher L. Mah** |  | **2008** |  |  | **AdL** |  |
| **ASTAN478-10** |  | **CEAMARC** | **Misidentified** | **Christopher L. Mah** |  | **2008** |  |  | **AdL** |  |
| **ASTAN555-10** |  | **CEAMARC** | **Misidentified** | **Christopher L. Mah** |  | **2008** |  |  | **AdL** | **472** |
| **BASEC009-09/SOA631-12** | **Dip188** | **JR144** | **Diplasterias brucei** | **Christopher L. Mah** |  | **2006** | **-59.47** | **-27.28** | **Ssand** | **290** |
| **BASEC015-09/SOA590-12** | **Bat068** | **JR144** | **Bathybiaster loripes** | **Christopher L. Mah** |  | **2006** | **-62.95** | **-60.63** | **Sshet** | **150** |
| **BASEC020-09/SOA612-12** | **Bat069** | **JR144** | **Bathybiaster loripes** | **Christopher L. Mah** |  | **2006** | **-62.28** | **-61.60** | **Sshet** | **1511** |
| **BASEC024-09/SOA605-12** | **Psi106** | **JR144** | **Psilaster charcoti** | **Christopher L. Mah** |  | **2006** | **-59.52** | **-27.44** | **Ssand** | **1545** |
| **BASEC028-09/SOA638-12** | **Bat093** | **JR179** | **Bathybiaster loripes** | **Christopher L. Mah** |  | **2008** | **-74.48** | **-104.26** | **Amund** | **1037.05** |
| **BASEC031-09/SOA622-12** | **Psi105** | **JR179** | **Psilaster charcoti** | **Christopher L. Mah** |  | **2008** | **-73.97** | **-107.42** | **Amund** | **552.75** |
| **BASEC032-09/SOA643-12** | **Psi115** | **JR179** | **Psilaster charcoti** | **Christopher L. Mah** |  | **2008** | **-73.97** | **-107.42** | **Amund** | **552.75** |
| **BASEC047-09/SOA748-12** | **Not181** | **JR179** | **Notasterias armata** | **Christopher L. Mah** |  | **2008** | **-71.35** | **-110.01** | **Amund** | **476.49** |
| **BASEC321-12** |  | **JR275** |  | **Christopher L. Mah** |  | **2012** |  |  | **WS** |  |
| **BASEC428-12** |  | **JR275** | **Misidentified** | **Christopher L. Mah** |  | **2012** |  |  | **WS** | **2052** |
| **CHEC060-12** |  |  | **Psilaster andromeda** | **Kara K. S. Layton** |  | **2010** | **71.09** | **-135.58** | **Arctic** | **886** |
| **CHEC074-12** |  |  | **Psilaster andromeda** | **Kara K. S. Layton** |  | **2010** | **71.03** | **-134.82** | **Arctic** | **440** |
| **CHEC077-12** |  |  | **Psilaster andromeda** | **Kara K. S. Layton** |  | **2010** | **71.16** | **-135.56** | **Arctic** | **981** |
| **CHONE012-10** |  |  | **Psilaster andromeda** | **Kara K. S. Layton** |  | **2009** |  |  | **Arctic** | **666** |
| **DSPEC173-08** |  | **Royal British Columbia Museum** | **Psilaster pectinatus** | **Philip Lambert** |  | **2006** | **52.41** | **-132.21** | **Arctic** | **2200** |
| **DSPEC176-08** |  | **Royal British Columbia Museum** | **Psilaster pectinatus** | **Philip Lambert** |  | **2006** | **50.02** | **-128.85** | **Arctic** | **2025** |
| **DSPEC177-08** |  | **Royal British Columbia Museum** | **Psilaster pectinatus** | **Philip Lambert** |  | **2003** | **49.34** | **-127.53** | **Arctic** | **1804** |
| **EAR005-07** |  |  | **Coscinasterias muricata** | **Felicity McEnnulty** |  | **2007** | **-42.98** | **147.32** | **Aus** | **1** |
| **GENBANKHM542995.1** |  |  | **Thrissacanthias penicillatus** |  |  |  |  |  |  |  |
| **NZEC380** | **PsiNZEC380** | **NZEC** | **Psilaster acuminatus** | **Kate Neill** |  | **2007** | **-44.13** | **174.84** | **NZ** | **516** |
| **NZEC389** | **PsiNZEC389** | **NZEC** | **Psilaster acuminatus** | **Kate Neill** |  | **2006** | **-41.30** | **176.56** | **NZ** | **731** |
| **NZEC390** | **PsiNZEC390** | **NZEC** | **Psilaster acuminatus** | **Kate Neill** |  | **2007** | **-43.84** | **-178.59** | **NZ** | **460** |
| **NZEC397** | **PsiNZEC397** | **NZEC** | **Psilaster acuminatus** | **Kate Neill** |  | **2007** | **-42.62** | **175.92** | **NZ** | **1202** |
| **NZEC401** | **PsiNZEC401** | **NZEC** | **Psilaster acuminatus** | **Kate Neill** |  | **2007** | **-39.09** | **169.34** | **NZ** | **522** |
| **NZEC407** | **NZEC407** | **NZEC** | **Diplasterias brucei** | **Kate Neill** |  | **2004** | **-71.77** | **171.17** | **Ross** | **245** |
| **NZEC444** | **PsiNZEC444** | **NZEC** | **Psilaster charcoti** | **Kate Neill** |  | **2004** | **-71.53** | **171.41** | **Ross** | **385** |
| **NZEC448** |  | **NZEC** | **Bathybiaster loripes** | **Kate Neill** |  | **2006** | **-67.23** | **164.71** | **Bal** | **103** |
| **NZEC449** | **PsiNZEC449** | **NZEC** | **Psilaster charcoti** | **Kate Neill** |  | **2006** | **-66.70** | **163.18** | **Bal** | **70** |
| **NZEC450** | **PsiNZEC450** | **NZEC** | **Psilaster charcoti** | **Kate Neill** |  | **2004** | **-72.34** | **170.50** | **Ross** | **328** |
| **NZEC452** | **PsiNZEC452** | **NZEC** | **Psilaster charcoti** | **Kate Neill** |  |  | **-66.96** | **163.22** | **Bal** | **199** |
| **NZEC453** | **PsiNZEC453** | **NZEC** | **Psilaster charcoti** | **Kate Neill** |  | **2006** | **-66.56** | **162.57** | **Bal** | **63** |
| **NZEC685** | **NZEC685** | **NZEC** | **Notasterias stolophora** | **Kate Neill** |  | **2008** | **-73.12** | **174.32** | **Ross** | **321** |
| **NZEC686** | **NZEC686** | **NZEC** | **Diplasterias brucei** | **Kate Neill** |  | **2008** | **-73.12** | **174.32** | **Ross** | **321** |
| **NZEC687** | **NZEC687** | **NZEC** | **Diplasterias brucei** | **Kate Neill** |  | **2008** | **-74.11** | **170.80** | **Ross** | **639** |
| **NZEC688** | **NZEC688** | **NZEC** | **Diplasterias brucei** | **Kate Neill** |  | **2008** | **-74.59** | **170.28** | **Ross** | **283** |
| **NZEC689** | **NZEC689** | **NZEC** | **Notasterias armata** | **Kate Neill** |  | **2008** | **-73.12** | **174.32** | **Ross** | **321** |
| **NZEC690** | **NZEC690** | **NZEC** | **Notasterias armata** | **Kate Neill** |  | **2008** | **-75.63** | **169.85** | **Ross** | **525** |
| **NZEC691** | **NZEC691** | **NZEC** | **Notasterias armata** | **Kate Neill** |  | **2008** | **-76.78** | **167.84** | **Ross** | **724** |
| **NZEC692** | **NZEC692** | **NZEC** | **Notasterias armata** | **Kate Neill** |  | **2008** | **-73.25** | **178.72** | **Ross** | **760** |
| **NZEC693** | **NZEC693** | **NZEC** | **Notasterias armata** | **Kate Neill** |  | **2008** | **-74.11** | **170.80** | **Ross** | **639** |
| **NZEC694** | **NZEC694** | **NZEC** | **Notasterias armata** | **Kate Neill** |  | **2008** | **-74.73** | **167.01** | **Ross** | **916** |
| **NZECA197** | **PsiNZECA197** | **NZECA** | **Psilaster charcoti** | **Kate Neill** |  | **2006** | **-66.76** | **163.06** | **Bal** | **140** |
| **NZECA198** | **PsiNZECA198** | **NZECA** | **Psilaster charcoti** | **H. E. S. Clark** |  | **1965** | **-67.55** | **165.23** | **Bal** | **646** |
| **NZECA200** | **PsiNZECA200** | **NZECA** | **Psilaster charcoti** | **Kate Neill** |  | **2008** | **-72.08** | **172.90** | **Ross** |  |
| **NZECA201** | **NZECA201** | **NZECA** | **Psilaster charcoti** | **Kate Neill** |  | **2008** | **-72.08** | **172.90** | **Ross** |  |
| **NZECA202** |  | **NZECA** | **Bathybiaster loripes** | **Kate Neill** |  | **1964** | **-66.69** | **163.23** | **Bal** | **554** |
| **NZECA203** | **PsiNZECA203** | **NZECA** | **Psilaster charcoti** | **Kate Neill** |  | **2008** | **-76.59** | **176.83** | **Ross** |  |
| **NZECA204** | **PsiNZECA204** | **NZECA** | **Psilaster charcoti** | **Kate Neill** |  | **2008** | **-76.59** | **176.83** | **Ross** |  |
| **NZECA205** | **PsiNZECA205** | **NZECA** | **Psilaster charcoti** | **Kate Neill** |  | **2008** | **-76.20** | **176.25** | **Ross** |  |
| **NZECA206** | **PsiNZECA206** | **NZECA** | **Psilaster charcoti** | **Kate Neill** |  | **2008** | **-76.20** | **176.25** | **Ross** |  |
| **NZECA207** | **PsiNZECA207** | **NZECA** | **Psilaster charcoti** | **Kate Neill** |  | **1965** | **-67.58** | **164.88** | **Bal** |  |
| **NZECA208** | **PsiNZECA208** | **NZECA** | **Psilaster charcoti** | **Kate Neill** |  | **1965** | **-67.58** | **164.88** | **Bal** |  |
| **NZECA209** | **PsiNZECA209** | **NZECA** | **Psilaster charcoti** | **Kate Neill** |  | **2008** | **-72.32** | **175.49** | **Ross** |  |
| **NZECA210** | **PsiNZECA210** | **NZECA** | **Psilaster charcoti** | **Kate Neill** |  | **2008** | **-72.34** | **175.53** | **Ross** |  |
| **NZECA211** | **PsiNZECA211** | **NZECA** | **Psilaster charcoti** | **Kate Neill** |  | **2008** | **-76.59** | **176.83** | **Ross** |  |
| **NZECA212** | **PsiNZECA212** | **NZECA** | **Psilaster charcoti** | **Kate Neill** |  | **2008** | **-75.62** | **167.32** | **Ross** |  |
| **NZECA213** | **PsiNZECA213** | **NZECA** | **Psilaster charcoti** | **Kate Neill** |  | **2008** | **-75.62** | **167.32** | **Ross** |  |
| **NZECA214** | **PsiNZECA214** | **NZECA** | **Psilaster charcoti** | **Kate Neill** |  | **2008** | **-76.83** | **179.95** | **Ross** |  |
| **NZECA215** | **PsiNZECA215** | **NZECA** | **Psilaster charcoti** | **Kate Neill** |  | **2008** | **-76.83** | **179.95** | **Ross** |  |
| **NZECA217** | **PsiNZECA217** | **NZECA** | **Psilaster charcoti** | **Kate Neill** |  | **1964** | **-66.96** | **163.20** | **Bal** | **236** |
| **NZECA220** | **PsiNZECA220** | **NZECA** | **Psilaster charcoti** | **Kate Neill** |  | **1965** | **-67.58** | **164.88** | **Bal** | **278** |
| **NZECA223** | **NZECA223** | **NZECA** | **Bathybiaster loripes** | **Kate Neill** |  | **2006** | **-67.23** | **164.71** | **Ross** |  |
| **NZECA224** |  | **NZECA** | **Bathybiaster loripes** | **Kate Neill** |  | **2008** | **-75.62** | **169.80** | **Ross** |  |
| **NZECA225** |  | **NZECA** | **Bathybiaster loripes** | **Kate Neill** |  | **2008** | **-75.62** | **169.80** | **Ross** |  |
| **NZECA226** |  | **NZECA** | **Bathybiaster loripes** | **Kate Neill** |  | **2008** | **-76.60** | **176.80** | **Ross** |  |
| **NZECA227** |  | **NZECA** | **Bathybiaster loripes** | **Kate Neill** |  | **2008** | **-76.60** | **176.80** | **Ross** |  |
| **NZECA228** |  | **NZECA** | **Bathybiaster loripes** | **Kate Neill** |  | **2008** | **-76.20** | **176.25** | **Ross** |  |
| **NZECA229** |  | **NZECA** | **Bathybiaster loripes** | **Kate Neill** |  | **2008** | **-76.83** | **179.95** | **Ross** |  |
| **NZECA231** |  | **NZECA** | **Bathybiaster loripes** | **Kate Neill** |  | **2008** | **-75.62** | **167.32** | **Ross** |  |
| **NZECA232** |  | **NZECA** | **Bathybiaster loripes** | **Kate Neill** |  | **2008** | **-75.62** | **167.32** | **Ross** |  |
| **NZECA233** |  | **NZECA** | **Bathybiaster loripes** | **Kate Neill** |  | **2008** | **-74.73** | **167.01** | **Ross** |  |
| **NZECA234** |  | **NZECA** | **Bathybiaster loripes** | **Kate Neill** |  | **2008** | **-74.73** | **167.01** | **Ross** |  |
| **NZECA235** |  | **NZECA** | **Bathybiaster loripes** | **Kate Neill** |  | **1965** | **-66.47** | **162.76** | **Bal** | **371** |
| **NZECA236** |  | **NZECA** | **Bathybiaster loripes** | **Kate Neill** |  | **1965** | **-67.63** | **164.83** | **Bal** | **187** |
| **NZECA238** | **PsiNZECA238** | **NZECA** | **Psilaster acuminatus** | **Kate Neill** |  | **2007** | **-41.11** | **171.07** | **NZ** | **288** |
| **NZECA250** | **NZECA250** | **NZECA** | **Proserpinaster neozelanicus** | **Don Mcknight** |  | **1997** | **-49.90** | **173.88** | **NZ** |  |
| **NZECA251** | **NZECA251** | **NZECA** | **Proserpinaster neozelanicus** | **Don Mcknight** |  | **1997** | **-49.90** | **173.88** | **NZ** |  |
| **NZECA252** | **PsiNZECA252** | **NZECA** | **Psilaster acuminatus** | **Don Mcknight** |  | **1996** | **-36.92** | **176.29** | **NZ** | **400** |
| **NZECA261** | **PsiNZECA261** | **NZECA** | **Psilaster acuminatus** | **Kate Neill** |  | **2004** | **-39.41** | **179.87** | **NZ** | **2348** |
| **NZECA262** | **PsiNZECA262** | **NZECA** | **Psilaster acuminatus** | **Kate Neill** |  | **2009** | **-45.01** | **171.80** | **NZ** | **131** |
| **NZECA263** | **PsiNZECA263** | **NZECA** | **Psilaster acuminatus** | **Christopher L. Mah** |  | **2007** | **-44.28** | **178.53** | **NZ** | **1148** |
| **NZECA264** | **PsiNZECA264** | **NZECA** | **Psilaster acuminatus** | **Kate Neill** |  | **2007** | **-37.35** | **167.88** | **NZ** | **1051** |
| **NZECA265** | **PsiNZECA265** | **NZECA** | **Psilaster acuminatus** | **Kate Neill** |  | **2007** | **-44.49** | **177.14** | **NZ** | **1240** |
| **NZECA266** | **PsiNZECA266** | **NZECA** | **Psilaster acuminatus** | **Kate Neill** |  | **2007** | **-44.49** | **177.14** | **NZ** | **1240** |
| **NZECA267** | **PsiNZECA267** | **NZECA** | **Psilaster acuminatus** | **Kate Neill** |  | **2007** | **-44.49** | **177.14** | **NZ** | **1240** |
| **NZECA268** | **PsiNZECA268** | **NZECA** | **Psilaster acuminatus** | **Don Mcknight** |  | **2003** | **-49.33** | **176.55** | **NZ** | **1522** |
| **NZECA269** | **PsiNZECA269** | **NZECA** | **Psilaster acuminatus** | **Don Mcknight** |  | **2003** | **-49.33** | **176.55** | **NZ** | **1522** |
| **NZECA271** | **PsiNZECA271** | **NZECA** | **Psilaster acuminatus** | **Kate Neill** |  |  | **-36.11** | **173.26** | **NZ** | **848** |
| **NZECA272** | **PsiNZECA272** | **NZECA** | **Psilaster acuminatus** | **Kate Neill** |  |  | **-36.11** | **173.26** | **NZ** | **848** |
| **NZECA285** | **PsiNZECA285** | **NZECA** | **Psilaster charcoti** | **Kate Neill** |  | **2008** | **-76.77** | **167.84** | **Ross** | **724** |
| **NZECA286** | **PsiNZECA286** | **NZECA** | **Psilaster charcoti** | **Kate Neill** |  | **2008** | **-76.77** | **167.84** | **Ross** | **724** |
| **NZECA287** | **PsiNZECA287** | **NZECA** | **Psilaster charcoti** | **Kate Neill** |  | **2008** | **-76.77** | **167.84** | **Ross** | **724** |
| **NZECA288** | **PsiNZECA288** | **NZECA** | **Psilaster charcoti** | **Kate Neill** |  | **2008** | **-76.77** | **167.84** | **Ross** | **724** |
| **NZECA289** | **PsiNZECA289** | **NZECA** | **Psilaster charcoti** | **Kate Neill** |  | **2008** | **-76.77** | **167.84** | **Ross** | **724** |
| **NZECA290** | **PsiNZECA290** | **NZECA** | **Psilaster charcoti** | **Kate Neill** |  | **2008** | **-76.77** | **167.84** | **Ross** | **724** |
| **NZECA291** | **PsiNZECA291** | **NZECA** | **Psilaster charcoti** | **Kate Neill** |  | **2008** | **-76.77** | **167.84** | **Ross** | **724** |
| **NZECA292** | **PsiNZECA292** | **NZECA** | **Psilaster charcoti** | **Kate Neill** |  | **2008** | **-76.77** | **167.84** | **Ross** | **724** |
| **NZECA293** | **PsiNZECA293** | **NZECA** | **Psilaster charcoti** | **Kate Neill** |  | **2008** | **-76.77** | **167.84** | **Ross** | **724** |
| **NZECA294** | **PsiNZECA294** | **NZECA** | **Psilaster charcoti** | **Kate Neill** |  | **2008** | **-76.77** | **167.84** | **Ross** | **724** |
| **NZECA295** | **PsiNZECA295** | **NZECA** | **Psilaster charcoti** | **Kate Neill** |  | **2008** | **-76.59** | **176.82** | **Ross** | **369** |
| **NZECA296** | **PsiNZECA296** | **NZECA** | **Psilaster charcoti** | **Kate Neill** |  | **2008** | **-76.83** | **179.95** | **Ross** | **664** |
| **NZECA297** | **PsiNZECA297** | **NZECA** | **Psilaster charcoti** | **Kate Neill** |  | **2008** | **-76.83** | **179.95** | **Ross** | **664** |
| **NZECA298** | **PsiNZECA298** | **NZECA** | **Psilaster charcoti** | **Kate Neill** |  | **2008** | **-76.83** | **179.95** | **Ross** | **664** |
| **NZECA299** | **PsiNZECA299** | **NZECA** | **Psilaster charcoti** | **Kate Neill** |  | **2008** | **-76.83** | **179.95** | **Ross** | **664** |
| **NZECA300** | **PsiNZECA300** | **NZECA** | **Psilaster charcoti** | **Kate Neill** |  | **2008** | **-76.83** | **179.95** | **Ross** | **664** |
| **NZECA301** | **PsiNZECA301** | **NZECA** | **Psilaster charcoti** | **Kate Neill** |  | **2008** | **-76.83** | **179.95** | **Ross** | **664** |
| **NZECA302** | **PsiNZECA302** | **NZECA** | **Psilaster charcoti** | **Kate Neill** |  | **2008** | **-76.83** | **179.95** | **Ross** | **664** |
| **NZECA303** | **PsiNZECA303** | **NZECA** | **Psilaster charcoti** | **Kate Neill** |  | **2008** | **-74.72** | **167.01** | **Ross** | **916** |
| **NZECA304** | **PsiNZECA304** | **NZECA** | **Psilaster charcoti** | **Kate Neill** |  | **2008** | **-74.72** | **167.01** | **Ross** | **916** |
| **NZECA305** | **PsiNZECA305** | **NZECA** | **Psilaster charcoti** | **Kate Neill** |  | **2008** | **-74.72** | **167.01** | **Ross** | **916** |
| **NZECA306** | **PsiNZECA306** | **NZECA** | **Psilaster charcoti** | **Kate Neill** |  | **2008** | **-74.72** | **167.01** | **Ross** | **916** |
| **NZECA307** | **PsiNZECA307** | **NZECA** | **Psilaster charcoti** | **Kate Neill** |  | **2008** | **-75.63** | **169.85** | **Ross** | **525** |
| **NZECA308** | **PsiNZECA308** | **NZECA** | **Psilaster charcoti** | **Kate Neill** |  | **2008** | **-74.74** | **167.06** | **Ross** | **863** |
| **NZECA309** | **PsiNZECA309** | **NZECA** | **Psilaster charcoti** | **Kate Neill** |  | **2008** | **-74.74** | **167.06** | **Ross** | **863** |
| **NZECA310** | **PsiNZECA310** | **NZECA** | **Psilaster charcoti** | **Kate Neill** |  | **2008** | **-74.74** | **167.06** | **Ross** | **863** |
| **NZECA311** | **PsiNZECA311** | **NZECA** | **Psilaster charcoti** | **Kate Neill** |  | **2008** | **-74.74** | **167.06** | **Ross** | **863** |
| **NZECA312** | **PsiNZECA312** | **NZECA** | **Psilaster charcoti** | **Kate Neill** |  | **2008** | **-74.74** | **167.06** | **Ross** | **863** |
| **NZECA313** | **PsiNZECA313** | **NZECA** | **Psilaster charcoti** | **Kate Neill** |  | **2008** | **-74.72** | **167.01** | **Ross** | **916** |
| **NZECA314** | **PsiNZECA314** | **NZECA** | **Psilaster charcoti** | **Kate Neill** |  | **2008** | **-74.72** | **167.01** | **Ross** | **916** |
| **NZECA315** | **PsiNZECA315** | **NZECA** | **Psilaster charcoti** | **Kate Neill** |  | **2008** | **-74.72** | **167.01** | **Ross** | **916** |
| **NZECA316** | **PsiNZECA316** | **NZECA** | **Psilaster charcoti** | **Kate Neill** |  | **2008** | **-74.72** | **167.01** | **Ross** | **916** |
| **NZECA317** | **NZECA317** | **NZECA** | **Psilaster charcoti** | **Kate Neill** |  | **2008** | **-74.72** | **167.01** | **Ross** | **916** |
| **NZECA335** | **PsiNZECA335** | **NZECA** | **Psilaster acuminatus** | **Kate Neill** |  | **1971** | **-52.49** | **169.75** | **NZ** | **155** |
| **NZECA336** | **PsiNZECA336** | **NZECA** | **Psilaster acuminatus** | **Kate Neill** |  | **1978** | **-53.26** | **169.51** | **NZ** | **480** |
| **NZECA338** | **PsiNZECA338** | **NZECA** | **Psilaster acuminatus** | **Kate Neill** |  | **1978** | **-53.26** | **169.51** | **NZ** | **480** |
| **NZECA339** | **PsiNZECA339** | **NZECA** | **Psilaster acuminatus** | **Kate Neill** |  | **1971** | **-51.93** | **170.23** | **NZ** | **200** |
| **NZECA340** | **PsiNZECA340** | **NZECA** | **Psilaster acuminatus** | **Kate Neill** |  | **1971** | **-51.93** | **170.23** | **NZ** | **200** |
| **NZECA341** | **PsiNZECA341** | **NZECA** | **Psilaster charcoti** | **Kate Neill** |  | **2008** | **-66.55** | **-177.67** | **Ross** | **1744** |
| **NZECA342** | **PsiNZECA342** | **NZECA** | **Psilaster acuminatus** | **Kate Neill** |  | **2002** | **-53.24** | **168.86** | **NZ** | **698** |
| **NZECA343** | **PsiNZECA343** | **NZECA** | **Psilaster acuminatus** | **Kate Neill** |  | **1978** | **-51.64** | **165.40** | **NZ** | **2460** |
| **NZECA344** | **PsiNZECA344** | **NZECA** | **Psilaster acuminatus** | **Kate Neill** |  | **1978** | **-52.78** | **172.90** | **NZ** | **494** |
| **NZECA345** | **PsiNZECA345** | **NZECA** | **Psilaster acuminatus** | **Kate Neill** |  | **1978** | **-52.78** | **172.90** | **NZ** | **494** |
| **NZECA349** | **PsiNZECA349** | **NZECA** | **Psilaster charcoti** | **Kate Neill** |  | **1965** | **-72.17** | **170.80** | **Ross** | **353** |
| **NZECA350** | **PsiNZECA350** | **NZECA** | **Psilaster charcoti** | **Kate Neill** |  | **1965** | **-72.17** | **170.80** | **Ross** | **353** |
| **NZECA351** | **PsiNZECA351** | **NZECA** | **Psilaster charcoti** | **Kate Neill** |  | **1965** | **-72.17** | **170.80** | **Ross** | **353** |
| **NZECA353** | **PsiNZECA353** | **NZECA** | **Psilaster charcoti** | **Kate Neill** |  | **1959** | **-73.53** | **171.37** | **Ross** | **550** |
| **NZECA354** | **PsiNZECA354** | **NZECA** | **Psilaster charcoti** | **Kate Neill** |  | **1959** | **-73.53** | **171.37** | **Ross** | **550** |
| **NZECA356** | **PsiNZECA356** | **NZECA** | **Psilaster charcoti** | **Kate Neill** |  | **2008** | **-75.62** | **169.80** | **Ross** | **520** |
| **NZECA357** | **PsiNZECA357** | **NZECA** | **Psilaster charcoti** | **Kate Neill** |  | **2008** | **-75.62** | **167.32** | **Ross** | **480** |
| **NZECA358** | **PsiNZECA358** | **NZECA** | **Psilaster charcoti** | **Kate Neill** |  | **2008** | **-76.20** | **167.24** | **Ross** | **451** |
| **NZECA359** | **PsiNZECA359** | **NZECA** | **Psilaster charcoti** | **Kate Neill** |  | **2008** | **-76.19** | **176.29** | **Ross** | **447** |
| **NZECA361** | **PsiNZECA361** | **NZECA** | **Psilaster charcoti** | **Kate Neill** |  | **2008** | **-76.61** | **176.81** | **Ross** | **360** |
| **NZECA362** | **PsiNZECA362** | **NZECA** | **Psilaster charcoti** | **Kate Neill** |  | **2008** | **-76.61** | **176.81** | **Ross** | **360** |
| **NZECA363** | **PsiNZECA363** | **NZECA** | **Psilaster charcoti** | **Kate Neill** |  | **2008** | **-76.61** | **176.81** | **Ross** | **360** |
| **NZECA367** | **PsiNZECA367** | **NZECA** | **Psilaster charcoti** | **Kate Neill** |  | **2008** | **-76.83** | **179.95** | **Ross** | **664** |
| **NZECA368** | **PsiNZECA368** | **NZECA** | **Psilaster charcoti** | **Kate Neill** |  | **2008** | **-73.62** | **167.32** | **Ross** | **480** |
| **NZECA369** | **PsiNZECA369** | **NZECA** | **Psilaster charcoti** | **Kate Neill** |  | **2008** | **-73.62** | **167.32** | **Ross** | **480** |
| **NZECA370** | **PsiNZECA370** | **NZECA** | **Psilaster charcoti** | **Kate Neill** |  | **2008** | **-73.62** | **167.32** | **Ross** | **480** |
| **NZECA371** | **PsiNZECA371** | **NZECA** | **Psilaster charcoti** | **Kate Neill** |  | **1959** | **-75.28** | **172.33** | **Ross** | **520** |
| **NZECA373** | **PsiNZECA373** | **NZECA** | **Psilaster charcoti** | **Kate Neill** |  | **2008** | **-73.12** | **174.32** | **Ross** | **321** |
| **NZECA374** | **PsiNZECA374** | **NZECA** | **Psilaster charcoti** | **Kate Neill** |  | **2008** | **-74.59** | **170.27** | **Ross** | **283** |
| **NZECA375** | **PsiNZECA375** | **NZECA** | **Psilaster charcoti** | **Kate Neill** |  | **2008** | **-72.58** | **175.31** | **Ross** | **467** |
| **NZECA378** | **PsiNZECA378** | **NZECA** | **Psilaster charcoti** |  |  |  |  |  |  |  |
| **NZECA379** | **PsiNZECA379** | **NZECA** | **Psilaster charcoti** |  |  |  |  |  |  |  |
| **NZECA642** | **PsiNZECA642** | **NZECA** | **Psilaster charcoti** |  |  |  | **-65.30** | **-53.38** | **AntPen** | **2087** |
| **NZECA643** | **PsiNZECA643** | **NZECA** | **Psilaster charcoti** |  |  | **2002** | **-58.73** | **-25.17** | **SSand** | **753** |
| **NZECA644** | **PsiNZECA644** | **NZECA** | **Psilaster charcoti** |  |  | **2003** | **-61.04** | **-54.50** | **TipPen** | **134** |
| **NZECA648** | **PsiNZECA648** | **NZECA** | **Psilaster charcoti** |  |  |  |  |  |  |  |
| **NZECA650** | **PsiNZECA650** | **NZECA** | **Psilaster charcoti** |  |  | **2009** | **-61.05** | **-46.82** | **Sork** |  |
| **NZECA651** | **PsiNZECA651** | **NZECA** | **Psilaster charcoti** |  |  |  |  |  |  |  |
| **NZECA652** | **PsiNZECA652** | **NZECA** | **Psilaster charcoti** |  |  |  |  |  |  |  |
| **NZECA683** | **PsiNZECA683** | **NZECA** | **Psilaster acuminatus** | **Owen Anderson** |  | **1983** | **-34.93** | **151.25** | **Aus** | **1152** |
| **ODTNB042-15** |  | **JR287SG** | **Undetermined** | **Camille Moreau** |  | **2013** |  |  | **SG** | **245** |
| **ODTNB047-15** | **Lys022** | **JR230** | **Undet (cf lysasterias)+brood** | **Camille Moreau** |  | **2009** | **-67.72** | **-70.06** | **Belli** | **463.95** |
| **ODTNB053-15** | **Dip078** | **JR144** | **Undet (cf diplasterias)** | **Camille Moreau** |  | **2006** | **-60.99** | **-46.83** | **Sork** | **507** |
| **ODTNB059-15** | **Psi187** | **JR144** | **Psilaster charcoti** | **Camille Moreau** |  | **2006** | **-62.92** | **-61.00** | **Sshet** | **199** |
| **ODTNB060-15** | **Dip055** | **JR144** | **Undet (cf diplasterias)** | **Camille Moreau** |  | **2006** | **-62.92** | **-61.00** | **Sshet** | **199** |
| **ODTNB086-15** | **Bat234** | **JR144** | **Bathybiaster loripes** | **Camille Moreau** |  | **2006** | **-62.53** | **-61.82** | **Sshet** | **190** |
| **ODTNB091-15** | **Bat229** | **ARGOS** | **Bathybiaster loripes** | **Camille Moreau** |  | **2009** | **-58.75** | **-25.24** | **Ssand** | **1428** |
| **SOA032-12** |  |  |  | **Christopher L. Mah** |  |  |  |  |  |  |
| **SOA488-12** |  | **POKER II** |  | **Christopher L. Mah** |  | **2011** |  |  | **Ker** | **124** |
| **SOA572-12** | **Not180** | **JR144** | **Notasterias bongraini** | **Christopher L. Mah** |  | **2006** | **-61.58** | **-55.26** | **Eleph** | **987** |
| **SOA574-12** | **Psi107** | **JR144** | **Psilaster charcoti** | **Christopher L. Mah** |  | **2006** | **-59.51** | **-27.30** | **Ssand** | **1043** |
| **SOA575-12** | **Psi108** | **JR144** | **Psilaster charcoti** | **Christopher L. Mah** |  | **2006** | **-59.51** | **-27.30** | **Ssand** | **1043** |
| **SOA576-12** | **Bat070** | **JR144** | **Bathybiaster loripes** | **Christopher L. Mah** |  | **2006** | **-61.04** | **-46.96** | **Sork** | **1630** |
| **SOA577-12** | **Psi110** | **JR144** | **Psilaster charcoti** | **Christopher L. Mah** |  | **2006** | **-59.47** | **-27.28** | **Ssand** | **290** |
| **SOA578-12** | **Psi111** | **JR144** | **Psilaster charcoti** | **Christopher L. Mah** |  | **2006** | **-59.47** | **-27.28** | **Ssand** | **290** |
| **SOA579-12** | **Psi109** | **JR144** | **Bathybiaster loripes** | **Christopher L. Mah** |  | **2006** | **-59.47** | **-27.28** | **Ssand** | **290** |
| **SOA580-12** | **Psi112** | **JR144** | **Psilaster charcoti** | **Christopher L. Mah** |  | **2006** | **-59.47** | **-27.28** | **Ssand** | **290** |
| **SOA602-12** | **Lys112** | **JR144** | **Diplasterias sp.** | **Christopher L. Mah** |  | **2006** | **-61.58** | **-55.26** | **Eleph** | **987** |
| **SOA610-12** | **Dip192** | **JR144** | **Diplasterias brucei** | **Christopher L. Mah** |  | **2006** | **-54.31** | **-56.68** | **Mag** | **201** |
| **SOA613-12** | **Dip190** | **JR179** | **Pedicellaster hypernotius** | **Christopher L. Mah** |  | **2008** | **-68.53** | **-76.20** | **Belli** | **463.12** |
| **SOA637-12** | **Bat072** | **JR144** | **Bathybiaster loripes** | **Christopher L. Mah** |  | **2006** | **-59.51** | **-27.30** | **Ssand** | **1043** |
| **SOA646-12** | **Dip189** | **JR144** | **Diplasterias cf. brucei** | **Christopher L. Mah** |  | **2006** | **-59.47** | **-27.28** | **Ssand** | **290** |
| **SOA647-12** | **Dip194** | **JR144** | **Diplasterias cf. brucei** | **Christopher L. Mah** |  | **2006** | **-59.47** | **-27.28** | **Ssand** | **290** |
| **SOA648-12** | **Dip193** | **JR144** | **Diplasterias cf. brucei** | **Christopher L. Mah** |  | **2006** | **-59.47** | **-27.28** | **Ssand** | **290** |
| **SOA652-12** | **Psi116** | **JR144** | **Psilaster charcoti** | **Christopher L. Mah** |  | **2006** | **-59.48** | **-27.28** | **Ssand** | **550** |
| **SOA654-12** |  | **JR144** | **Misidentified** | **Christopher L. Mah** |  | **2006** | **-53.63** | **-40.90** | **Shag** | **212** |
| **SOA655-12** |  | **JR144** | **Misidentified** | **Christopher L. Mah** |  | **2006** | **-53.63** | **-40.90** | **Shag** | **212** |
| **SOA659-12** | **Psi113** | **JR144** | **Psilaster charcoti** | **Christopher L. Mah** |  | **2006** | **-61.58** | **-55.26** | **Eleph** | **987** |
| **SOA660-12** | **Psi114** | **JR144** | **Psilaster charcoti** | **Christopher L. Mah** |  | **2006** | **-61.58** | **-55.26** | **Eleph** | **987** |
| **SOA669-12** | **Not184** | **JR144** | **Notasterias bongraini** | **Christopher L. Mah** |  | **2006** | **-60.99** | **-46.83** | **Sork** | **507** |
| **SOA670-12** | **Not185** | **JR144** | **Notasterias bongraini** | **Christopher L. Mah** |  | **2006** | **-60.99** | **-46.83** | **Sork** | **507** |
| **SOA671-12** | **Not186** | **JR144** | **Notasterias bongraini** | **Christopher L. Mah** |  | **2006** | **-60.99** | **-46.83** | **Sork** | **507** |
| **SOA672-12** | **Not187** | **JR144** | **Notasterias bongraini** | **Christopher L. Mah** |  | **2006** | **-60.99** | **-46.83** | **Sork** | **507** |
| **SOA673-12** | **Not188** | **JR144** | **Notasterias bongraini** | **Christopher L. Mah** |  | **2006** | **-60.99** | **-46.83** | **Sork** | **507** |
| **SOA678-12** | **Dip195** | **JR144** | **Diplasterias brandti** | **Christopher L. Mah** |  | **2006** | **-60.99** | **-46.83** | **Sork** | **507** |
| **SOA679-12** | **Dip319** | **JR144** | **Diplasterias brandti** | **Christopher L. Mah** |  | **2006** | **-60.99** | **-46.83** | **Sork** | **507** |
| **SOA680-12** | **Bat233** | **JR144** | **Bathybiaster loripes** | **Christopher L. Mah** |  | **2006** | **-59.48** | **-27.28** | **Ssand** | **550** |
| **SOA682-12** | **Psi243** |  |  | **Christopher L. Mah** |  |  |  |  |  |  |
| **SOA683-12** | **Bat230** | **JR144** | **Bathybiaster loripes** | **Christopher L. Mah** |  | **2006** | **-59.48** | **-27.28** | **Ssand** | **550** |
| **SOA684-12** | **Psi244** |  |  | **Christopher L. Mah** |  |  |  |  |  |  |
| **SOA685-12** | **Bat231** | **JR144** | **Bathybiaster loripes** | **Christopher L. Mah** |  | **2006** | **-59.48** | **-27.28** | **Ssand** | **550** |
| **SOA703-12** | **Lys117** | **JR144** | **Lysasterias perrieri** | **Christopher L. Mah** |  | **2006** | **-61.58** | **-55.26** | **Eleph** | **987** |
| **SOA704-12** | **Lys114** | **JR144** | **Lysasterias perrieri** | **Christopher L. Mah** |  | **2006** | **-61.58** | **-55.26** | **Eleph** | **987** |
| **SOA705-12** | **Lys115** | **JR144** | **Lysasterias perrieri** | **Christopher L. Mah** |  | **2006** | **-61.58** | **-55.26** | **Eleph** | **987** |
| **SOA706-12** | **Lys113** | **JR144** | **Lysasterias perrieri** | **Christopher L. Mah** |  | **2006** | **-61.58** | **-55.26** | **Eleph** | **987** |
| **SOA707-12** | **Lys116** | **JR144** | **Lysasterias perrieri** | **Christopher L. Mah** |  | **2006** | **-61.58** | **-55.26** | **Eleph** | **987** |
| **SOA708-12** | **Not182** | **JR144** | **Notasterias armata** | **Christopher L. Mah** |  | **2006** | **-61.39** | **-55.19** | **Eleph** | **483** |
| **SOA709-12** | **Not183** | **JR144** | **Notasterias armata** | **Christopher L. Mah** |  | **2006** | **-61.39** | **-55.19** | **Eleph** | **483** |
| **SOA749-12** | **Bat071** | **JR179** | **Bathybiaster loripes** | **Christopher L. Mah** |  | **2008** | **-73.87** | **-106.30** | **Amund** | **1094.02** |
| **SORK002-17** | **Bat215** | **JR15005** | **Bathybiaster loripes** | **Camille Moreau** |  | **2016** | **-60.35** | **-46.68** | **Sork** | **465** |
| **SORK004-17** | **Lys018** | **JR15005** | **Lysasterias sp** | **Camille Moreau** |  | **2016** | **-60.35** | **-46.68** | **Sork** | **458** |
| **SORK018-17** | **Bat212** | **JR15005** | **Bathybiaster loripes** | **Camille Moreau** |  | **2016** | **-60.32** | **-46.77** | **Sork** | **722** |
| **SORK019-17** | **Bat214** | **JR15005** | **Bathybiaster loripes** | **Camille Moreau** |  | **2016** | **-60.22** | **-46.69** | **Sork** | **783** |
| **SORK028-17** | **Not009** | **JR15005** | **Notasterias sp** | **Camille Moreau** |  | **2016** | **-60.28** | **-46.87** | **Sork** | **484** |
| **SORK040-17** | **Lys317** | **JR15005** |  | **Camille Moreau** |  | **2016** | **-60.27** | **-46.88** | **Sork** | **459** |
| **SORK052-17** | **Dip098** | **JR15005** | **Diplasterias** | **Camille Moreau** |  | **2016** | **-60.50** | **-44.52** | **Sork** | **551** |
| **SORK055-17** | **Lys019** | **JR15005** | **Lysasterias sp** | **Camille Moreau** |  | **2016** | **-60.50** | **-44.52** | **Sork** | **551** |
| **SORK058-17** | **Not076** | **JR15005** | **Notasterias sp** | **Camille Moreau** |  | **2016** | **-60.46** | **-44.70** | **Sork** | **1028** |
| **SORK132-17** | **Lys013** | **JR15005** | **Lysasterias sp** | **Camille Moreau** |  | **2016** | **-60.68** | **-42.52** | **Sork** | **493** |
| **SORK133-17** | **Lys012** | **JR15005** | **Lysasterias sp** | **Camille Moreau** |  | **2016** | **-60.68** | **-42.52** | **Sork** | **493** |
| **SORK136-17** | **Bat213** | **JR15005** | **Bathybiaster loripes** | **Camille Moreau** |  | **2016** | **-60.66** | **-43.10** | **Sork** | **2133** |
| **SORK148-17[** | **Not029** | **JR15005** | **Notasterias sp** | **Camille Moreau** |  | **2016** | **-60.56** | **-41.03** | **Sork** | **617** |
| **SORK149-17** | **Lys014** | **JR15005** | **Lysasterias sp** | **Camille Moreau** |  | **2016** | **-60.56** | **-41.03** | **Sork** | **617** |
| **SORK176-17** | **Dip374** | **JR15005** | **Diplasterias** | **Camille Moreau** |  | **2016** | **-62.33** | **-44.54** | **Sork** | **969** |
| **SORK182-17** | **Not050** | **JR15005** | **Notasterias sp** | **Camille Moreau** |  | **2016** | **-62.33** | **-44.54** | **Sork** | **969** |
| **SORK185-17** | **Dip101** | **JR15005** |  | **Camille Moreau** |  | **2016** | **-62.33** | **-44.54** | **Sork** | **969** |
| **SWEMA104-15** |  |  |  |  |  |  |  |  |  |  |
| **SWEMA163-15** |  |  |  |  |  |  |  |  |  |  |
| **TCTNB053-15** | **Not015** | **XXIX** | **Notasterias armata** | **Camille Moreau** |  | **2014** |  |  | **Ross** | **242** |
| **TCTNB058-15** | **Psi226** | **XXVIII** | **Psilaster charcoti** | **Camille Moreau** |  | **2013** |  |  | **Ross** | **90** |
| **TCTNB060-15** | **Lys009** | **XXVIII** | **Lysasterias sp** | **Camille Moreau** |  | **2013** |  |  | **Ross** | **35** |
| **TCTNB065-15** | **Lys007** | **XXVIII** | **Lysasterias sp** | **Camille Moreau** |  | **2013** |  |  | **Ross** | **150** |
| **TCTNB067-15** | **Not018** | **XXVIII** | **Notasterias armata** | **Camille Moreau** |  | **2013** |  |  | **Ross** | **140** |
| **TCTNB068-15** | **Not014** | **XXIX** | **Notasterias armata** | **Camille Moreau** |  | **2014** |  |  | **Ross** | **242** |
| **TCTNB069-15** | **Dip032** | **XXVIII** | **Diplasterias brucei** | **Camille Moreau** |  | **2013** |  |  | **Ross** | **90** |
| **TCTNB071-15** | **Not016** | **XXIX** | **Notasterias armata** | **Camille Moreau** |  | **2014** |  |  | **Ross** | **242** |
| **TCTNB072-15** | **Not022** | **XXVIII** | **Notasterias armata** | **Camille Moreau** |  | **2013** |  |  | **Ross** | **150** |
| **TCTNB073-15** | **Psi225** | **XXVIII** | **Psilaster charcoti** | **Camille Moreau** |  | **2013** |  |  | **Ross** | **293** |
| **TCTNB075-15** | **Not021** | **XXVIII** | **Notasterias armata** | **Camille Moreau** |  | **2013** |  |  | **Ross** | **150** |
| **TCTNB076-15** | **Dip002** | **XXIX** | **Diplasterias brucei** | **Camille Moreau** |  | **2014** |  |  | **Ross** | **130** |
| **TCTNB083-15** | **Psi223** | **XXIX** | **Psilaster charcoti** | **Camille Moreau** |  | **2014** |  |  | **Ross** | **100** |
| **TCTNB084-15** | **Psi224** | **XXIX** | **Psilaster charcoti** | **Camille Moreau** |  | **2014** |  |  | **Ross** | **100** |
| **TCTNB085-15** | **Dip038** | **XXVIII** | **Diplasterias brucei** | **Camille Moreau** |  |  |  |  | **Ross** | **25** |
| **TCTNB086-15** | **Dip039** | **XXVIII** | **Diplasterias brucei** | **Camille Moreau** |  |  |  |  | **Ross** | **25** |
| **TCTNB087-15** | **Dip040** | **XXVIII** | **Diplasterias brucei** | **Camille Moreau** |  |  |  |  | **Ross** | **25** |
| **TCTNB088-15** | **Dip041** | **XXVIII** | **Diplasterias brucei** | **Camille Moreau** |  |  |  |  | **Ross** | **25** |
| **TCTNB093-15** | **Not023** | **XXVIII** | **Notasterias armata** | **Camille Moreau** |  | **2013** |  |  | **Ross** | **208** |
| **TCTNB094-15** | **Not020** | **XXVIII** | **Notasterias armata** | **Camille Moreau** |  | **2013** |  |  | **Ross** | **150** |
| **TCTNB095-15** | **Dip001** | **XVII** | **Diplasterias brucei** | **Camille Moreau** |  | **2001** |  |  | **Ross** | **50** |
| **TNBA007-15** | **Not019** | **XXVIII** | **Notasterias armata** | **Camille Moreau** |  |  |  |  | **Ross** |  |
| **TNBA008-15** | **Not024** | **XXVIII** | **Notasterias armata** | **Camille Moreau** |  |  |  |  | **Ross** |  |
| **TNBA009-15** | **Not017** | **XXVIII** | **Notasterias armata** | **Camille Moreau** |  |  |  |  | **Ross** |  |
| **TNBA011-15** | **Lys005** | **XXIX** | **Lysasterias sp** | **Camille Moreau** |  |  |  |  | **Ross** |  |
| **TNBA012-15** | **Lys006** | **XXVIII** | **Lysasterias sp** | **Camille Moreau** |  |  |  |  | **Ross** |  |
| **TNBA018-15** | **Dip033** | **XXVIII** | **Diplasterias brucei** | **Camille Moreau** |  |  |  |  | **Ross** |  |
| **TNBA033-15** | **Bat227** | **ANDEEP-III** | **Bathybiaster loripes** | **Camille Moreau** |  | **2005** | **-69.38** | **-5.25** | **WS** | **1822** |
| **TNBA034-15** | **Bat271** | **ANDEEP-III** | **Bathybiaster loripes** | **Camille Moreau** |  | **2005** | **-69.38** | **-5.25** | **WS** | **1822** |
| **TNBA035-15** | **Bat272** | **ANDEEP-III** | **Bathybiaster loripes** | **Camille Moreau** |  | **2005** | **-69.38** | **-5.25** | **WS** | **1822** |
| **TNBA037-15** | **Bat248** | **ANDEEP-III** | **Bathybiaster loripes** | **Camille Moreau** |  | **2005** | **-63.35** | **-64.61** | **Sshet** | **2124** |
| **TNBA038-15** | **Bat249** | **ANDEEP-III** | **Bathybiaster loripes** | **Camille Moreau** |  | **2005** | **-63.35** | **-64.61** | **Sshet** | **2124** |
| **TNBA039-15** | **Bat242** | **JR230** | **Bathybiaster loripes** | **Camille Moreau** |  | **2009** | **-67.92** | **-68.53** | **Belli** | **568.77** |
| **TNBA040-15** | **Bat244** | **JR230** | **Bathybiaster loripes** | **Camille Moreau** |  | **2009** | **-67.91** | **-68.53** | **Belli** | **543.95** |
| **TNBA041-15** | **Bat246** | **JR230** | **Bathybiaster loripes** | **Camille Moreau** |  | **2009** | **-67.99** | **-68.60** | **Belli** | **505.1** |
| **TNBA042-15** | **Bat245** | **JR230** | **Bathybiaster loripes** | **Camille Moreau** |  | **2009** | **-67.91** | **-68.53** | **Belli** | **543.95** |
| **TNBA043-15** | **Bat241** | **JR230** | **Bathybiaster loripes** | **Camille Moreau** |  | **2009** | **-67.17** | **-69.45** | **Belli** | **454.41** |
| **TNBA044-15** | **Bat243** | **JR230** | **Bathybiaster loripes** | **Camille Moreau** |  | **2009** | **-67.08** | **-69.40** | **Belli** | **473.23** |
| **TNBA045-15** | **Bat247** | **JR230** | **Bathybiaster loripes** | **Camille Moreau** |  | **2009** | **-67.83** | **-70.84** | **Belli** | **588.98** |
| **TNBA069-15** | **Not026** | **ANDEEP-III** | **Notasterias sp** | **Camille Moreau** |  | **2005** | **-62.55** | **-64.54** | **Sshet** | **3799** |
| **TNBA073-15** | **Bat232** | **JR144** | **Bathybiaster loripes** | **Camille Moreau** |  | **2006** | **-61.33** | **-55.20** | **Eleph** | **201** |
| **TNBA074-15** | **Bat235** | **JR179** | **Bathybiaster loripes** | **Camille Moreau** |  | **2008** | **-74.48** | **-104.24** | **Amund** | **1203.06** |
| **TNBA075-15** | **Bat236** | **JR179** | **Bathybiaster loripes** | **Camille Moreau** |  | **2008** | **-74.48** | **-104.24** | **Amund** | **1203.06** |
| **TNBA076-15** | **Bat237** | **JR179** | **Bathybiaster loripes** | **Camille Moreau** |  | **2008** | **-74.48** | **-104.24** | **Amund** | **1203.06** |
| **TNBA077-15** | **Bat238** | **JR179** | **Bathybiaster loripes** | **Camille Moreau** |  | **2008** | **-74.48** | **-104.24** | **Amund** | **1203.06** |
| **TNBA078-15** | **Bat239** | **JR179** | **Bathybiaster loripes** | **Camille Moreau** |  | **2008** | **-74.48** | **-104.24** | **Amund** | **1203.06** |
| **TNBA079-15** | **Psi208** | **JR262** | **Psilaster charcoti** | **Camille Moreau** |  | **2011** | **-54.90** | **-36.53** | **SG** | **250** |
| **TNBA080-15** | **Bat250** | **JR262** | **Bathybiaster loripes** | **Camille Moreau** |  | **2011** | **-54.90** | **-36.53** | **SG** | **250** |
| **TNBA081-15** | **Bat251** | **JR262** | **Bathybiaster loripes** | **Camille Moreau** |  | **2011** | **-54.90** | **-36.53** | **SG** | **250** |
| **TNBA082-15** | **Bat252** | **JR262** | **Bathybiaster loripes** | **Camille Moreau** |  | **2011** | **-54.90** | **-36.53** | **SG** | **250** |
| **TNBA083-15** | **Bat253** | **JR262** | **Bathybiaster loripes** | **Camille Moreau** |  | **2011** | **-54.90** | **-36.53** | **SG** | **250** |
| **TNBA084-15** | **Bat240** | **JR179** | **Bathybiaster loripes** | **Camille Moreau** |  | **2008** | **-74.48** | **-104.24** | **Amund** | **1203.06** |
| **TNBA085-15** | **Bat254** | **JR308** | **Bathybiaster loripes** | **Camille Moreau** |  | **2014** | **-67.75** | **-68.16** | **Belli** | **480** |
| **TNBA088-15** | **Bat222** | **ANDEEP-III** | **Bathybiaster loripes** | **Camille Moreau** |  | **2005** | **-63.35** | **-64.61** | **Sshet** | **2124** |
| **Psi253** | **Psi253** | **JR275** | **Psilaster charcoti** | **Camille Moreau** |  | **2012** | **-75246.00** | **-2901895.00** | **WS** | **393** |
| **Not506** | **Not506** | **ACE_2017** | **Notasterias sp** | **Camille Moreau** |  | **2017** | **-73.20** | **-127.27** | **Amund** | **340** |
| **Lys349** | **Lys349** | **ACE_2017** | **Lysasterias sp** | **Camille Moreau** |  | **2017** | **-73.16** | **-126.98** | **Amund** | **270** |
| **Und053** | **Und053** | **JR179** | **Pedicellaster hypernotius** | **Christopher L. Mah** |  | **2008** | **-71.15** | **-110.01** | **Amund** | **1484.69** |
| **Psi201** | **Psi201** | **JR179** | **Psilaster charcoti** | **Camille Moreau** |  | **2008** | **-73.98** | **-107.41** | **Amund** | **535** |
| **Dip091** | **Dip091** | **JR230** | **Diplasterias brucei** | **Camille Moreau** |  | **2009** | **-66.26** | **-70.65** | **Belli** | **509.61** |
| **Und015** | **Und015** | **JR230** | **juv.** | **Camille Moreau** |  | **2009** | **-66.25** | **-70.64** | **Belli** | **507.88** |
| **Dip093** | **Dip092** | **JR230** | **Diplasterias brucei** | **Camille Moreau** |  | **2009** | **-66.19** | **-70.53** | **Belli** | **502.05** |
| **Lys010** | **Lys010** | **JR230** | **Lysasterias sp** | **Camille Moreau** |  | **2009** | **-66.26** | **-70.42** | **Belli** | **514** |
| **Psi207** | **Psi207** | **JR230** | **Psilaster charcoti** | **Camille Moreau** |  | **2009** | **-67.76** | **-70.20** | **Belli** | **610.71** |
| **Lys011** | **Lys011** | **JR230** | **Lysasterias sp** | **Camille Moreau** |  | **2009** | **-67.53** | **-70.20** | **Belli** | **530** |
| **Psi203** | **Psi203** | **JR230** | **Psilaster charcoti** | **Camille Moreau** |  | **2009** | **-67.11** | **-69.63** | **Belli** | **549.47** |
| **Psi204** | **Psi204** | **JR230** | **Psilaster charcoti** | **Camille Moreau** |  | **2009** | **-67.11** | **-69.61** | **Belli** | **528.85** |
| **Psi221** | **Psi221** | **JR308** | **Psilaster charcoti** | **Camille Moreau** |  | **2014** | **-67.80** | **-68.62** | **Belli** | **450** |
| **Psi220** | **Psi220** | **JR308** | **Psilaster charcoti** | **Camille Moreau** |  | **2014** | **-67.83** | **-68.56** | **Belli** | **550** |
| **Psi222** | **Psi222** | **JR308** | **Psilaster charcoti** | **Camille Moreau** |  | **2014** | **-67.83** | **-68.56** | **Belli** | **550** |
| **Psi202** | **Psi202** | **JR230** | **Psilaster charcoti** | **Camille Moreau** |  | **2009** | **-67.92** | **-68.53** | **Belli** | **568.77** |
| **Psi205** | **Psi205** | **JR230** | **Psilaster charcoti** | **Camille Moreau** |  | **2009** | **-67.91** | **-68.53** | **Belli** | **543.95** |
| **Psi206** | **Psi206** | **JR230** | **Psilaster charcoti** | **Camille Moreau** |  | **2009** | **-67.91** | **-68.53** | **Belli** | **543.95** |
| **Psi217** | **Psi217** | **JR308** | **Psilaster charcoti** | **Camille Moreau** |  | **2014** | **-67.79** | **-68.51** | **Belli** | **540** |
| **Psi218** | **Psi218** | **JR308** | **Psilaster charcoti** | **Camille Moreau** |  | **2014** | **-67.79** | **-68.51** | **Belli** | **540** |
| **Psi219** | **Psi219** | **JR308** | **Psilaster charcoti** | **Camille Moreau** |  | **2014** | **-67.79** | **-68.51** | **Belli** | **540** |
| **Psi215** | **Psi215** | **JR308** | **Psilaster charcoti** | **Camille Moreau** |  | **2014** | **-67.78** | **-68.50** | **Belli** | **520** |
| **Psi216** | **Psi216** | **JR308** | **Psilaster charcoti** | **Camille Moreau** |  | **2014** | **-67.78** | **-68.50** | **Belli** | **520** |
| **Dip094** | **Dip094** | **JR308** | **undet** | **Camille Moreau** |  | **2014** | **-67.76** | **-68.36** | **Belli** | **225** |
| **Not027** | **Not027** | **JR308** | **Notasterias sp** | **Camille Moreau** |  | **2014** | **-67.76** | **-68.36** | **Belli** | **180** |
| **Psi213** | **Psi213** | **JR308** | **Psilaster charcoti** | **Camille Moreau** |  | **2014** | **-67.75** | **-68.17** | **Belli** | **455** |
| **Psi214** | **Psi214** | **JR308** | **Psilaster charcoti** | **Camille Moreau** |  | **2014** | **-67.75** | **-68.16** | **Belli** | **480** |
| **Bat276** | **Bat276** | **ANDEEP-III** | **Bathybiaster loripes** | **Camille Moreau** |  | **2005** | **-63.35** | **-64.61** | **Sshet** | **2124** |
| **Psi200** | **Psi200** | **ANDEEP-III** | **Psilaster charcoti** | **Camille Moreau** |  | **2005** | **-63.35** | **-64.61** | **Sshet** | **2124** |
| **Lys001** | **Lys001** | **ANDEEP-III** | **Lysasterias sp** | **Camille Moreau** |  | **2005** | **-62.55** | **-64.54** | **Sshet** | **3799** |
| **Lys003** | **Lys003** | **ANDEEP-III** | **Lysasterias sp** | **Camille Moreau** |  | **2005** | **-62.55** | **-64.54** | **Sshet** | **3799** |
| **Bat266** | **Bat266** | **JR144** | **Bathybiaster loripes** | **Camille Moreau** |  | **2006** | **-62.53** | **-61.82** | **Sshet** | **190** |
| **Bat269** | **Bat269** | **JR144** | **Bathybiaster loripes** | **Camille Moreau** |  | **2006** | **-62.53** | **-61.82** | **Sshet** | **190** |
| **Bat270** | **Bat270** | **JR144** | **Bathybiaster loripes** | **Camille Moreau** |  | **2006** | **-62.53** | **-61.82** | **Sshet** | **190** |
| **Psi189** | **Psi189** | **JR144** | **Psilaster charcoti** | **Camille Moreau** |  | **2006** | **-62.53** | **-61.82** | **Sshet** | **190** |
| **Psi190** | **Psi190** | **JR144** | **Psilaster charcoti** | **Camille Moreau** |  | **2006** | **-62.53** | **-61.82** | **Sshet** | **190** |
| **Psi191** | **Psi191** | **JR144** | **Psilaster charcoti** | **Camille Moreau** |  | **2006** | **-62.53** | **-61.82** | **Sshet** | **190** |
| **Psi192** | **Psi192** | **JR144** | **Psilaster charcoti** | **Camille Moreau** |  | **2006** | **-62.53** | **-61.82** | **Sshet** | **190** |
| **Bat219** | **Bat219** | **LASSO_ANTXXIX/3** | **Bathybiaster loripes** | **Christopher L. Mah** |  | **2013** | **-62.29** | **-61.20** | **Sshet** | **251,0** |
| **Bat220** | **Bat220** | **LASSO_ANTXXIX/3** | **Bathybiaster loripes** | **Christopher L. Mah** |  | **2013** | **-62.29** | **-61.20** | **Sshet** | **251,0** |
| **Bat221** | **Bat221** | **LASSO_ANTXXIX/3** | **Bathybiaster loripes** | **Christopher L. Mah** |  | **2013** | **-62.29** | **-61.20** | **Sshet** | **251,0** |
| **Dip221** | **Dip221** | **LASSO_ANTXXIX/3** | **Diplasterias brucei** | **Christopher L. Mah** |  | **2013** | **-62.29** | **-61.20** | **Sshet** | **251,0** |
| **Dip222** | **Dip222** | **LASSO_ANTXXIX/3** | **Diplasterias brucei** | **Christopher L. Mah** |  | **2013** | **-62.29** | **-61.20** | **Sshet** | **251,0** |
| **Dip223** | **Dip223** | **LASSO_ANTXXIX/3** | **Diplasterias brucei** | **Christopher L. Mah** |  | **2013** | **-62.29** | **-61.20** | **Sshet** | **251,0** |
| **Lys060** | **Lys060** | **LASSO_ANTXXIX/3** | **Lysasterias sp** | **Christopher L. Mah** |  | **2013** | **-62.29** | **-61.20** | **Sshet** | **251,0** |
| **Lys061** | **Lys061** | **LASSO_ANTXXIX/3** | **Lysasterias sp** | **Christopher L. Mah** |  | **2013** | **-62.29** | **-61.20** | **Sshet** | **251,0** |
| **Lys064** | **Lys064** | **LASSO_ANTXXIX/3** | **Lysasterias sp** | **Christopher L. Mah** |  | **2013** | **-62.29** | **-61.20** | **Sshet** | **251,0** |
| **Lys065** | **Lys065** | **LASSO_ANTXXIX/3** | **Lysasterias sp** | **Christopher L. Mah** |  | **2013** | **-62.29** | **-61.20** | **Sshet** | **251,0** |
| **Lys066** | **Lys066** | **LASSO_ANTXXIX/3** | **Lysasterias sp** | **Christopher L. Mah** |  | **2013** | **-62.29** | **-61.20** | **Sshet** | **251,0** |
| **Lys068** | **Lys068** | **LASSO_ANTXXIX/3** | **Lysasterias sp** | **Christopher L. Mah** |  | **2013** | **-62.29** | **-61.20** | **Sshet** | **251,0** |
| **Lys069** | **Lys069** | **LASSO_ANTXXIX/3** | **Lysasterias sp** | **Christopher L. Mah** |  | **2013** | **-62.29** | **-61.20** | **Sshet** | **251,0** |
| **Lys071** | **Lys071** | **LASSO_ANTXXIX/3** | **Lysasterias sp** | **Christopher L. Mah** |  | **2013** | **-62.29** | **-61.20** | **Sshet** | **251,0** |
| **Lys073** | **Lys073** | **LASSO_ANTXXIX/3** | **Lysasterias sp** | **Christopher L. Mah** |  | **2013** | **-62.29** | **-61.20** | **Sshet** | **251,0** |
| **Lys074** | **Lys074** | **LASSO_ANTXXIX/3** | **Lysasterias sp** | **Christopher L. Mah** |  | **2013** | **-62.29** | **-61.20** | **Sshet** | **251,0** |
| **Lys198** | **Lys198** | **LASSO_ANTXXIX/3** | **Lysasterias sp** | **Christopher L. Mah** |  | **2013** | **-62.29** | **-61.20** | **Sshet** | **251,0** |
| **Lys199** | **Lys199** | **LASSO_ANTXXIX/3** | **Lysasterias sp** | **Christopher L. Mah** |  | **2013** | **-62.29** | **-61.20** | **Sshet** | **251,0** |
| **Lys200** | **Lys200** | **LASSO_ANTXXIX/3** | **Lysasterias sp** | **Christopher L. Mah** |  | **2013** | **-62.29** | **-61.20** | **Sshet** | **251,0** |
| **Lys201** | **Lys201** | **LASSO_ANTXXIX/3** | **Lysasterias sp** | **Christopher L. Mah** |  | **2013** | **-62.29** | **-61.20** | **Sshet** | **251,0** |
| **Lys202** | **Lys202** | **LASSO_ANTXXIX/3** | **Lysasterias sp** | **Christopher L. Mah** |  | **2013** | **-62.29** | **-61.20** | **Sshet** | **251,0** |
| **Lys203** | **Lys203** | **LASSO_ANTXXIX/3** | **Lysasterias sp** | **Christopher L. Mah** |  | **2013** | **-62.29** | **-61.20** | **Sshet** | **251,0** |
| **Not197** | **Not197** | **LASSO_ANTXXIX/3** | **Notasterias bongraini** | **Christopher L. Mah** |  | **2013** | **-62.29** | **-61.20** | **Sshet** | **251,0** |
| **Not198** | **Not198** | **LASSO_ANTXXIX/3** | **Notasterias bongraini** | **Christopher L. Mah** |  | **2013** | **-62.29** | **-61.20** | **Sshet** | **251,0** |
| **Not199** | **Not199** | **LASSO_ANTXXIX/3** | **Notasterias bongraini** | **Christopher L. Mah** |  | **2013** | **-62.29** | **-61.20** | **Sshet** | **251,0** |
| **Psi057** | **Psi057** | **LASSO_ANTXXIX/3** | **Psilaster charcoti** | **Christopher L. Mah** |  | **2013** | **-62.29** | **-61.20** | **Sshet** | **251,0** |
| **Psi058** | **Psi058** | **LASSO_ANTXXIX/3** | **Psilaster charcoti** | **Christopher L. Mah** |  | **2013** | **-62.29** | **-61.20** | **Sshet** | **251,0** |
| **Psi059** | **Psi059** | **LASSO_ANTXXIX/3** | **Psilaster charcoti** | **Christopher L. Mah** |  | **2013** | **-62.29** | **-61.20** | **Sshet** | **251,0** |
| **Psi060** | **Psi060** | **LASSO_ANTXXIX/3** | **Psilaster charcoti** | **Christopher L. Mah** |  | **2013** | **-62.29** | **-61.20** | **Sshet** | **251,0** |
| **Psi061** | **Psi061** | **LASSO_ANTXXIX/3** | **Psilaster charcoti** | **Christopher L. Mah** |  | **2013** | **-62.29** | **-61.20** | **Sshet** | **251,0** |
| **Lys127** | **Lys127** | **LASSO_ANTXXIX/3** | **Lysasterias sp** | **Christopher L. Mah** |  | **2013** | **-62.27** | **-61.18** | **Sshet** | **392,0** |
| **Bat265** | **Bat265** | **JR144** | **Bathybiaster loripes** | **Camille Moreau** |  | **2006** | **-62.92** | **-61.00** | **Sshet** | **199** |
| **Dip044** | **Dip044** | **JR144** | **Undet (cf diplasterias)** | **Camille Moreau** |  | **2006** | **-62.92** | **-61.00** | **Sshet** | **199** |
| **Dip046** | **Dip046** | **JR144** | **Undet (cf diplasterias)** | **Camille Moreau** |  | **2006** | **-62.92** | **-61.00** | **Sshet** | **199** |
| **Dip049** | **Dip049** | **JR144** | **Undet (cf diplasterias)** | **Camille Moreau** |  | **2006** | **-62.92** | **-61.00** | **Sshet** | **199** |
| **Dip050** | **Dip050** | **JR144** | **Undet (cf diplasterias)** | **Camille Moreau** |  | **2006** | **-62.92** | **-61.00** | **Sshet** | **199** |
| **Dip054** | **Dip054** | **JR144** | **Undet (cf diplasterias)** | **Camille Moreau** |  | **2006** | **-62.92** | **-61.00** | **Sshet** | **199** |
| **Dip056** | **Dip056** | **JR144** | **Undet (cf diplasterias)** | **Camille Moreau** |  | **2006** | **-62.92** | **-61.00** | **Sshet** | **199** |
| **Dip057** | **Dip057** | **JR144** | **Undet (cf diplasterias)** | **Camille Moreau** |  | **2006** | **-62.92** | **-61.00** | **Sshet** | **199** |
| **Dip058** | **Dip058** | **JR144** | **Undet (cf diplasterias)** | **Camille Moreau** |  | **2006** | **-62.92** | **-61.00** | **Sshet** | **199** |
| **Dip059** | **Dip059** | **JR144** | **Undet (cf diplasterias)** | **Camille Moreau** |  | **2006** | **-62.92** | **-61.00** | **Sshet** | **199** |
| **Dip060** | **Dip060** | **JR144** | **Undet (cf diplasterias)** | **Camille Moreau** |  | **2006** | **-62.92** | **-61.00** | **Sshet** | **199** |
| **Dip061** | **Dip061** | **JR144** | **Undet (cf diplasterias)** | **Camille Moreau** |  | **2006** | **-62.92** | **-61.00** | **Sshet** | **199** |
| **Dip062** | **Dip062** | **JR144** | **Undet (cf diplasterias)** | **Camille Moreau** |  | **2006** | **-62.92** | **-61.00** | **Sshet** | **199** |
| **Dip063** | **Dip063** | **JR144** | **Undet (cf diplasterias)** | **Camille Moreau** |  | **2006** | **-62.92** | **-61.00** | **Sshet** | **199** |
| **Dip064** | **Dip064** | **JR144** | **Undet (cf diplasterias)** | **Camille Moreau** |  | **2006** | **-62.92** | **-61.00** | **Sshet** | **199** |
| **Dip065** | **Dip065** | **JR144** | **Undet (cf diplasterias)** | **Camille Moreau** |  | **2006** | **-62.92** | **-61.00** | **Sshet** | **199** |
| **Dip066** | **Dip066** | **JR144** | **Undet (cf diplasterias)** | **Camille Moreau** |  | **2006** | **-62.92** | **-61.00** | **Sshet** | **199** |
| **Dip067** | **Dip067** | **JR144** | **Undet (cf diplasterias)** | **Camille Moreau** |  | **2006** | **-62.92** | **-61.00** | **Sshet** | **199** |
| **Dip068** | **Dip068** | **JR144** | **Undet (cf diplasterias)** | **Camille Moreau** |  | **2006** | **-62.92** | **-61.00** | **Sshet** | **199** |
| **Dip069** | **Dip069** | **JR144** | **Undet (cf diplasterias)** | **Camille Moreau** |  | **2006** | **-62.92** | **-61.00** | **Sshet** | **199** |
| **Dip070** | **Dip070** | **JR144** | **Undet (cf diplasterias)** | **Camille Moreau** |  | **2006** | **-62.92** | **-61.00** | **Sshet** | **199** |
| **Dip071** | **Dip071** | **JR144** | **Undet (cf diplasterias)** | **Camille Moreau** |  | **2006** | **-62.92** | **-61.00** | **Sshet** | **199** |
| **Dip072** | **Dip072** | **JR144** | **Undet (cf diplasterias)** | **Camille Moreau** |  | **2006** | **-62.92** | **-61.00** | **Sshet** | **199** |
| **Dip073** | **Dip073** | **JR144** | **Undet (cf diplasterias)** | **Camille Moreau** |  | **2006** | **-62.92** | **-61.00** | **Sshet** | **199** |
| **Dip074** | **Dip074** | **JR144** | **Undet (cf diplasterias)** | **Camille Moreau** |  | **2006** | **-62.92** | **-61.00** | **Sshet** | **199** |
| **Dip075** | **Dip075** | **JR144** | **Undet (cf diplasterias)** | **Camille Moreau** |  | **2006** | **-62.92** | **-61.00** | **Sshet** | **199** |
| **Dip076** | **Dip076** | **JR144** | **Undet (cf diplasterias)** | **Camille Moreau** |  | **2006** | **-62.92** | **-61.00** | **Sshet** | **199** |
| **Dip077** | **Dip077** | **JR144** | **Undet (cf diplasterias)** | **Camille Moreau** |  | **2006** | **-62.92** | **-61.00** | **Sshet** | **199** |
| **Bat031** | **Bat031** | **LASSO_ANTXXIX/3** | **Bathybiaster loripes** | **Christopher L. Mah** |  | **2013** | **-62.13** | **-60.65** | **Sshet** | **464,0** |
| **Bat032** | **Bat032** | **LASSO_ANTXXIX/3** | **Bathybiaster loripes** | **Christopher L. Mah** |  | **2013** | **-62.13** | **-60.65** | **Sshet** | **464,0** |
| **Lys123** | **Lys123** | **LASSO_ANTXXIX/3** | **Lysasterias sp** | **Christopher L. Mah** |  | **2013** | **-62.13** | **-60.65** | **Sshet** | **464,0** |
| **Lys101** | **Lys101** | **LASSO_ANTXXIX/3** | **Lysasterias sp** | **Christopher L. Mah** |  | **2013** | **-62.12** | **-60.56** | **Sshet** | **277,0** |
| **Lys102** | **Lys102** | **LASSO_ANTXXIX/3** | **Lysasterias sp** | **Christopher L. Mah** |  | **2013** | **-62.12** | **-60.56** | **Sshet** | **277,0** |
| **Lys103** | **Lys103** | **LASSO_ANTXXIX/3** | **Lysasterias sp** | **Christopher L. Mah** |  | **2013** | **-62.12** | **-60.56** | **Sshet** | **277,0** |
| **Psi135** | **Psi135** | **LASSO_ANTXXIX/3** | **Psilaster charcoti** | **Christopher L. Mah** |  | **2013** | **-62.12** | **-60.56** | **Sshet** | **277,0** |
| **Psi136** | **Psi136** | **LASSO_ANTXXIX/3** | **Psilaster charcoti** | **Christopher L. Mah** |  | **2013** | **-62.12** | **-60.56** | **Sshet** | **277,0** |
| **Lys158** | **Lys158** | **LASSO_ANTXXIX/3** | **Lysasterias sp** | **Christopher L. Mah** |  | **2013** | **-61.93** | **-60.09** | **Sshet** | **423,0** |
| **Lys159** | **Lys159** | **LASSO_ANTXXIX/3** | **Lysasterias sp** | **Christopher L. Mah** |  | **2013** | **-61.93** | **-60.09** | **Sshet** | **423,0** |
| **Lys160** | **Lys160** | **LASSO_ANTXXIX/3** | **Lysasterias sp** | **Christopher L. Mah** |  | **2013** | **-61.93** | **-60.09** | **Sshet** | **423,0** |
| **Lys161** | **Lys161** | **LASSO_ANTXXIX/3** | **Lysasterias sp** | **Christopher L. Mah** |  | **2013** | **-61.93** | **-60.09** | **Sshet** | **423,0** |
| **Lys164** | **Lys164** | **LASSO_ANTXXIX/3** | **Lysasterias sp** | **Christopher L. Mah** |  | **2013** | **-61.93** | **-60.09** | **Sshet** | **423,0** |
| **Lys166** | **Lys166** | **LASSO_ANTXXIX/3** | **Lysasterias sp** | **Christopher L. Mah** |  | **2013** | **-61.93** | **-60.09** | **Sshet** | **423,0** |
| **Psi124** | **Psi124** | **LASSO_ANTXXIX/3** | **Bathybiaster loripes** | **Christopher L. Mah** |  | **2013** | **-61.93** | **-60.09** | **Sshet** | **423,0** |
| **Psi128** | **Psi128** | **LASSO_ANTXXIX/3** | **Psilaster charcoti** | **Christopher L. Mah** |  | **2013** | **-61.93** | **-60.09** | **Sshet** | **423,0** |
| **Bat076** | **Bat076** | **LASSO_ANTXXIX/3** | **Bathybiaster loripes** | **Christopher L. Mah** |  | **2013** | **-62.00** | **-60.06** | **Sshet** | **270,0** |
| **Bat077** | **Bat077** | **LASSO_ANTXXIX/3** | **Bathybiaster loripes** | **Christopher L. Mah** |  | **2013** | **-62.00** | **-60.06** | **Sshet** | **270,0** |
| **Bat106** | **Bat106** | **LASSO_ANTXXIX/3** | **Bathybiaster loripes** | **Christopher L. Mah** |  | **2013** | **-62.00** | **-60.06** | **Sshet** | **270,0** |
| **Lys212** | **Lys212** | **LASSO_ANTXXIX/3** | **Lysasterias sp** | **Christopher L. Mah** |  | **2013** | **-62.00** | **-60.06** | **Sshet** | **270,0** |
| **Not201** | **Not201** | **LASSO_ANTXXIX/3** | **Notasterias bongraini** | **Christopher L. Mah** |  | **2013** | **-62.00** | **-60.06** | **Sshet** | **270,0** |
| **Not202** | **Not202** | **LASSO_ANTXXIX/3** | **Notasterias bongraini** | **Christopher L. Mah** |  | **2013** | **-62.00** | **-60.06** | **Sshet** | **270,0** |
| **Not203** | **Not203** | **LASSO_ANTXXIX/3** | **Notasterias bongraini** | **Christopher L. Mah** |  | **2013** | **-62.00** | **-60.06** | **Sshet** | **270,0** |
| **Not204** | **Not204** | **LASSO_ANTXXIX/3** | **Notasterias bongraini** | **Christopher L. Mah** |  | **2013** | **-62.00** | **-60.06** | **Sshet** | **270,0** |
| **Not205** | **Not205** | **LASSO_ANTXXIX/3** | **Notasterias bongraini** | **Christopher L. Mah** |  | **2013** | **-62.00** | **-60.06** | **Sshet** | **270,0** |
| **Not206** | **Not206** | **LASSO_ANTXXIX/3** | **Notasterias bongraini** | **Christopher L. Mah** |  | **2013** | **-62.00** | **-60.06** | **Sshet** | **270,0** |
| **Not207** | **Not207** | **LASSO_ANTXXIX/3** | **Notasterias bongraini** | **Christopher L. Mah** |  | **2013** | **-62.00** | **-60.06** | **Sshet** | **270,0** |
| **Not208** | **Not208** | **LASSO_ANTXXIX/3** | **Notasterias bongraini** | **Christopher L. Mah** |  | **2013** | **-62.00** | **-60.06** | **Sshet** | **270,0** |
| **Not209** | **Not209** | **LASSO_ANTXXIX/3** | **Notasterias bongraini** | **Christopher L. Mah** |  | **2013** | **-62.00** | **-60.06** | **Sshet** | **270,0** |
| **Not210** | **Not210** | **LASSO_ANTXXIX/3** | **Notasterias bongraini** | **Christopher L. Mah** |  | **2013** | **-62.00** | **-60.06** | **Sshet** | **270,0** |
| **Not211** | **Not211** | **LASSO_ANTXXIX/3** | **Notasterias bongraini** | **Christopher L. Mah** |  | **2013** | **-62.00** | **-60.06** | **Sshet** | **270,0** |
| **Not212** | **Not212** | **LASSO_ANTXXIX/3** | **Notasterias bongraini** | **Christopher L. Mah** |  | **2013** | **-62.00** | **-60.06** | **Sshet** | **270,0** |
| **Not213** | **Not213** | **LASSO_ANTXXIX/3** | **Notasterias bongraini** | **Christopher L. Mah** |  | **2013** | **-62.00** | **-60.06** | **Sshet** | **270,0** |
| **Not214** | **Not214** | **LASSO_ANTXXIX/3** | **Notasterias bongraini** | **Christopher L. Mah** |  | **2013** | **-62.00** | **-60.06** | **Sshet** | **270,0** |
| **Not215** | **Not215** | **LASSO_ANTXXIX/3** | **Notasterias bongraini** | **Christopher L. Mah** |  | **2013** | **-62.00** | **-60.06** | **Sshet** | **270,0** |
| **Not216** | **Not216** | **LASSO_ANTXXIX/3** | **Notasterias bongraini** | **Christopher L. Mah** |  | **2013** | **-62.00** | **-60.06** | **Sshet** | **270,0** |
| **Not217** | **Not217** | **LASSO_ANTXXIX/3** | **Notasterias bongraini** | **Christopher L. Mah** |  | **2013** | **-62.00** | **-60.06** | **Sshet** | **270,0** |
| **Not219** | **Not219** | **LASSO_ANTXXIX/3** | **Notasterias bongraini** | **Christopher L. Mah** |  | **2013** | **-62.00** | **-60.06** | **Sshet** | **270,0** |
| **Not220** | **Not220** | **LASSO_ANTXXIX/3** | **Notasterias bongraini** | **Christopher L. Mah** |  | **2013** | **-62.00** | **-60.06** | **Sshet** | **270,0** |
| **Not221** | **Not221** | **LASSO_ANTXXIX/3** | **Notasterias bongraini** | **Christopher L. Mah** |  | **2013** | **-62.00** | **-60.06** | **Sshet** | **270,0** |
| **Not222** | **Not222** | **LASSO_ANTXXIX/3** | **Notasterias bongraini** | **Christopher L. Mah** |  | **2013** | **-62.00** | **-60.06** | **Sshet** | **270,0** |
| **Not223** | **Not223** | **LASSO_ANTXXIX/3** | **Notasterias bongraini** | **Christopher L. Mah** |  | **2013** | **-62.00** | **-60.06** | **Sshet** | **270,0** |
| **Not250** | **Not250** | **LASSO_ANTXXIX/3** | **Notasterias bongraini** | **Christopher L. Mah** |  | **2013** | **-62.00** | **-60.06** | **Sshet** | **270,0** |
| **Not251** | **Not251** | **LASSO_ANTXXIX/3** | **Notasterias bongraini** | **Christopher L. Mah** |  | **2013** | **-62.00** | **-60.06** | **Sshet** | **270,0** |
| **Not252** | **Not252** | **LASSO_ANTXXIX/3** | **Notasterias bongraini** | **Christopher L. Mah** |  | **2013** | **-62.00** | **-60.06** | **Sshet** | **270,0** |
| **Not253** | **Not253** | **LASSO_ANTXXIX/3** | **Notasterias bongraini** | **Christopher L. Mah** |  | **2013** | **-62.00** | **-60.06** | **Sshet** | **270,0** |
| **Psi129** | **Psi129** | **LASSO_ANTXXIX/3** | **Bathybiaster loripes** | **Christopher L. Mah** |  | **2013** | **-62.00** | **-60.06** | **Sshet** | **270,0** |
| **Psi131** | **Psi131** | **LASSO_ANTXXIX/3** | **Psilaster charcoti** | **Christopher L. Mah** |  | **2013** | **-62.00** | **-60.06** | **Sshet** | **270,0** |
| **Psi132** | **Psi132** | **LASSO_ANTXXIX/3** | **Psilaster charcoti** | **Christopher L. Mah** |  | **2013** | **-62.00** | **-60.06** | **Sshet** | **270,0** |
| **Psi133** | **Psi133** | **LASSO_ANTXXIX/3** | **Psilaster charcoti** | **Christopher L. Mah** |  | **2013** | **-62.00** | **-60.06** | **Sshet** | **270,0** |
| **Psi134** | **Psi134** | **LASSO_ANTXXIX/3** | **Psilaster charcoti** | **Christopher L. Mah** |  | **2013** | **-62.00** | **-60.06** | **Sshet** | **270,0** |
| **Psi137** | **Psi137** | **LASSO_ANTXXIX/3** | **Psilaster charcoti** | **Christopher L. Mah** |  | **2013** | **-62.00** | **-60.06** | **Sshet** | **270,0** |
| **Psi138** | **Psi138** | **LASSO_ANTXXIX/3** | **Psilaster charcoti** | **Christopher L. Mah** |  | **2013** | **-62.00** | **-60.06** | **Sshet** | **270,0** |
| **Bat074** | **Bat074** | **LASSO_ANTXXIX/3** | **Bathybiaster loripes** | **Christopher L. Mah** |  | **2013** | **-62.93** | **-58.68** | **Bransfield** | **564** |
| **Bat075** | **Bat075** | **LASSO_ANTXXIX/3** | **Bathybiaster loripes** | **Christopher L. Mah** |  | **2013** | **-62.93** | **-58.68** | **Bransfield** | **564** |
| **Dip214** | **Dip214** | **LASSO_ANTXXIX/3** | **Lysasterias sp** | **Christopher L. Mah** |  | **2013** | **-62.93** | **-58.68** | **Bransfield** | **564** |
| **Dip215** | **Dip215** | **LASSO_ANTXXIX/3** | **Lysasterias sp** | **Christopher L. Mah** |  | **2013** | **-62.93** | **-58.68** | **Bransfield** | **564** |
| **Not246** | **Not246** | **LASSO_ANTXXIX/3** | **Notasterias bongraini** | **Christopher L. Mah** |  | **2013** | **-62.93** | **-58.68** | **Bransfield** | **564** |
| **Not247** | **Not247** | **LASSO_ANTXXIX/3** | **Notasterias bongraini** | **Christopher L. Mah** |  | **2013** | **-62.93** | **-58.68** | **Bransfield** | **564** |
| **Not257** | **Not257** | **LASSO_ANTXXIX/3** | **Notasterias bongraini** | **Christopher L. Mah** |  | **2013** | **-62.93** | **-58.68** | **Bransfield** | **564** |
| **Not258** | **Not258** | **LASSO_ANTXXIX/3** | **Notasterias bongraini** | **Christopher L. Mah** |  | **2013** | **-62.93** | **-58.68** | **Bransfield** | **564** |
| **Psi127** | **Psi127** | **LASSO_ANTXXIX/3** | **Psilaster charcoti** | **Christopher L. Mah** |  | **2013** | **-62.93** | **-58.68** | **Bransfield** | **564** |
| **Bat079** | **Bat079** | **LASSO_ANTXXIX/3** | **Bathybiaster loripes** | **Christopher L. Mah** |  | **2013** | **-63.01** | **-58.59** | **Bransfield** | **261** |
| **Bat080** | **Bat080** | **LASSO_ANTXXIX/3** | **Bathybiaster loripes** | **Christopher L. Mah** |  | **2013** | **-63.01** | **-58.59** | **Bransfield** | **261** |
| **Dip177** | **Dip177** | **LASSO_ANTXXIX/3** | **Diplasterias brucei** | **Christopher L. Mah** |  | **2013** | **-63.01** | **-58.59** | **Bransfield** | **261** |
| **Dip178** | **Dip178** | **LASSO_ANTXXIX/3** | **Diplasterias brucei** | **Christopher L. Mah** |  | **2013** | **-63.01** | **-58.59** | **Bransfield** | **261** |
| **Lys168** | **Lys168** | **LASSO_ANTXXIX/3** | **Lysasterias heteractis** | **Christopher L. Mah** |  | **2013** | **-63.01** | **-58.59** | **Bransfield** | **261** |
| **Not173** | **Not173** | **LASSO_ANTXXIX/3** | **Notasterias armata** | **Christopher L. Mah** |  | **2013** | **-63.01** | **-58.59** | **Bransfield** | **261** |
| **Not174** | **Not174** | **LASSO_ANTXXIX/3** | **Notasterias armata** | **Christopher L. Mah** |  | **2013** | **-63.01** | **-58.59** | **Bransfield** | **261** |
| **Not175** | **Not175** | **LASSO_ANTXXIX/3** | **Notasterias armata** | **Christopher L. Mah** |  | **2013** | **-63.01** | **-58.59** | **Bransfield** | **261** |
| **Not176** | **Not176** | **LASSO_ANTXXIX/3** | **Notasterias armata** | **Christopher L. Mah** |  | **2013** | **-63.01** | **-58.59** | **Bransfield** | **261** |
| **Bat029** | **Bat029** | **LASSO_ANTXXIX/3** | **Bathybiaster loripes** | **Christopher L. Mah** |  | **2013** | **-62.95** | **-58.39** | **Bransfield** | **782** |
| **Not191** | **Not191** | **LASSO_ANTXXIX/3** | **Notasterias bongraini** | **Christopher L. Mah** |  | **2013** | **-62.95** | **-58.39** | **Bransfield** | **782** |
| **Not192** | **Not192** | **LASSO_ANTXXIX/3** | **Notasterias bongraini** | **Christopher L. Mah** |  | **2013** | **-62.95** | **-58.39** | **Bransfield** | **782** |
| **Not193** | **Not193** | **LASSO_ANTXXIX/3** | **Notasterias bongraini** | **Christopher L. Mah** |  | **2013** | **-62.95** | **-58.39** | **Bransfield** | **782** |
| **Not194** | **Not194** | **LASSO_ANTXXIX/3** | **Notasterias bongraini** | **Christopher L. Mah** |  | **2013** | **-62.95** | **-58.39** | **Bransfield** | **782** |
| **Not195** | **Not195** | **LASSO_ANTXXIX/3** | **Notasterias bongraini** | **Christopher L. Mah** |  | **2013** | **-62.95** | **-58.39** | **Bransfield** | **782** |
| **Lys029** | **Lys029** | **Carlini2016** | **Lysasterias sp** | **Camille Moreau** |  | **2016** | **-62.10** | **-58.34** | **Sshet** | **10** |
| **Lys031** | **Lys031** | **Carlini2016** | **Lysasterias sp** | **Camille Moreau** |  | **2016** | **-62.10** | **-58.34** | **Sshet** | **10** |
| **Bat083** | **Bat083** | **LASSO_ANTXXIX/3** | **Bathybiaster loripes** | **Christopher L. Mah** |  | **2013** | **-62.95** | **-58.24** | **Bransfield** | **325** |
| **Dip182** | **Dip182** | **LASSO_ANTXXIX/3** | **Diplasterias brucei** | **Christopher L. Mah** |  | **2013** | **-62.95** | **-58.24** | **Bransfield** | **325** |
| **Dip183** | **Dip183** | **LASSO_ANTXXIX/3** | **Diplasterias brucei** | **Christopher L. Mah** |  | **2013** | **-62.95** | **-58.24** | **Bransfield** | **325** |
| **Lys098** | **Lys098** | **LASSO_ANTXXIX/3** | **Lysasterias sp** | **Christopher L. Mah** |  | **2013** | **-62.95** | **-58.24** | **Bransfield** | **325** |
| **Not255** | **Not255** | **LASSO_ANTXXIX/3** | **Notasterias bongraini** | **Christopher L. Mah** |  | **2013** | **-62.95** | **-58.24** | **Bransfield** | **325** |
| **Dip196** | **Dip196** | **LASSO_ANTXXIX/3** | **Diplasterias brucei** | **Christopher L. Mah** |  | **2013** | **-62.89** | **-58.22** | **Bransfield** | **461** |
| **Dip198** | **Dip198** | **LASSO_ANTXXIX/3** | **Diplasterias brucei** | **Christopher L. Mah** |  | **2013** | **-62.89** | **-58.22** | **Bransfield** | **461** |
| **Lys120** | **Lys120** | **LASSO_ANTXXIX/3** | **Lysasterias sp** | **Christopher L. Mah** |  | **2013** | **-62.89** | **-58.22** | **Bransfield** | **461** |
| **Bat082** | **Bat082** | **LASSO_ANTXXIX/3** | **Bathybiaster loripes** | **Christopher L. Mah** |  | **2013** | **-63.03** | **-58.05** | **Bransfield** | **176** |
| **Dip181** | **Dip181** | **LASSO_ANTXXIX/3** | **Diplasterias brucei** | **Christopher L. Mah** |  | **2013** | **-63.03** | **-58.05** | **Bransfield** | **176** |
| **Dip212** | **Dip212** | **LASSO_ANTXXIX/3** | **Diplasterias brucei** | **Christopher L. Mah** |  | **2013** | **-62.93** | **-57.97** | **Bransfield** | **781** |
| **Dip213** | **Dip213** | **LASSO_ANTXXIX/3** | **Diplasterias brucei** | **Christopher L. Mah** |  | **2013** | **-62.93** | **-57.97** | **Bransfield** | **781** |
| **Not238** | **Not238** | **LASSO_ANTXXIX/3** | **Notasterias bongraini** | **Christopher L. Mah** |  | **2013** | **-62.93** | **-57.97** | **Bransfield** | **781** |
| **Not239** | **Not239** | **LASSO_ANTXXIX/3** | **Notasterias bongraini** | **Christopher L. Mah** |  | **2013** | **-62.93** | **-57.97** | **Bransfield** | **781** |
| **Not240** | **Not240** | **LASSO_ANTXXIX/3** | **Notasterias bongraini** | **Christopher L. Mah** |  | **2013** | **-62.93** | **-57.97** | **Bransfield** | **781** |
| **Dip220** | **Dip220** | **LASSO_ANTXXIX/3** | **Diplasterias brucei** | **Christopher L. Mah** |  | **2013** | **-62.73** | **-57.47** | **Bransfield** | **423** |
| **Dip200** | **Dip200** | **LASSO_ANTXXIX/3** | **Diplasterias brucei** | **Christopher L. Mah** |  | **2013** | **-62.72** | **-57.45** | **Bransfield** | **412** |
| **Dip201** | **Dip201** | **LASSO_ANTXXIX/3** | **Diplasterias brucei** | **Christopher L. Mah** |  | **2013** | **-62.72** | **-57.45** | **Bransfield** | **412** |
| **Bat108** | **Bat108** | **LASSO_ANTXXIX/3** | **Bathybiaster loripes** | **Christopher L. Mah** |  | **2013** | **-62.74** | **-57.44** | **Bransfield** | **286** |
| **Dip179** | **Dip179** | **LASSO_ANTXXIX/3** | **Diplasterias brucei** | **Christopher L. Mah** |  | **2013** | **-62.74** | **-57.44** | **Bransfield** | **286** |
| **Dip180** | **Dip180** | **LASSO_ANTXXIX/3** | **Diplasterias brucei** | **Christopher L. Mah** |  | **2013** | **-62.74** | **-57.44** | **Bransfield** | **286** |
| **Dip218** | **Dip218** | **LASSO_ANTXXIX/3** | **Diplasterias brucei** | **Christopher L. Mah** |  | **2013** | **-62.74** | **-57.44** | **Bransfield** | **286** |
| **Lys076** | **Lys076** | **LASSO_ANTXXIX/3** | **Lysasterias sp** | **Christopher L. Mah** |  | **2013** | **-62.74** | **-57.44** | **Bransfield** | **286** |
| **Lys078** | **Lys078** | **LASSO_ANTXXIX/3** | **Lysasterias sp** | **Christopher L. Mah** |  | **2013** | **-62.74** | **-57.44** | **Bransfield** | **286** |
| **Lys080** | **Lys080** | **LASSO_ANTXXIX/3** | **Lysasterias sp** | **Christopher L. Mah** |  | **2013** | **-62.74** | **-57.44** | **Bransfield** | **286** |
| **Not243** | **Not243** | **LASSO_ANTXXIX/3** | **Notasterias bongraini** | **Christopher L. Mah** |  | **2013** | **-62.74** | **-57.44** | **Bransfield** | **286** |
| **Not190** | **Not190** | **LASSO_ANTXXIX/3** | **Notasterias bongraini** | **Christopher L. Mah** |  | **2013** | **-62.74** | **-57.43** | **Bransfield** | **277** |
| **Dip042** | **Dip042** | **JR144** | **Undet (cf diplasterias)** | **Camille Moreau** |  | **2006** | **-61.97** | **-57.24** | **Sshet** | **130** |
| **Lys028** | **Lys028** | **JR144** | **Undet** | **Camille Moreau** |  | **2006** | **-61.97** | **-57.24** | **Sshet** | **130** |
| **Lys167** | **Lys167** | **LASSO_ANTXXIX/3** | **Lysasterias sp** | **Christopher L. Mah** |  | **2013** | **-62.80** | **-57.09** | **Bransfield** | **580** |
| **Psi126** | **Psi126** | **LASSO_ANTXXIX/3** | **Psilaster charcoti** | **Christopher L. Mah** |  | **2013** | **-62.80** | **-57.09** | **Bransfield** | **580** |
| **Bat218** | **Bat218** | **LASSO_ANTXXIX/3** | **Bathybiaster loripes** | **Christopher L. Mah** |  | **2013** | **-63.98** | **-56.77** | **TipPen** | **217** |
| **Dip224** | **Dip224** | **LASSO_ANTXXIX/3** | **Diplasterias brucei** | **Christopher L. Mah** |  | **2013** | **-63.98** | **-56.77** | **TipPen** | **217** |
| **Dip225** | **Dip225** | **LASSO_ANTXXIX/3** | **Diplasterias brucei** | **Christopher L. Mah** |  | **2013** | **-63.98** | **-56.77** | **TipPen** | **217** |
| **Psi176** | **Psi176** | **LASSO_ANTXXIX/3** | **Psilaster charcoti** | **Christopher L. Mah** |  | **2013** | **-63.98** | **-56.77** | **TipPen** | **217** |
| **Psi177** | **Psi177** | **LASSO_ANTXXIX/3** | **Psilaster charcoti** | **Christopher L. Mah** |  | **2013** | **-63.98** | **-56.77** | **TipPen** | **217** |
| **Psi178** | **Psi178** | **LASSO_ANTXXIX/3** | **Psilaster charcoti** | **Christopher L. Mah** |  | **2013** | **-63.98** | **-56.77** | **TipPen** | **217** |
| **Psi179** | **Psi179** | **LASSO_ANTXXIX/3** | **Psilaster charcoti** | **Christopher L. Mah** |  | **2013** | **-63.98** | **-56.77** | **TipPen** | **217** |
| **Psi180** | **Psi180** | **LASSO_ANTXXIX/3** | **Psilaster charcoti** | **Christopher L. Mah** |  | **2013** | **-63.98** | **-56.77** | **TipPen** | **217** |
| **Psi181** | **Psi181** | **LASSO_ANTXXIX/3** | **Psilaster charcoti** | **Christopher L. Mah** |  | **2013** | **-63.98** | **-56.77** | **TipPen** | **217** |
| **Psi182** | **Psi182** | **LASSO_ANTXXIX/3** | **Psilaster charcoti** | **Christopher L. Mah** |  | **2013** | **-63.98** | **-56.77** | **TipPen** | **217** |
| **Psi183** | **Psi183** | **LASSO_ANTXXIX/3** | **Psilaster charcoti** | **Christopher L. Mah** |  | **2013** | **-63.98** | **-56.77** | **TipPen** | **217** |
| **Dip051** | **Dip051** | **JR144** | **Undet (cf diplasterias)** | **Camille Moreau** |  | **2006** | **-54.31** | **-56.68** | **Mag** | **201** |
| **Dip052** | **Dip052** | **JR144** | **Undet (cf diplasterias)** | **Camille Moreau** |  | **2006** | **-54.31** | **-56.68** | **Mag** | **201** |
| **Dip053** | **Dip053** | **JR144** | **Undet (cf diplasterias)** | **Camille Moreau** |  | **2006** | **-54.31** | **-56.68** | **Mag** | **201** |
| **Psi184** | **Psi184** | **LASSO_ANTXXIX/3** | **Psilaster charcoti** | **Christopher L. Mah** |  | **2013** | **-63.80** | **-56.31** | **TipPen** | **554.4** |
| **Psi185** | **Psi185** | **LASSO_ANTXXIX/3** | **Psilaster charcoti** | **Christopher L. Mah** |  | **2013** | **-63.80** | **-56.31** | **TipPen** | **554.4** |
| **Bat030** | **Bat030** | **LASSO_ANTXXIX/3** | **Bathybiaster loripes** | **Christopher L. Mah** |  | **2013** | **-62.44** | **-56.29** | **Bransfield** | **438.5** |
| **Lys184** | **Lys184** | **LASSO_ANTXXIX/3** | **Lysasterias sp** | **Christopher L. Mah** |  | **2013** | **-62.44** | **-56.29** | **Bransfield** | **438.5** |
| **Bat107** | **Bat107** | **LASSO_ANTXXIX/3** | **Bathybiaster loripes** | **Christopher L. Mah** |  | **2013** | **-63.62** | **-56.15** | **TipPen** | **101.8** |
| **Lys130** | **Lys130** | **LASSO_ANTXXIX/3** | **Lysasterias heteractis** | **Christopher L. Mah** |  | **2013** | **-63.62** | **-56.15** | **TipPen** | **101.8** |
| **Dip217** | **Dip217** | **LASSO_ANTXXIX/3** | **Diplasterias brucei** | **Christopher L. Mah** |  | **2013** | **-63.85** | **-55.68** | **TipPen** | **342** |
| **Dip227** | **Dip227** | **LASSO_ANTXXIX/3** | **Diplasterias brucei** | **Christopher L. Mah** |  | **2013** | **-63.85** | **-55.68** | **TipPen** | **342** |
| **Dip228** | **Dip228** | **LASSO_ANTXXIX/3** | **Diplasterias brucei** | **Christopher L. Mah** |  | **2013** | **-63.85** | **-55.68** | **TipPen** | **342** |
| **Lys182** | **Lys182** | **LASSO_ANTXXIX/3** | **Lysasterias heteractis** | **Christopher L. Mah** |  | **2013** | **-63.85** | **-55.68** | **TipPen** | **342** |
| **Lys196** | **Lys196** | **LASSO_ANTXXIX/3** | **Lysasterias heteractis** | **Christopher L. Mah** |  | **2013** | **-63.85** | **-55.68** | **TipPen** | **342** |
| **Dip202** | **Dip202** | **LASSO_ANTXXIX/3** | **Diplasterias brucei** | **Christopher L. Mah** |  | **2013** | **-63.84** | **-55.62** | **TipPen** | **425** |
| **Dip216** | **Dip216** | **LASSO_ANTXXIX/3** | **Diplasterias brucei** | **Christopher L. Mah** |  | **2013** | **-63.17** | **-54.12** | **TipPen** | **227.1** |
| **Lys183** | **Lys183** | **LASSO_ANTXXIX/3** | **Lysasterias sp** | **Christopher L. Mah** |  | **2013** | **-63.17** | **-54.12** | **TipPen** | **227.1** |
| **Lys193** | **Lys193** | **LASSO_ANTXXIX/3** | **Lysasterias sp** | **Christopher L. Mah** |  | **2013** | **-63.17** | **-54.12** | **TipPen** | **227.1** |
| **Lys194** | **Lys194** | **LASSO_ANTXXIX/3** | **Lysasterias sp** | **Christopher L. Mah** |  | **2013** | **-63.17** | **-54.12** | **TipPen** | **227.1** |
| **Lys333** | **Lys333** | **LASSO_ANTXXIX/3** | **Lysasterias sp** | **Christopher L. Mah** |  | **2013** | **-63.17** | **-54.12** | **TipPen** | **227.1** |
| **Lys334** | **Lys334** | **LASSO_ANTXXIX/3** | **Lysasterias sp** | **Christopher L. Mah** |  | **2013** | **-63.17** | **-54.12** | **TipPen** | **227.1** |
| **Psi117** | **Psi117** | **LASSO_ANTXXIX/3** | **Psilaster charcoti** | **Christopher L. Mah** |  | **2013** | **-63.17** | **-54.12** | **TipPen** | **227.1** |
| **Psi118** | **Psi118** | **LASSO_ANTXXIX/3** | **Psilaster charcoti** | **Christopher L. Mah** |  | **2013** | **-63.17** | **-54.12** | **TipPen** | **227.1** |
| **Psi119** | **Psi119** | **LASSO_ANTXXIX/3** | **Psilaster charcoti** | **Christopher L. Mah** |  | **2013** | **-63.17** | **-54.12** | **TipPen** | **227.1** |
| **Psi130** | **Psi130** | **LASSO_ANTXXIX/3** | **Psilaster charcoti** | **Christopher L. Mah** |  | **2013** | **-63.17** | **-54.12** | **TipPen** | **227.1** |
| **Not035** | **Not035** | **JR15005** | **Notasterias sp** | **Camille Moreau** |  | **2016** | **-61.54** | **-47.13** | **Sork** | **772** |
| **Psi163** | **Psi163** | **JR15005** | **Psilaster charcoti** | **Camille Moreau** |  | **2016** | **-61.54** | **-47.13** | **Sork** | **772** |
| **Psi003** | **Psi003** | **JR15005** | **Psilaster charcoti** | **Camille Moreau** |  | **2016** | **-61.54** | **-46.94** | **Sork** | **522** |
| **Not036** | **Not036** | **JR15005** | **Notasterias sp** | **Camille Moreau** |  | **2016** | **-60.27** | **-46.88** | **Sork** | **459** |
| **Not390** | **Not390** | **JR15005** |  | **Camille Moreau** |  | **2016** | **-60.27** | **-46.88** | **Sork** | **459** |
| **Not444** | **Not444** | **JR15005** |  | **Camille Moreau** |  | **2016** | **-60.27** | **-46.88** | **Sork** | **459** |
| **Not445** | **Not445** | **JR15005** |  | **Camille Moreau** |  | **2016** | **-60.27** | **-46.88** | **Sork** | **459** |
| **Not446** | **Not446** | **JR15005** |  | **Camille Moreau** |  | **2016** | **-60.27** | **-46.88** | **Sork** | **459** |
| **Not447** | **Not447** | **JR15005** |  | **Camille Moreau** |  | **2016** | **-60.27** | **-46.88** | **Sork** | **459** |
| **Not448** | **Not448** | **JR15005** |  | **Camille Moreau** |  | **2016** | **-60.27** | **-46.88** | **Sork** | **459** |
| **Not449** | **Not449** | **JR15005** |  | **Camille Moreau** |  | **2016** | **-60.27** | **-46.88** | **Sork** | **459** |
| **Not451** | **Not451** | **JR15005** |  | **Camille Moreau** |  | **2016** | **-60.27** | **-46.88** | **Sork** | **459** |
| **Not452** | **Not452** | **JR15005** |  | **Camille Moreau** |  | **2016** | **-60.27** | **-46.88** | **Sork** | **459** |
| **Not453** | **Not453** | **JR15005** |  | **Camille Moreau** |  | **2016** | **-60.27** | **-46.88** | **Sork** | **459** |
| **Psi156** | **Psi156** | **JR15005** |  | **Camille Moreau** |  | **2016** | **-60.27** | **-46.88** | **Sork** | **459** |
| **Not454** | **Not454** | **JR15005** |  | **Camille Moreau** |  | **2016** | **-60.27** | **-46.88** | **Sork** | **459** |
| **Not455** | **Not455** | **JR15005** |  | **Camille Moreau** |  | **2016** | **-60.27** | **-46.88** | **Sork** | **459** |
| **Not457** | **Not457** | **JR15005** |  | **Camille Moreau** |  | **2016** | **-60.27** | **-46.88** | **Sork** | **459** |
| **Not458** | **Not458** | **JR15005** |  | **Camille Moreau** |  | **2016** | **-60.27** | **-46.88** | **Sork** | **459** |
| **Not010** | **Not010** | **JR15005** | **Notasterias sp** | **Camille Moreau** |  | **2016** | **-60.28** | **-46.87** | **Sork** | **484** |
| **Not011** | **Not011** | **JR15005** | **Notasterias sp** | **Camille Moreau** |  | **2016** | **-60.28** | **-46.87** | **Sork** | **484** |
| **Not012** | **Not012** | **JR15005** | **Notasterias sp** | **Camille Moreau** |  | **2016** | **-60.28** | **-46.87** | **Sork** | **484** |
| **Not013** | **Not013** | **JR15005** | **Notasterias sp** | **Camille Moreau** |  | **2016** | **-60.28** | **-46.87** | **Sork** | **484** |
| **Not033** | **Not033** | **JR15005** | **Notasterias sp** | **Camille Moreau** |  | **2016** | **-60.28** | **-46.87** | **Sork** | **484** |
| **Not042** | **Not042** | **JR15005** | **Notasterias sp** | **Camille Moreau** |  | **2016** | **-60.28** | **-46.87** | **Sork** | **484** |
| **Not043** | **Not043** | **JR15005** | **Notasterias sp** | **Camille Moreau** |  | **2016** | **-60.28** | **-46.87** | **Sork** | **484** |
| **Not044** | **Not044** | **JR15005** | **Notasterias sp** | **Camille Moreau** |  | **2016** | **-60.28** | **-46.87** | **Sork** | **484** |
| **Not060** | **Not060** | **JR15005** | **Notasterias sp** | **Camille Moreau** |  | **2016** | **-60.28** | **-46.87** | **Sork** | **484** |
| **Not061** | **Not061** | **JR15005** | **Notasterias sp** | **Camille Moreau** |  | **2016** | **-60.28** | **-46.87** | **Sork** | **484** |
| **Not062** | **Not062** | **JR15005** | **Notasterias sp** | **Camille Moreau** |  | **2016** | **-60.28** | **-46.87** | **Sork** | **484** |
| **Not063** | **Not063** | **JR15005** | **Notasterias sp** | **Camille Moreau** |  | **2016** | **-60.28** | **-46.87** | **Sork** | **484** |
| **Not064** | **Not064** | **JR15005** | **Notasterias sp** | **Camille Moreau** |  | **2016** | **-60.28** | **-46.87** | **Sork** | **484** |
| **Not065** | **Not065** | **JR15005** | **Notasterias sp** | **Camille Moreau** |  | **2016** | **-60.28** | **-46.87** | **Sork** | **484** |
| **Not066** | **Not066** | **JR15005** | **Notasterias sp** | **Camille Moreau** |  | **2016** | **-60.28** | **-46.87** | **Sork** | **484** |
| **Psi014** | **Psi014** | **JR15005** | **Psilaster charcoti** | **Camille Moreau** |  | **2016** | **-60.28** | **-46.87** | **Sork** | **484** |
| **Dip079** | **Dip079** | **JR144** | **Undet (cf diplasterias)** | **Camille Moreau** |  | **2006** | **-60.99** | **-46.83** | **Sork** | **507** |
| **Dip080** | **Dip080** | **JR144** | **Undet (cf diplasterias)** | **Camille Moreau** |  | **2006** | **-60.99** | **-46.83** | **Sork** | **507** |
| **Dip081** | **Dip081** | **JR144** | **Undet (cf diplasterias)** | **Camille Moreau** |  | **2006** | **-60.99** | **-46.83** | **Sork** | **507** |
| **Dip082** | **Dip082** | **JR144** | **Undet (cf diplasterias)** | **Camille Moreau** |  | **2006** | **-60.99** | **-46.83** | **Sork** | **507** |
| **Dip083** | **Dip083** | **JR144** | **Undet (cf diplasterias)** | **Camille Moreau** |  | **2006** | **-60.99** | **-46.83** | **Sork** | **507** |
| **Dip085** | **Dip085** | **JR144** | **Undet (cf diplasterias)** | **Camille Moreau** |  | **2006** | **-60.99** | **-46.83** | **Sork** | **507** |
| **Dip086** | **Dip086** | **JR144** | **Undet (cf diplasterias)** | **Camille Moreau** |  | **2006** | **-60.99** | **-46.83** | **Sork** | **507** |
| **Dip087** | **Dip087** | **JR144** | **Undet (cf diplasterias)** | **Camille Moreau** |  | **2006** | **-60.99** | **-46.83** | **Sork** | **507** |
| **Dip088** | **Dip088** | **JR144** | **Undet (cf diplasterias)** | **Camille Moreau** |  | **2006** | **-60.99** | **-46.83** | **Sork** | **507** |
| **Dip191** | **Dip191** | **JR144** | **Diplasterias brandti** | **Christopher L. Mah** |  | **2006** | **-60.99** | **-46.83** | **Sork** | **507** |
| **Not093** | **Not093** | **JR15005** | **Notasterias sp** | **Camille Moreau** |  | **2016** | **-60.32** | **-46.77** | **Sork** | **722** |
| **Not094** | **Not094** | **JR15005** | **Notasterias sp** | **Camille Moreau** |  | **2016** | **-60.32** | **-46.77** | **Sork** | **722** |
| **Not095** | **Not095** | **JR15005** | **Notasterias sp** | **Camille Moreau** |  | **2016** | **-60.32** | **-46.77** | **Sork** | **722** |
| **Not097** | **Not097** | **JR15005** | **Notasterias sp** | **Camille Moreau** |  | **2016** | **-60.32** | **-46.77** | **Sork** | **722** |
| **Not098** | **Not098** | **JR15005** | **Notasterias sp** | **Camille Moreau** |  | **2016** | **-60.32** | **-46.77** | **Sork** | **722** |
| **Not384** | **Not384** | **JR15005** |  | **Camille Moreau** |  | **2016** | **-60.32** | **-46.77** | **Sork** | **722** |
| **Not386** | **Not386** | **JR15005** |  | **Camille Moreau** |  | **2016** | **-60.32** | **-46.77** | **Sork** | **722** |
| **Not387** | **Not387** | **JR15005** |  | **Camille Moreau** |  | **2016** | **-60.32** | **-46.77** | **Sork** | **722** |
| **Not388** | **Not388** | **JR15005** |  | **Camille Moreau** |  | **2016** | **-60.32** | **-46.77** | **Sork** | **722** |
| **Not389** | **Not389** | **JR15005** |  | **Camille Moreau** |  | **2016** | **-60.32** | **-46.77** | **Sork** | **722** |
| **Psi024** | **Psi024** | **JR15005** |  | **Camille Moreau** |  | **2016** | **-60.32** | **-46.77** | **Sork** | **722** |
| **Psi025** | **Psi025** | **JR15005** |  | **Camille Moreau** |  | **2016** | **-60.32** | **-46.77** | **Sork** | **722** |
| **Psi026** | **Psi026** | **JR15005** |  | **Camille Moreau** |  | **2016** | **-60.32** | **-46.77** | **Sork** | **722** |
| **Psi157** | **Psi157** | **JR15005** |  | **Camille Moreau** |  | **2016** | **-60.32** | **-46.77** | **Sork** | **722** |
| **Psi158** | **Psi158** | **JR15005** |  | **Camille Moreau** |  | **2016** | **-60.32** | **-46.77** | **Sork** | **722** |
| **Psi159** | **Psi159** | **JR15005** |  | **Camille Moreau** |  | **2016** | **-60.32** | **-46.77** | **Sork** | **722** |
| **Not416** | **Not416** | **JR15005** |  | **Camille Moreau** |  | **2016** | **-60.32** | **-46.77** | **Sork** | **733** |
| **Not417** | **Not417** | **JR15005** |  | **Camille Moreau** |  | **2016** | **-60.32** | **-46.77** | **Sork** | **733** |
| **Not418** | **Not418** | **JR15005** |  | **Camille Moreau** |  | **2016** | **-60.32** | **-46.77** | **Sork** | **733** |
| **Not419** | **Not419** | **JR15005** |  | **Camille Moreau** |  | **2016** | **-60.32** | **-46.77** | **Sork** | **733** |
| **Not420** | **Not420** | **JR15005** |  | **Camille Moreau** |  | **2016** | **-60.32** | **-46.77** | **Sork** | **733** |
| **Not421** | **Not421** | **JR15005** |  | **Camille Moreau** |  | **2016** | **-60.32** | **-46.77** | **Sork** | **733** |
| **Not422** | **Not422** | **JR15005** |  | **Camille Moreau** |  | **2016** | **-60.32** | **-46.77** | **Sork** | **733** |
| **Not423** | **Not423** | **JR15005** |  | **Camille Moreau** |  | **2016** | **-60.32** | **-46.77** | **Sork** | **733** |
| **Not424** | **Not424** | **JR15005** |  | **Camille Moreau** |  | **2016** | **-60.32** | **-46.77** | **Sork** | **733** |
| **Not425** | **Not425** | **JR15005** |  | **Camille Moreau** |  | **2016** | **-60.32** | **-46.77** | **Sork** | **733** |
| **Not426** | **Not426** | **JR15005** |  | **Camille Moreau** |  | **2016** | **-60.32** | **-46.77** | **Sork** | **733** |
| **Not427** | **Not427** | **JR15005** |  | **Camille Moreau** |  | **2016** | **-60.32** | **-46.77** | **Sork** | **733** |
| **Not428** | **Not428** | **JR15005** |  | **Camille Moreau** |  | **2016** | **-60.32** | **-46.77** | **Sork** | **733** |
| **Not429** | **Not429** | **JR15005** |  | **Camille Moreau** |  | **2016** | **-60.32** | **-46.77** | **Sork** | **733** |
| **Not430** | **Not430** | **JR15005** |  | **Camille Moreau** |  | **2016** | **-60.32** | **-46.77** | **Sork** | **733** |
| **Not431** | **Not431** | **JR15005** |  | **Camille Moreau** |  | **2016** | **-60.32** | **-46.77** | **Sork** | **733** |
| **Not432** | **Not432** | **JR15005** |  | **Camille Moreau** |  | **2016** | **-60.32** | **-46.77** | **Sork** | **733** |
| **Not434** | **Not434** | **JR15005** |  | **Camille Moreau** |  | **2016** | **-60.32** | **-46.77** | **Sork** | **733** |
| **Not437** | **Not437** | **JR15005** |  | **Camille Moreau** |  | **2016** | **-60.32** | **-46.77** | **Sork** | **733** |
| **Not438** | **Not438** | **JR15005** |  | **Camille Moreau** |  | **2016** | **-60.32** | **-46.77** | **Sork** | **733** |
| **Not439** | **Not439** | **JR15005** |  | **Camille Moreau** |  | **2016** | **-60.32** | **-46.77** | **Sork** | **733** |
| **Not440** | **Not440** | **JR15005** |  | **Camille Moreau** |  | **2016** | **-60.32** | **-46.77** | **Sork** | **733** |
| **Not441** | **Not441** | **JR15005** |  | **Camille Moreau** |  | **2016** | **-60.32** | **-46.77** | **Sork** | **733** |
| **Not442** | **Not442** | **JR15005** |  | **Camille Moreau** |  | **2016** | **-60.32** | **-46.77** | **Sork** | **733** |
| **Not443** | **Not443** | **JR15005** |  | **Camille Moreau** |  | **2016** | **-60.32** | **-46.77** | **Sork** | **733** |
| **Psi166** | **Psi166** | **JR15005** |  | **Camille Moreau** |  | **2016** | **-60.32** | **-46.77** | **Sork** | **733** |
| **Psi167** | **Psi167** | **JR15005** |  | **Camille Moreau** |  | **2016** | **-60.32** | **-46.77** | **Sork** | **733** |
| **Psi168** | **Psi168** | **JR15005** |  | **Camille Moreau** |  | **2016** | **-60.32** | **-46.77** | **Sork** | **733** |
| **Psi169** | **Psi169** | **JR15005** |  | **Camille Moreau** |  | **2016** | **-60.32** | **-46.77** | **Sork** | **733** |
| **Lys032** | **Lys032** | **JR15005** |  | **Camille Moreau** |  | **2016** | **-60.32** | **-46.77** | **Sork** | **732** |
| **Not086** | **Not086** | **JR15005** | **Notasterias sp** | **Camille Moreau** |  | **2016** | **-60.32** | **-46.77** | **Sork** | **732** |
| **Not087** | **Not087** | **JR15005** | **Notasterias sp** | **Camille Moreau** |  | **2016** | **-60.32** | **-46.77** | **Sork** | **732** |
| **Not088** | **Not088** | **JR15005** | **Notasterias sp** | **Camille Moreau** |  | **2016** | **-60.32** | **-46.77** | **Sork** | **732** |
| **Not090** | **Not090** | **JR15005** | **Notasterias sp** | **Camille Moreau** |  | **2016** | **-60.32** | **-46.77** | **Sork** | **732** |
| **Not091** | **Not091** | **JR15005** | **Notasterias sp** | **Camille Moreau** |  | **2016** | **-60.32** | **-46.77** | **Sork** | **732** |
| **Not092** | **Not092** | **JR15005** | **Notasterias sp** | **Camille Moreau** |  | **2016** | **-60.32** | **-46.77** | **Sork** | **732** |
| **Psi021** | **Psi021** | **JR15005** |  | **Camille Moreau** |  | **2016** | **-60.32** | **-46.77** | **Sork** | **732** |
| **Psi022** | **Psi022** | **JR15005** |  | **Camille Moreau** |  | **2016** | **-60.32** | **-46.77** | **Sork** | **732** |
| **Psi023** | **Psi023** | **JR15005** |  | **Camille Moreau** |  | **2016** | **-60.32** | **-46.77** | **Sork** | **732** |
| **Und037** | **Und037** | **JR15005** |  | **Camille Moreau** |  | **2016** | **-60.32** | **-46.77** | **Sork** | **732** |
| **Psi162** | **Psi162** | **JR15005** |  | **Camille Moreau** |  | **2016** | **-60.22** | **-46.69** | **Sork** | **783** |
| **Bat001** | **Bat001** | **JR15005** | **Bathybiaster loripes** | **Camille Moreau** |  | **2016** | **-60.35** | **-46.68** | **Sork** | **465** |
| **Bat003** | **Bat003** | **JR15005** | **Bathybiaster loripes** | **Camille Moreau** |  | **2016** | **-60.35** | **-46.68** | **Sork** | **442** |
| **Dip110** | **Dip110** | **JR15005** | **Diplasterias** | **Camille Moreau** |  | **2016** | **-60.35** | **-46.68** | **Sork** | **442** |
| **Lys015** | **Lys015** | **JR15005** | **Lysasterias sp** | **Camille Moreau** |  | **2016** | **-60.35** | **-46.68** | **Sork** | **465** |
| **Lys020** | **Lys020** | **JR15005** | **Lysasterias sp** | **Camille Moreau** |  | **2016** | **-60.35** | **-46.68** | **Sork** | **442** |
| **Lys313** | **Lys313** | **JR15005** |  | **Camille Moreau** |  | **2016** | **-60.35** | **-46.68** | **Sork** | **465** |
| **Psi004** | **Psi004** | **JR15005** | **Psilaster charcoti** | **Camille Moreau** |  | **2016** | **-60.35** | **-46.68** | **Sork** | **465** |
| **Psi005** | **Psi005** | **JR15005** | **Psilaster charcoti** | **Camille Moreau** |  | **2016** | **-60.35** | **-46.68** | **Sork** | **465** |
| **Psi006** | **Psi006** | **JR15005** | **Psilaster charcoti** | **Camille Moreau** |  | **2016** | **-60.35** | **-46.68** | **Sork** | **465** |
| **Psi007** | **Psi007** | **JR15005** | **Psilaster charcoti** | **Camille Moreau** |  | **2016** | **-60.35** | **-46.68** | **Sork** | **465** |
| **Psi008** | **Psi008** | **JR15005** | **Psilaster charcoti** | **Camille Moreau** |  | **2016** | **-60.35** | **-46.68** | **Sork** | **465** |
| **Psi011** | **Psi011** | **JR15005** | **Psilaster charcoti** | **Camille Moreau** |  | **2016** | **-60.35** | **-46.68** | **Sork** | **442** |
| **Psi012** | **Psi012** | **JR15005** | **Psilaster charcoti** | **Camille Moreau** |  | **2016** | **-60.35** | **-46.68** | **Sork** | **442** |
| **Psi013** | **Psi013** | **JR15005** | **Psilaster charcoti** | **Camille Moreau** |  | **2016** | **-60.35** | **-46.68** | **Sork** | **442** |
| **Psi016** | **Psi016** | **JR15005** | **Psilaster charcoti** | **Camille Moreau** |  | **2016** | **-60.35** | **-46.68** | **Sork** | **442** |
| **Psi164** | **Psi164** | **JR15005** | **Psilaster charcoti** | **Camille Moreau** |  | **2016** | **-60.35** | **-46.68** | **Sork** | **465** |
| **Und003** | **Und003** | **JR15005** | **Undet** | **Camille Moreau** |  | **2016** | **-60.35** | **-46.68** | **Sork** | **465** |
| **Und006** | **Und006** | **JR15005** | **Undet** | **Camille Moreau** |  | **2016** | **-60.35** | **-46.68** | **Sork** | **465** |
| **Und007** | **Und007** | **JR15005** | **Undet** | **Camille Moreau** |  | **2016** | **-60.35** | **-46.68** | **Sork** | **465** |
| **Und008** | **Und008** | **JR15005** | **Undet** | **Camille Moreau** |  | **2016** | **-60.35** | **-46.68** | **Sork** | **465** |
| **Und009** | **Und009** | **JR15005** | **Undet** | **Camille Moreau** |  | **2016** | **-60.35** | **-46.68** | **Sork** | **465** |
| **Und010** | **Und010** | **JR15005** | **Undet** | **Camille Moreau** |  | **2016** | **-60.35** | **-46.68** | **Sork** | **465** |
| **Und012** | **Und012** | **JR15005** | **Undet** | **Camille Moreau** |  | **2016** | **-60.35** | **-46.68** | **Sork** | **442** |
| **Und014** | **Und014** | **JR15005** | **Undet** | **Camille Moreau** |  | **2016** | **-60.35** | **-46.68** | **Sork** | **442** |
| **Bat002** | **Bat002** | **JR15005** | **Bathybiaster loripes** | **Camille Moreau** |  | **2016** | **-60.35** | **-46.68** | **Sork** | **458** |
| **Dip045** | **Dip045** | **JR15005** | **Diplasterias sp** | **Camille Moreau** |  | **2016** | **-60.35** | **-46.68** | **Sork** | **458** |
| **Lys016** | **Lys016** | **JR15005** | **Lysasterias sp** | **Camille Moreau** |  | **2016** | **-60.35** | **-46.68** | **Sork** | **458** |
| **Lys017** | **Lys017** | **JR15005** | **Lysasterias sp** | **Camille Moreau** |  | **2016** | **-60.35** | **-46.68** | **Sork** | **458** |
| **Psi009** | **Psi009** | **JR15005** | **Psilaster charcoti** | **Camille Moreau** |  | **2016** | **-60.35** | **-46.68** | **Sork** | **458** |
| **Psi010** | **Psi010** | **JR15005** | **Psilaster charcoti** | **Camille Moreau** |  | **2016** | **-60.35** | **-46.68** | **Sork** | **458** |
| **Psi188** | **Psi188** | **JR144** | **Psilaster charcoti** | **Camille Moreau** |  | **2006** | **-60.82** | **-46.49** | **Sork** | **221** |
| **Dip373** | **Dip373** | **JR15005** | **Diplasterias** | **Camille Moreau** |  | **2016** | **-62.28** | **-45.00** | **Sork** | **726** |
| **Not391** | **Not391** | **JR15005** | **Notasterias sp** | **Camille Moreau** |  | **2016** | **-62.28** | **-45.00** | **Sork** | **726** |
| **Und039** | **Und039** | **JR15005** |  | **Camille Moreau** |  | **2016** | **-62.28** | **-45.00** | **Sork** | **726** |
| **Not045** | **Not045** | **JR15005** | **Notasterias sp** | **Camille Moreau** |  | **2016** | **-62.16** | **-44.99** | **Sork** | **524** |
| **Psi015** | **Psi015** | **JR15005** | **Psilaster charcoti** | **Camille Moreau** |  | **2016** | **-62.16** | **-44.99** | **Sork** | **524** |
| **Dip043** | **Dip043** | **JR15005** | **Diplasterias** | **Camille Moreau** |  | **2016** | **-62.17** | **-44.98** | **Sork** | **531** |
| **Not052** | **Not052** | **JR15005** | **Notasterias sp** | **Camille Moreau** |  | **2016** | **-60.47** | **-44.72** | **Sork** | **992** |
| **Not053** | **Not053** | **JR15005** | **Notasterias sp** | **Camille Moreau** |  | **2016** | **-60.47** | **-44.72** | **Sork** | **992** |
| **Not054** | **Not054** | **JR15005** | **Notasterias sp** | **Camille Moreau** |  | **2016** | **-60.47** | **-44.72** | **Sork** | **992** |
| **Not055** | **Not055** | **JR15005** | **Notasterias sp** | **Camille Moreau** |  | **2016** | **-60.47** | **-44.72** | **Sork** | **992** |
| **Not392** | **Not392** | **JR15005** |  | **Camille Moreau** |  | **2016** | **-60.47** | **-44.72** | **Sork** | **992** |
| **Not047** | **Not047** | **JR15005** | **Notasterias sp** | **Camille Moreau** |  | **2016** | **-60.46** | **-44.70** | **Sork** | **1062** |
| **Not048** | **Not048** | **JR15005** | **Notasterias sp** | **Camille Moreau** |  | **2016** | **-60.46** | **-44.70** | **Sork** | **1062** |
| **Not049** | **Not049** | **JR15005** | **Notasterias sp** | **Camille Moreau** |  | **2016** | **-60.46** | **-44.70** | **Sork** | **1062** |
| **Dip097** | **Dip097** | **JR15005** | **Diplasterias** | **Camille Moreau** |  | **2016** | **-60.46** | **-44.70** | **Sork** | **1028** |
| **Dip099** | **Dip099** | **JR15005** | **Diplasterias** | **Camille Moreau** |  | **2016** | **-60.46** | **-44.70** | **Sork** | **1028** |
| **Dip111** | **Dip111** | **JR15005** | **Notasterias sp** | **Camille Moreau** |  | **2016** | **-60.46** | **-44.70** | **Sork** | **1028** |
| **Dip112** | **Dip112** | **JR15005** | **Notasterias sp** | **Camille Moreau** |  | **2016** | **-60.46** | **-44.70** | **Sork** | **1028** |
| **Not067** | **Not067** | **JR15005** | **Notasterias sp** | **Camille Moreau** |  | **2016** | **-60.46** | **-44.70** | **Sork** | **1028** |
| **Not068** | **Not068** | **JR15005** | **Notasterias sp** | **Camille Moreau** |  | **2016** | **-60.46** | **-44.70** | **Sork** | **1028** |
| **Not069** | **Not069** | **JR15005** | **Notasterias sp** | **Camille Moreau** |  | **2016** | **-60.46** | **-44.70** | **Sork** | **1028** |
| **Not070** | **Not070** | **JR15005** | **Notasterias sp** | **Camille Moreau** |  | **2016** | **-60.46** | **-44.70** | **Sork** | **1028** |
| **Not071** | **Not071** | **JR15005** | **Notasterias sp** | **Camille Moreau** |  | **2016** | **-60.46** | **-44.70** | **Sork** | **1028** |
| **Not072** | **Not072** | **JR15005** | **Notasterias sp** | **Camille Moreau** |  | **2016** | **-60.46** | **-44.70** | **Sork** | **1028** |
| **Not073** | **Not073** | **JR15005** | **Notasterias sp** | **Camille Moreau** |  | **2016** | **-60.46** | **-44.70** | **Sork** | **1028** |
| **Not075** | **Not075** | **JR15005** | **Notasterias sp** | **Camille Moreau** |  | **2016** | **-60.46** | **-44.70** | **Sork** | **1028** |
| **Not077** | **Not077** | **JR15005** | **Notasterias sp** | **Camille Moreau** |  | **2016** | **-60.46** | **-44.70** | **Sork** | **1028** |
| **Not078** | **Not078** | **JR15005** | **Notasterias sp** | **Camille Moreau** |  | **2016** | **-60.46** | **-44.70** | **Sork** | **1028** |
| **Not079** | **Not079** | **JR15005** | **Notasterias sp** | **Camille Moreau** |  | **2016** | **-60.46** | **-44.70** | **Sork** | **1028** |
| **Not080** | **Not080** | **JR15005** | **Notasterias sp** | **Camille Moreau** |  | **2016** | **-60.46** | **-44.70** | **Sork** | **1028** |
| **Not081** | **Not081** | **JR15005** | **Notasterias sp** | **Camille Moreau** |  | **2016** | **-60.46** | **-44.70** | **Sork** | **1028** |
| **Not082** | **Not082** | **JR15005** | **Notasterias sp** | **Camille Moreau** |  | **2016** | **-60.46** | **-44.70** | **Sork** | **1028** |
| **Not083** | **Not083** | **JR15005** | **Notasterias sp** | **Camille Moreau** |  | **2016** | **-60.46** | **-44.70** | **Sork** | **1028** |
| **Not084** | **Not084** | **JR15005** | **Notasterias sp** | **Camille Moreau** |  | **2016** | **-60.46** | **-44.70** | **Sork** | **1028** |
| **Not085** | **Not085** | **JR15005** | **Notasterias sp** | **Camille Moreau** |  | **2016** | **-60.46** | **-44.70** | **Sork** | **1028** |
| **Dip100** | **Dip100** | **JR15005** |  | **Camille Moreau** |  | **2016** | **-62.33** | **-44.54** | **Sork** | **969** |
| **Dip102** | **Dip102** | **JR15005** |  | **Camille Moreau** |  | **2016** | **-62.33** | **-44.54** | **Sork** | **969** |
| **Dip103** | **Dip103** | **JR15005** |  | **Camille Moreau** |  | **2016** | **-62.33** | **-44.54** | **Sork** | **969** |
| **Dip105** | **Dip105** | **JR15005** |  | **Camille Moreau** |  | **2016** | **-62.33** | **-44.54** | **Sork** | **969** |
| **Dip106** | **Dip106** | **JR15005** |  | **Camille Moreau** |  | **2016** | **-62.33** | **-44.54** | **Sork** | **969** |
| **Dip114** | **Dip114** | **JR15005** | **Diplasterias** | **Camille Moreau** |  | **2016** | **-62.33** | **-44.54** | **Sork** | **969** |
| **Dip115** | **Dip115** | **JR15005** | **Diplasterias** | **Camille Moreau** |  | **2016** | **-62.33** | **-44.54** | **Sork** | **969** |
| **Dip116** | **Dip116** | **JR15005** | **Diplasterias** | **Camille Moreau** |  | **2016** | **-62.33** | **-44.54** | **Sork** | **969** |
| **Not051** | **Not051** | **JR15005** | **Notasterias sp** | **Camille Moreau** |  | **2016** | **-62.33** | **-44.54** | **Sork** | **969** |
| **Dip108** | **Dip108** | **JR15005** |  | **Camille Moreau** |  | **2016** | **-60.48** | **-44.42** | **Sork** | **790** |
| **Dip109** | **Dip109** | **JR15005** |  | **Camille Moreau** |  | **2016** | **-60.48** | **-44.42** | **Sork** | **790** |
| **Not057** | **Not057** | **JR15005** | **Notasterias sp** | **Camille Moreau** |  | **2016** | **-60.48** | **-44.42** | **Sork** | **790** |
| **Not058** | **Not058** | **JR15005** | **Notasterias sp** | **Camille Moreau** |  | **2016** | **-60.48** | **-44.42** | **Sork** | **790** |
| **Not059** | **Not059** | **JR15005** | **Notasterias sp** | **Camille Moreau** |  | **2016** | **-60.48** | **-44.42** | **Sork** | **790** |
| **Not393** | **Not393** | **JR15005** |  | **Camille Moreau** |  | **2016** | **-60.48** | **-44.42** | **Sork** | **790** |
| **Psi018** | **Psi018** | **JR15005** |  | **Camille Moreau** |  | **2016** | **-60.48** | **-44.42** | **Sork** | **790** |
| **Psi019** | **Psi019** | **JR15005** |  | **Camille Moreau** |  | **2016** | **-60.48** | **-44.42** | **Sork** | **790** |
| **Psi020** | **Psi020** | **JR15005** |  | **Camille Moreau** |  | **2016** | **-60.48** | **-44.42** | **Sork** | **790** |
| **Dip107** | **Dip107** | **JR15005** |  | **Camille Moreau** |  | **2016** | **-60.48** | **-44.42** | **Sork** | **773** |
| **Psi017** | **Psi017** | **JR15005** |  | **Camille Moreau** |  | **2016** | **-60.48** | **-44.42** | **Sork** | **773** |
| **Psi002** | **Psi002** | **JR15005** | **Psilaster charcoti** | **Camille Moreau** |  | **2016** | **-60.72** | **-43.03** | **Sork** | **1014** |
| **Not031** | **Not031** | **JR15005** | **Notasterias sp** | **Camille Moreau** |  | **2016** | **-60.73** | **-43.00** | **Sork** | **909** |
| **Not032** | **Not032** | **JR15005** | **Notasterias sp** | **Camille Moreau** |  | **2016** | **-60.73** | **-43.00** | **Sork** | **909** |
| **Not046** | **Not046** | **JR15005** | **Notasterias sp** | **Camille Moreau** |  | **2016** | **-60.76** | **-42.97** | **Sork** | **508** |
| **Psi001** | **Psi001** | **JR15005** | **Psilaster charcoti** | **Camille Moreau** |  | **2016** | **-60.67** | **-42.51** | **Sork** | **493** |
| **Not028** | **Not028** | **JR15005** | **Notasterias sp** | **Camille Moreau** |  | **2016** | **-60.67** | **-42.50** | **Sork** | **512** |
| **Not408** | **Not408** | **PS96_ANTXXXI/2** | **Notasterias sp** | **Camille Moreau** |  | **2016** | **-75.68** | **-42.47** | **WS** | **388.5** |
| **Not399** | **Not399** | **PS96_ANTXXXI/2** | **Notasterias sp** | **Camille Moreau** |  | **2016** | **-75.67** | **-42.44** | **WS** | **389.2** |
| **Not400** | **Not400** | **PS96_ANTXXXI/2** | **Notasterias sp** | **Camille Moreau** |  | **2016** | **-75.67** | **-42.44** | **WS** | **389.2** |
| **Not401** | **Not401** | **PS96_ANTXXXI/2** | **Notasterias sp** | **Camille Moreau** |  | **2016** | **-75.67** | **-42.44** | **WS** | **389.2** |
| **Not402** | **Not402** | **PS96_ANTXXXI/2** | **Notasterias sp** | **Camille Moreau** |  | **2016** | **-75.67** | **-42.44** | **WS** | **389.2** |
| **Not403** | **Not403** | **PS96_ANTXXXI/2** | **Notasterias sp** | **Camille Moreau** |  | **2016** | **-75.67** | **-42.44** | **WS** | **389.2** |
| **Not404** | **Not404** | **PS96_ANTXXXI/2** | **Notasterias sp** | **Camille Moreau** |  | **2016** | **-75.67** | **-42.44** | **WS** | **389.2** |
| **Not405** | **Not405** | **PS96_ANTXXXI/2** | **Notasterias sp** | **Camille Moreau** |  | **2016** | **-75.67** | **-42.44** | **WS** | **389.2** |
| **Not406** | **Not406** | **PS96_ANTXXXI/2** | **Notasterias sp** | **Camille Moreau** |  | **2016** | **-75.67** | **-42.44** | **WS** | **389.2** |
| **Not407** | **Not407** | **PS96_ANTXXXI/2** | **Notasterias sp** | **Camille Moreau** |  | **2016** | **-75.67** | **-42.44** | **WS** | **389.2** |
| **Und023** | **Und023** | **JR262** | **Undet** | **Camille Moreau** |  | **2011** | **-53.60** | **-41.21** | **Shag** | **130** |
| **Dip325c** | **Dip325c** | **POKER II** | **Diplasterias meridionalis** | **Christopher L. Mah** |  | **2011** | **-53.83** | **-41.00** | **Ker** | **160** |
| **Psi211** | **Psi211** | **JR262** | **Psilaster charcoti** | **Camille Moreau** |  | **2011** | **-54.40** | **-37.48** | **SG** | **325** |
| **Psi212** | **Psi212** | **JR262** | **Psilaster charcoti** | **Camille Moreau** |  | **2011** | **-54.40** | **-37.48** | **SG** | **325** |
| **Bat255** | **Bat255** | **JR262** | **Bathybiaster loripes** | **Camille Moreau** |  | **2011** | **-54.40** | **-37.37** | **SG** | **160** |
| **Psi209** | **Psi209** | **JR262** | **Psilaster charcoti** | **Camille Moreau** |  | **2011** | **-54.40** | **-37.37** | **SG** | **160** |
| **Psi210** | **Psi210** | **JR262** | **Psilaster charcoti** | **Camille Moreau** |  | **2011** | **-54.39** | **-37.26** | **SG** | **280** |
| **Und016** | **Und016** | **JR287SG** | **Undet (juv )** | **Camille Moreau** |  | **2013** | **-54.94** | **-35.98** | **SG** | **245** |
| **Dip089** | **Dip089** | **JR287SG** | **Undet (cf diplasterias)** | **Camille Moreau** |  | **2013** | **-54.94** | **-35.97** | **SG** | **245** |
| **Lys024** | **Lys024** | **JR287SG** | **Undet** | **Camille Moreau** |  | **2013** | **-54.94** | **-35.97** | **SG** | **245** |
| **Lys026** | **Lys026** | **JR262** | **Undet** | **Camille Moreau** |  | **2011** | **-55.17** | **-35.48** | **SG** | **125** |
| **Lys027** | **Lys027** | **JR262** | **Undet** | **Camille Moreau** |  | **2011** | **-55.17** | **-35.48** | **SG** | **125** |
| **Und020** | **Und020** | **JR262** | **Undet** | **Camille Moreau** |  | **2011** | **-55.17** | **-35.48** | **SG** | **125** |
| **Dip388** | **Dip388** | **PS96_ANTXXXI/2** | **Undet** | **Camille Moreau** |  | **2016** | **-74.94** | **-32.45** | **WS** | **621.3** |
| **Dip390** | **Dip390** | **PS96_ANTXXXI/2** | **Undet** | **Camille Moreau** |  | **2016** | **-74.94** | **-32.45** | **WS** | **621.3** |
| **Not409** | **Not409** | **PS96_ANTXXXI/2** | **Notasterias sp** | **Camille Moreau** |  | **2016** | **-74.94** | **-32.45** | **WS** | **621.3** |
| **Not396** | **Not396** | **PS96_ANTXXXI/2** | **Notasterias sp** | **Camille Moreau** |  | **2016** | **-75.63** | **-31.83** | **WS** | **736** |
| **Not397** | **Not397** | **PS96_ANTXXXI/2** | **Notasterias sp** | **Camille Moreau** |  | **2016** | **-75.63** | **-31.83** | **WS** | **736** |
| **Not398** | **Not398** | **PS96_ANTXXXI/2** | **Notasterias sp** | **Camille Moreau** |  | **2016** | **-75.63** | **-31.83** | **WS** | **736** |
| **Bat216** | **Bat216** | **PS96_ANTXXXI/2** | **Bathybiaster loripes** | **Camille Moreau** |  | **2016** | **-76.19** | **-30.05** | **WS** | **396** |
| **Bat217** | **Bat217** | **PS96_ANTXXXI/2** | **Bathybiaster loripes** | **Camille Moreau** |  | **2016** | **-76.19** | **-30.05** | **WS** | **396** |
| **Dip381** | **Dip381** | **PS96_ANTXXXI/2** | **Diplasterias sp** | **Camille Moreau** |  | **2016** | **-76.27** | **-29.12** | **WS** | **290** |
| **Dip437** | **Dip437** | **ACE_2017** |  | **Camille Moreau** |  | **2017** | **-59.47** | **-27.33** | **Ssand** |  |
| **Dip438** | **Dip438** | **ACE_2017** |  | **Camille Moreau** |  | **2017** | **-59.47** | **-27.33** | **Ssand** |  |
| **Dip439** | **Dip439** | **ACE_2017** |  | **Camille Moreau** |  | **2017** | **-59.47** | **-27.33** | **Ssand** |  |
| **Dip441** | **Dip441** | **ACE_2017** |  | **Camille Moreau** |  | **2017** | **-59.47** | **-27.33** | **Ssand** |  |
| **Dip443** | **Dip443** | **ACE_2017** |  | **Camille Moreau** |  | **2017** | **-59.47** | **-27.29** | **Ssand** |  |
| **Dip445** | **Dip445** | **ACE_2017** |  | **Camille Moreau** |  | **2017** | **-59.47** | **-27.29** | **Ssand** |  |
| **Psi228** | **Psi228** | **ACE_2017** | **Psilaster charcoti** | **Camille Moreau** |  | **2017** | **-59.47** | **-27.29** | **Ssand** | **132** |
| **Dip433** | **Dip433** | **ACE_2017** |  | **Camille Moreau** |  | **2017** | **-59.47** | **-27.29** | **Ssand** |  |
| **Dip435** | **Dip435** | **ACE_2017** |  | **Camille Moreau** |  | **2017** | **-59.47** | **-27.29** | **Ssand** |  |
| **Dip436** | **Dip436** | **ACE_2017** |  | **Camille Moreau** |  | **2017** | **-59.47** | **-27.29** | **Ssand** |  |
| **Bat256** | **Bat256** | **ARGOS** | **Bathybiaster loripes** | **Camille Moreau** |  | **2009** | **-59.53** | **-27.17** | **Ssand** | **993** |
| **Bat257** | **Bat257** | **ARGOS** | **Bathybiaster loripes** | **Camille Moreau** |  | **2009** | **-59.53** | **-27.17** | **Ssand** | **993** |
| **Bat258** | **Bat258** | **ARGOS** | **Bathybiaster loripes** | **Camille Moreau** |  | **2009** | **-59.53** | **-27.17** | **Ssand** | **993** |
| **Bat259** | **Bat259** | **ARGOS** | **Bathybiaster loripes** | **Camille Moreau** |  | **2009** | **-59.53** | **-27.17** | **Ssand** | **993** |
| **Bat260** | **Bat260** | **ARGOS** | **Bathybiaster loripes** | **Camille Moreau** |  | **2009** | **-59.53** | **-27.17** | **Ssand** | **993** |
| **Bat261** | **Bat261** | **ARGOS** | **Bathybiaster loripes** | **Camille Moreau** |  | **2009** | **-59.53** | **-27.17** | **Ssand** | **993** |
| **Bat262** | **Bat262** | **ARGOS** | **Bathybiaster loripes** | **Camille Moreau** |  | **2009** | **-59.53** | **-27.17** | **Ssand** | **993** |
| **Bat267** | **Bat267** | **ARGOS** | **Bathybiaster loripes** | **Camille Moreau** |  | **2009** | **-59.53** | **-27.17** | **Ssand** | **993** |
| **Psi193** | **Psi193** | **ARGOS** | **Psilaster charcoti** | **Camille Moreau** |  | **2009** | **-59.53** | **-27.17** | **Ssand** | **993** |
| **Psi194** | **Psi194** | **ARGOS** | **Psilaster charcoti** | **Camille Moreau** |  | **2009** | **-59.53** | **-27.17** | **Ssand** | **993** |
| **Psi195** | **Psi195** | **ARGOS** | **Psilaster charcoti** | **Camille Moreau** |  | **2009** | **-59.53** | **-27.17** | **Ssand** | **993** |
| **Lys348** | **Lys348** | **ACE_2017** | **Lysasterias sp** | **Camille Moreau** |  | **2017** | **-57.16** | **-26.79** | **Ssand** | **326** |
| **Not507** | **Not507** | **ACE_2017** | **Notasterias sp** | **Camille Moreau** |  | **2017** | **-57.15** | **-26.74** | **Ssand** | **277** |
| **Lys314** | **Lys314** | **PS96_ANTXXXI/2** | **Undet** | **Camille Moreau** |  | **2016** | **-72.30** | **-16.85** | **WS** | **878** |
| **Not394** | **Not394** | **PS96_ANTXXXI/2** | **Notasterias sp** | **Camille Moreau** |  | **2016** | **-72.30** | **-16.85** | **WS** | **878** |
| **Not395** | **Not395** | **PS96_ANTXXXI/2** | **Notasterias sp** | **Camille Moreau** |  | **2016** | **-72.30** | **-16.85** | **WS** | **878** |
| **Not414** | **Not414** | **PS96_ANTXXXI/2** | **Notasterias sp** | **Camille Moreau** |  | **2016** | **-72.30** | **-16.85** | **WS** | **878** |
| **Dip384** | **Dip384** | **PS96_ANTXXXI/2** | **Diplasterias sp** | **Camille Moreau** |  | **2015** | **-70.89** | **-11.13** | **WS** | **302** |
| **Not412** | **Not412** | **PS96_ANTXXXI/2** | **Notasterias sp** | **Camille Moreau** |  | **2015** | **-70.89** | **-11.13** | **WS** | **302** |
| **Psi165** | **Psi165** | **PS96_ANTXXXI/2** | **Bathybiaster loripes** | **Camille Moreau** |  | **2015** | **-70.89** | **-11.13** | **WS** | **302** |
| **Not410** | **Not410** | **PS96_ANTXXXI/2** | **Notasterias sp** | **Camille Moreau** |  | **2015** | **-70.89** | **-11.13** | **WS** | **302** |
| **Not002** | **Not002** | **ANDEEP-SYSTCO** | **Notasterias sp** | **Camille Moreau** |  | **2008** | **-70.40** | **-8.33** | **WS** | **602** |
| **Not505** | **Not505** | **ACE_2017** | **Notasterias sp** | **Camille Moreau** |  | **2017** | **-54.42** | **3.52** | **Bouv** | **327** |
| **Psi230** | **Psi230** | **ACE_2017** | **Psilaster charcoti** | **Camille Moreau** |  | **2017** | **-54.42** | **3.52** | **Bouv** | **327** |
| **Psi235** | **Psi235** | **ACE_2017** | **Psilaster charcoti** | **Camille Moreau** |  | **2017** | **-54.42** | **3.52** | **Bouv** | **327** |
| **Not508** | **Not508** | **ACE_2017** | **Notasterias sp** | **Camille Moreau** |  | **2017** | **-54.42** | **3.53** | **Bouv** | **399** |
| **Not509** | **Not509** | **ACE_2017** | **Notasterias sp** | **Camille Moreau** |  | **2017** | **-54.42** | **3.53** | **Bouv** | **399** |
| **Psi231** | **Psi231** | **ACE_2017** | **Psilaster charcoti** | **Camille Moreau** |  | **2017** | **-54.42** | **3.53** | **Bouv** | **399** |
| **Psi232** | **Psi232** | **ACE_2017** | **Psilaster charcoti** | **Camille Moreau** |  | **2017** | **-54.42** | **3.53** | **Bouv** | **399** |
| **Dip440** | **Dip440** | **ACE_2017** |  | **Camille Moreau** |  | **2017** | **-54.42** | **3.54** | **Bouv** |  |
| **Dip121** | **Dip121** | **Proteker I** | **Undet** | **Camille Moreau** |  | **2011** | **-48.56** | **69.02** | **Ker** | **5** |
| **Dip123** | **Dip123** | **Proteker II** | **Undet** | **Camille Moreau** |  | **2013** | **-49.36** | **69.14** | **Ker** | **42** |
| **Dip125** | **Dip125** | **Proteker II** | **Undet** | **Camille Moreau** |  | **2013** | **-49.36** | **69.14** | **Ker** | **42** |
| **Und042** | **Und042** | **Proteker I** | **Undet** | **Camille Moreau** |  | **2011** | **-49.32** | **69.53** | **Ker** | **15** |
| **Dip119** | **Dip119** | **Proteker II** | **Undet** | **Camille Moreau** |  | **2013** | **-49.32** | **69.53** | **Ker** | **18** |
| **Dip120** | **Dip120** | **Proteker II** | **Undet** | **Camille Moreau** |  | **2013** | **-49.32** | **69.53** | **Ker** | **18** |
| **Bat004** | **Bat004** | **Proteker II** | **Undet** | **Camille Moreau** |  | **2013** | **-49.23** | **69.56** | **Ker** | **17** |
| **Dip124** | **Dip124** | **Proteker II** | **Undet** | **Camille Moreau** |  | **2013** | **-49.23** | **69.56** | **Ker** | **17** |
| **Dip128** | **Dip128** | **Proteker III** | **Diplasterias** | **Camille Moreau** |  |  | **-49.39** | **70.18** | **Ker** | **50** |
| **Dip431** | **Dip431** | **ACE_2017** |  | **Camille Moreau** |  | **2017** | **-51.13** | **71.84** | **Ker** |  |
| **Dip131** | **Dip131** | **REVOLTA1** | **Diplasterias brucei** | **Christopher L. Mah** |  | **2010** | **-66.66** | **139.80** | **AdL** | **90** |
| **Dip132** | **Dip132** | **REVOLTA1** | **Diplasterias brucei** | **Christopher L. Mah** |  | **2010** | **-66.66** | **139.80** | **AdL** | **90** |
| **Dip133** | **Dip133** | **REVOLTA1** | **Diplasterias brucei** | **Christopher L. Mah** |  | **2010** | **-66.64** | **139.83** | **AdL** | **130** |
| **Lys042** | **Lys042** | **REVOLTA1** | **Lysasterias cf. lactea** | **Christopher L. Mah** |  | **2010** | **-66.66** | **139.87** | **AdL** | **104** |
| **Not160** | **Not160** | **REVOLTA1** | **Notasterias cf. stolophora** | **Christopher L. Mah** |  | **2010** | **-66.66** | **139.87** | **AdL** | **104** |
| **Psi032** | **Psi032** | **REVOLTA1** | **Psilaster charcoti** | **Christopher L. Mah** |  | **2010** | **-66.66** | **139.87** | **AdL** |  |
| **Not271** | **Not271** | **REVOLTA2** | **Notasterias armata** | **Christopher L. Mah** |  | **2011** | **-66.65** | **139.87** | **AdL** |  |
| **Dip271** | **Dip271** | **REVOLTA2** | **Diplasterias brucei** | **Christopher L. Mah** |  | **2011** | **-66.68** | **139.88** | **AdL** | **27** |
| **Lys277** | **Lys277** | **REVOLTA2** | **Lysasterias cf. lactea** | **Christopher L. Mah** |  | **2011** | **-66.68** | **139.88** | **AdL** | **27** |
| **Lys279** | **Lys279** | **REVOLTA2** | **Lysasterias cf. lactea** | **Christopher L. Mah** |  | **2011** | **-66.68** | **139.88** | **AdL** | **27** |
| **Lys280** | **Lys280** | **REVOLTA2** | **Lysasterias cf. lactea** | **Christopher L. Mah** |  | **2011** | **-66.68** | **139.88** | **AdL** | **27** |
| **Dip136** | **Dip136** | **REVOLTA1** | **Diplasterias brucei** | **Christopher L. Mah** |  | **2010** | **-66.68** | **139.89** | **AdL** | **52** |
| **Dip137** | **Dip137** | **REVOLTA1** | **Diplasterias brucei** | **Christopher L. Mah** |  | **2010** | **-66.68** | **139.89** | **AdL** | **52** |
| **Dip138** | **Dip138** | **REVOLTA1** | **Diplasterias brucei** | **Christopher L. Mah** |  | **2010** | **-66.68** | **139.89** | **AdL** | **52** |
| **Dip139** | **Dip139** | **REVOLTA1** | **Diplasterias brucei** | **Christopher L. Mah** |  | **2010** | **-66.68** | **139.89** | **AdL** | **52** |
| **Dip140** | **Dip140** | **REVOLTA1** | **Diplasterias brucei** | **Christopher L. Mah** |  | **2010** | **-66.68** | **139.89** | **AdL** | **52** |
| **Not161** | **Not161** | **REVOLTA1** | **Diplasterias brucei** | **Christopher L. Mah** |  | **2010** | **-66.68** | **139.89** | **AdL** |  |
| **Psi029** | **Psi029** | **REVOLTA1** | **Psilaster charcoti** | **Christopher L. Mah** |  | **2010** | **-66.68** | **139.89** | **AdL** |  |
| **Dip143** | **Dip143** | **REVOLTA1** | **Diplasterias brucei** | **Christopher L. Mah** |  | **2010** | **-66.66** | **139.92** | **AdL** | **44** |
| **Bat007** | **Bat007** | **REVOLTA1** | **Bathybiaster loripes** | **Christopher L. Mah** |  | **2010** | **-66.67** | **139.92** | **AdL** |  |
| **Bat008** | **Bat008** | **REVOLTA1** | **Bathybiaster loripes** | **Christopher L. Mah** |  | **2010** | **-66.67** | **139.92** | **AdL** |  |
| **Bat009** | **Bat009** | **REVOLTA1** | **Bathybiaster loripes** | **Christopher L. Mah** |  | **2010** | **-66.67** | **139.92** | **AdL** |  |
| **Lys045** | **Lys045** | **REVOLTA1** | **Lysasterias cf. lactea** | **Christopher L. Mah** |  | **2010** | **-66.67** | **139.92** | **AdL** | **53** |
| **Lys046** | **Lys046** | **REVOLTA1** | **Lysasterias cf. lactea** | **Christopher L. Mah** |  | **2010** | **-66.67** | **139.92** | **AdL** | **53** |
| **Not150** | **Not150** | **REVOLTA1** | **Notasterias armata** | **Christopher L. Mah** |  | **2010** | **-66.67** | **139.92** | **AdL** |  |
| **Psi036** | **Psi036** | **REVOLTA1** | **Psilaster charcoti** | **Christopher L. Mah** |  | **2010** | **-66.67** | **139.92** | **AdL** |  |
| **Psi037** | **Psi037** | **REVOLTA1** | **Psilaster charcoti** | **Christopher L. Mah** |  | **2010** | **-66.67** | **139.92** | **AdL** |  |
| **Dip142** | **Dip142** | **REVOLTA1** | **Diplasterias brucei** | **Christopher L. Mah** |  | **2010** | **-66.67** | **139.92** | **AdL** | **21** |
| **Lys043** | **Lys043** | **REVOLTA1** | **Lysasterias cf. lactea** | **Christopher L. Mah** |  | **2010** | **-66.67** | **139.92** | **AdL** | **20** |
| **Lys044** | **Lys044** | **REVOLTA1** | **Lysasterias cf. lactea** | **Christopher L. Mah** |  | **2010** | **-66.67** | **139.92** | **AdL** | **20** |
| **Dip254** | **Dip254** | **REVOLTA2** | **Diplasterias brandti** | **Christopher L. Mah** |  | **2011** | **-66.63** | **139.92** | **AdL** | **150** |
| **Dip134** | **Dip134** | **REVOLTA1** | **Diplasterias brucei** | **Christopher L. Mah** |  | **2010** | **-66.69** | **139.93** | **AdL** | **31** |
| **Lys034** | **Lys034** | **REVOLTA1** | **Lysasterias cf. lactea** | **Christopher L. Mah** |  | **2010** | **-66.69** | **139.93** | **AdL** | **31** |
| **Lys035** | **Lys035** | **REVOLTA1** | **Lysasterias cf. lactea** | **Christopher L. Mah** |  | **2010** | **-66.69** | **139.93** | **AdL** | **31** |
| **Not151** | **Not151** | **REVOLTA1** | **Notasterias armata** | **Christopher L. Mah** |  | **2010** | **-66.69** | **139.93** | **AdL** |  |
| **Not152** | **Not152** | **REVOLTA1** | **Notasterias armata** | **Christopher L. Mah** |  | **2010** | **-66.69** | **139.93** | **AdL** |  |
| **Not153** | **Not153** | **REVOLTA1** | **Notasterias armata** | **Christopher L. Mah** |  | **2010** | **-66.69** | **139.93** | **AdL** |  |
| **Not154** | **Not154** | **REVOLTA1** | **Notasterias armata** | **Christopher L. Mah** |  | **2010** | **-66.69** | **139.93** | **AdL** |  |
| **Psi030** | **Psi030** | **REVOLTA1** | **Psilaster charcoti** | **Christopher L. Mah** |  | **2010** | **-66.69** | **139.93** | **AdL** |  |
| **Psi031** | **Psi031** | **REVOLTA1** | **Psilaster charcoti** | **Christopher L. Mah** |  | **2010** | **-66.69** | **139.93** | **AdL** |  |
| **Psi147** | **Psi147** | **REVOLTA2** | **Psilaster charcoti** | **Christopher L. Mah** |  | **2011** | **-66.67** | **139.93** | **AdL** |  |
| **Dip259** | **Dip259** | **REVOLTA2** | **Diplasterias brucei** | **Christopher L. Mah** |  | **2011** | **-66.69** | **139.95** | **AdL** | **33** |
| **Dip261** | **Dip261** | **REVOLTA2** | **Diplasterias brucei** | **Christopher L. Mah** |  | **2011** | **-66.69** | **139.95** | **AdL** | **33** |
| **Dip262** | **Dip262** | **REVOLTA2** | **Diplasterias brucei** | **Christopher L. Mah** |  | **2011** | **-66.69** | **139.95** | **AdL** | **33** |
| **Lys284** | **Lys284** | **REVOLTA2** | **Lysasterias cf. lactea** | **Christopher L. Mah** |  | **2011** | **-66.69** | **139.95** | **AdL** | **34** |
| **Lys285** | **Lys285** | **REVOLTA2** | **Lysasterias cf. lactea** | **Christopher L. Mah** |  | **2011** | **-66.69** | **139.95** | **AdL** | **34** |
| **Not261** | **Not261** | **REVOLTA2** | **Notasterias armata** | **Christopher L. Mah** |  | **2011** | **-66.69** | **139.95** | **AdL** |  |
| **Not262** | **Not262** | **REVOLTA2** | **Notasterias armata** | **Christopher L. Mah** |  | **2011** | **-66.69** | **139.95** | **AdL** |  |
| **Not263** | **Not263** | **REVOLTA2** | **Notasterias armata** | **Christopher L. Mah** |  | **2011** | **-66.69** | **139.95** | **AdL** |  |
| **Not264** | **Not264** | **REVOLTA2** | **Notasterias armata** | **Christopher L. Mah** |  | **2011** | **-66.69** | **139.95** | **AdL** |  |
| **Not265** | **Not265** | **REVOLTA2** | **Notasterias armata** | **Christopher L. Mah** |  | **2011** | **-66.69** | **139.95** | **AdL** |  |
| **Not266** | **Not266** | **REVOLTA2** | **Notasterias armata** | **Christopher L. Mah** |  | **2011** | **-66.69** | **139.95** | **AdL** |  |
| **Psi028** | **Psi028** | **REVOLTA2** | **Psilaster charcoti** | **Christopher L. Mah** |  | **2011** | **-66.69** | **139.95** | **AdL** |  |
| **Bat151** | **Bat151** | **REVOLTA2** | **Bathybiaster loripes** | **Christopher L. Mah** |  | **2011** | **-66.65** | **139.95** | **AdL** |  |
| **Lys266** | **Lys266** | **REVOLTA2** | **Lysasterias cf. lactea** | **Christopher L. Mah** |  | **2011** | **-66.65** | **139.95** | **AdL** | **92** |
| **Lys267** | **Lys267** | **REVOLTA2** | **Lysasterias cf. lactea** | **Christopher L. Mah** |  | **2011** | **-66.65** | **139.95** | **AdL** | **92** |
| **Lys268** | **Lys268** | **REVOLTA2** | **Lysasterias cf. lactea** | **Christopher L. Mah** |  | **2011** | **-66.65** | **139.95** | **AdL** | **92** |
| **Lys269** | **Lys269** | **REVOLTA2** | **Lysasterias cf. lactea** | **Christopher L. Mah** |  | **2011** | **-66.65** | **139.95** | **AdL** | **92** |
| **Lys271** | **Lys271** | **REVOLTA2** | **Lysasterias cf. lactea** | **Christopher L. Mah** |  | **2011** | **-66.65** | **139.95** | **AdL** | **92** |
| **Lys272** | **Lys272** | **REVOLTA2** | **Lysasterias cf. lactea** | **Christopher L. Mah** |  | **2011** | **-66.65** | **139.95** | **AdL** | **92** |
| **Lys273** | **Lys273** | **REVOLTA2** | **Lysasterias cf. lactea** | **Christopher L. Mah** |  | **2011** | **-66.65** | **139.95** | **AdL** | **92** |
| **Lys274** | **Lys274** | **REVOLTA2** | **Lysasterias cf. lactea** | **Christopher L. Mah** |  | **2011** | **-66.65** | **139.95** | **AdL** | **92** |
| **Lys298** | **Lys298** | **REVOLTA2** | **Lysasterias cf. lactea** | **Christopher L. Mah** |  | **2011** | **-66.65** | **139.95** | **AdL** | **92** |
| **Not270** | **Not270** | **REVOLTA2** | **Notasterias cf. stolophora** | **Christopher L. Mah** |  | **2011** | **-66.65** | **139.95** | **AdL** | **92** |
| **Psi033** | **Psi033** | **REVOLTA1** | **Psilaster charcoti** | **Christopher L. Mah** |  | **2010** | **-66.68** | **139.96** | **AdL** |  |
| **Psi034** | **Psi034** | **REVOLTA1** | **Psilaster charcoti** | **Christopher L. Mah** |  | **2010** | **-66.68** | **139.96** | **AdL** |  |
| **Bat006** | **Bat006** | **REVOLTA2** | **Bathybiaster loripes** | **Christopher L. Mah** |  | **2011** | **-66.58** | **139.97** | **AdL** |  |
| **Lys281** | **Lys281** | **REVOLTA2** | **Lysasterias cf. lactea** | **Christopher L. Mah** |  | **2011** | **-66.62** | **139.97** | **AdL** | **116** |
| **Lys282** | **Lys282** | **REVOLTA2** | **Lysasterias cf. lactea** | **Christopher L. Mah** |  | **2011** | **-66.62** | **139.97** | **AdL** | **116** |
| **Lys283** | **Lys283** | **REVOLTA2** | **Lysasterias cf. lactea** | **Christopher L. Mah** |  | **2011** | **-66.62** | **139.97** | **AdL** | **116** |
| **Dip286** | **Dip286** | **REVOLTA2** | **Diplasterias brucei** | **Christopher L. Mah** |  | **2011** | **-66.68** | **139.97** | **AdL** | **34** |
| **Lys219** | **Lys219** | **REVOLTA2** | **Lysasterias sp** | **Christopher L. Mah** |  | **2011** | **-66.68** | **139.97** | **AdL** | **33** |
| **Lys220** | **Lys220** | **REVOLTA2** | **Lysasterias sp** | **Christopher L. Mah** |  | **2011** | **-66.68** | **139.97** | **AdL** | **33** |
| **Lys221** | **Lys221** | **REVOLTA2** | **Lysasterias sp** | **Christopher L. Mah** |  | **2011** | **-66.68** | **139.97** | **AdL** | **33** |
| **Dip266** | **Dip266** | **REVOLTA2** | **Diplasterias brucei** | **Christopher L. Mah** |  | **2011** | **-66.67** | **139.98** | **AdL** | **22** |
| **Dip283** | **Dip283** | **REVOLTA2** | **Diplasterias** | **Christopher L. Mah** |  | **2011** | **-66.67** | **139.98** | **AdL** | **22** |
| **Lys215** | **Lys215** | **REVOLTA2** | **Lysasterias sp** | **Christopher L. Mah** |  | **2011** | **-66.67** | **139.98** | **AdL** | **22** |
| **Lys216** | **Lys216** | **REVOLTA2** | **Lysasterias sp** | **Christopher L. Mah** |  | **2011** | **-66.67** | **139.98** | **AdL** | **22** |
| **Lys217** | **Lys217** | **REVOLTA2** | **Lysasterias sp** | **Christopher L. Mah** |  | **2011** | **-66.67** | **139.98** | **AdL** | **22** |
| **Lys218** | **Lys218** | **REVOLTA2** | **Lysasterias sp** | **Christopher L. Mah** |  | **2011** | **-66.67** | **139.98** | **AdL** | **22** |
| **Not272** | **Not272** | **REVOLTA2** | **Notasterias sp** | **Christopher L. Mah** |  | **2011** | **-66.67** | **139.98** | **AdL** |  |
| **Dip255** | **Dip255** | **REVOLTA2** | **Diplasterias brucei** | **Christopher L. Mah** |  | **2011** | **-66.67** | **139.98** | **AdL** | **22** |
| **Lys275** | **Lys275** | **REVOLTA2** | **Lysasterias cf. lactea** | **Christopher L. Mah** |  | **2011** | **-66.67** | **139.98** | **AdL** | **16** |
| **Lys276** | **Lys276** | **REVOLTA2** | **Lysasterias cf. lactea** | **Christopher L. Mah** |  | **2011** | **-66.67** | **139.98** | **AdL** | **16** |
| **Lys287** | **Lys287** | **REVOLTA2** | **Lysasterias sp** | **Christopher L. Mah** |  | **2011** | **-66.67** | **139.98** | **AdL** | **16** |
| **Lys288** | **Lys288** | **REVOLTA2** | **Lysasterias sp** | **Christopher L. Mah** |  | **2011** | **-66.67** | **139.98** | **AdL** | **16** |
| **Dip144** | **Dip144** | **REVOLTA1** | **Diplasterias brucei** | **Christopher L. Mah** |  | **2010** | **-66.66** | **139.99** | **AdL** | **30** |
| **Dip145** | **Dip145** | **REVOLTA1** | **Diplasterias brucei** | **Christopher L. Mah** |  | **2010** | **-66.66** | **139.99** | **AdL** | **30** |
| **Dip256** | **Dip256** | **REVOLTA2** | **Diplasterias brucei** | **Christopher L. Mah** |  | **2011** | **-66.67** | **139.99** | **AdL** | **36** |
| **Dip258** | **Dip258** | **REVOLTA2** | **Diplasterias brucei** | **Christopher L. Mah** |  | **2011** | **-66.67** | **139.99** | **AdL** | **36** |
| **Lys299** | **Lys299** | **REVOLTA2** | **Lysasterias sp** | **Christopher L. Mah** |  | **2011** | **-66.67** | **139.99** | **AdL** | **36** |
| **Dip135** | **Dip135** | **REVOLTA1** | **Diplasterias brandti** | **Christopher L. Mah** |  | **2010** | **-66.63** | **140.00** | **AdL** | **119** |
| **Not149** | **Not149** | **REVOLTA1** | **Notasterias armata** | **Christopher L. Mah** |  | **2010** | **-66.63** | **140.00** | **AdL** |  |
| **Not267** | **Not267** | **REVOLTA2** | **Notasterias armata** | **Christopher L. Mah** |  | **2011** | **-66.62** | **140.00** | **AdL** |  |
| **Not268** | **Not268** | **REVOLTA2** | **Notasterias armata** | **Christopher L. Mah** |  | **2011** | **-66.62** | **140.00** | **AdL** |  |
| **Psi146** | **Psi146** | **REVOLTA2** | **Psilaster charcoti** | **Christopher L. Mah** |  | **2011** | **-66.62** | **140.00** | **AdL** |  |
| **Dip146** | **Dip146** | **REVOLTA1** | **Diplasterias brucei** | **Christopher L. Mah** |  | **2010** | **-66.67** | **140.02** | **AdL** | **44** |
| **Dip147** | **Dip147** | **REVOLTA1** | **Diplasterias brucei** | **Christopher L. Mah** |  | **2010** | **-66.67** | **140.02** | **AdL** | **44** |
| **Dip148** | **Dip148** | **REVOLTA1** | **Diplasterias brucei** | **Christopher L. Mah** |  | **2010** | **-66.67** | **140.02** | **AdL** | **44** |
| **Lys290** | **Lys290** | **REVOLTA2** | **Lysasterias sp** | **Christopher L. Mah** |  | **2011** | **-66.63** | **140.02** | **AdL** | **88** |
| **Lys291** | **Lys291** | **REVOLTA2** | **Lysasterias sp** | **Christopher L. Mah** |  | **2011** | **-66.63** | **140.02** | **AdL** | **88** |
| **Lys292** | **Lys292** | **REVOLTA2** | **Lysasterias sp** | **Christopher L. Mah** |  | **2011** | **-66.63** | **140.02** | **AdL** | **88** |
| **Lys293** | **Lys293** | **REVOLTA2** | **Lysasterias sp** | **Christopher L. Mah** |  | **2011** | **-66.63** | **140.02** | **AdL** | **88** |
| **Lys294** | **Lys294** | **REVOLTA2** | **Lysasterias sp** | **Christopher L. Mah** |  | **2011** | **-66.63** | **140.02** | **AdL** | **88** |
| **Lys295** | **Lys295** | **REVOLTA2** | **Lysasterias sp** | **Christopher L. Mah** |  | **2011** | **-66.63** | **140.02** | **AdL** | **88** |
| **Dip257** | **Dip257** | **REVOLTA2** | **Diplasterias brucei** | **Christopher L. Mah** |  | **2011** | **-66.64** | **140.03** | **AdL** | **90** |
| **Lys289** | **Lys289** | **REVOLTA2** | **Lysasterias cf. lactea** | **Christopher L. Mah** |  | **2011** | **-66.64** | **140.03** | **AdL** | **90** |
| **Lys297** | **Lys297** | **REVOLTA2** | **Lysasterias cf. lactea** | **Christopher L. Mah** |  | **2011** | **-66.64** | **140.03** | **AdL** | **90** |
| **Dip276** | **Dip276** | **REVOLTA2** | **Diplasterias brucei** | **Christopher L. Mah** |  | **2011** | **-66.60** | **140.03** | **AdL** | **130** |
| **Dip277** | **Dip277** | **REVOLTA2** | **Diplasterias brucei** | **Christopher L. Mah** |  | **2011** | **-66.60** | **140.03** | **AdL** | **130** |
| **Dip278** | **Dip278** | **REVOLTA2** | **Diplasterias brucei** | **Christopher L. Mah** |  | **2011** | **-66.60** | **140.03** | **AdL** | **130** |
| **Dip279** | **Dip279** | **REVOLTA2** | **Diplasterias brucei** | **Christopher L. Mah** |  | **2011** | **-66.60** | **140.03** | **AdL** | **130** |
| **Not155** | **Not155** | **REVOLTA1** | **Notasterias armata** | **Christopher L. Mah** |  | **2010** | **-66.64** | **140.03** | **AdL** |  |
| **Not156** | **Not156** | **REVOLTA1** | **Notasterias armata** | **Christopher L. Mah** |  | **2010** | **-66.64** | **140.03** | **AdL** |  |
| **Lys296** | **Lys296** | **REVOLTA2** | **Lysasterias cf. lactea** | **Christopher L. Mah** |  | **2011** | **-66.66** | **140.03** | **AdL** | **100** |
| **Dip282** | **Dip282** | **REVOLTA2** | **Diplasterias brucei** | **Christopher L. Mah** |  | **2011** | **-66.64** | **140.05** | **AdL** | **40** |
| **Dip288** | **Dip288** | **REVOLTA2** | **Diplasterias brucei** | **Christopher L. Mah** |  | **2011** | **-66.64** | **140.05** | **AdL** | **40** |
| **Dip289** | **Dip289** | **REVOLTA2** | **Diplasterias brucei** | **Christopher L. Mah** |  | **2011** | **-66.64** | **140.05** | **AdL** | **40** |
| **Lys222** | **Lys222** | **REVOLTA2** | **Lysasterias sp** | **Christopher L. Mah** |  | **2011** | **-66.64** | **140.05** | **AdL** | **40** |
| **Lys223** | **Lys223** | **REVOLTA2** | **Lysasterias sp** | **Christopher L. Mah** |  | **2011** | **-66.64** | **140.05** | **AdL** | **40** |
| **Lys224** | **Lys224** | **REVOLTA2** | **Lysasterias sp** | **Christopher L. Mah** |  | **2011** | **-66.64** | **140.05** | **AdL** | **40** |
| **Lys225** | **Lys225** | **REVOLTA2** | **Lysasterias sp** | **Christopher L. Mah** |  | **2011** | **-66.64** | **140.05** | **AdL** | **40** |
| **Lys226** | **Lys226** | **REVOLTA2** | **Lysasterias sp** | **Christopher L. Mah** |  | **2011** | **-66.64** | **140.05** | **AdL** | **40** |
| **Not269** | **Not269** | **REVOLTA2** | **Notasterias armata** | **Christopher L. Mah** |  | **2011** | **-66.64** | **140.05** | **AdL** |  |
| **Lys033** | **Lys033** | **REVOLTA1** | **Lysasterias cf. lactea** | **Christopher L. Mah** |  | **2010** | **-66.64** | **140.08** | **AdL** | **45** |
| **Not157** | **Not157** | **REVOLTA1** | **Notasterias armata** | **Christopher L. Mah** |  | **2010** | **-66.64** | **140.08** | **AdL** |  |
| **Psi056** | **Psi056** | **CEAMARC** | **Psilaster charcoti** | **Christopher L. Mah** |  | **2007** | **-66.34** | **143.04** | **AdL** | **683.6** |
| **Psi229** | **Psi229** | **ACE_2017** | **Psilaster charcoti** | **Camille Moreau** |  | **2017** | **-66.16** | **162.18** | **Bal** | **357** |
| **Psi236** | **Psi236** | **ACE_2017** | **Psilaster charcoti** | **Camille Moreau** |  | **2017** | **-66.15** | **162.20** | **Bal** | **600** |
| **Psi237** | **Psi237** | **ACE_2017** | **Psilaster charcoti** | **Christopher L. Mah** |  | **2017** | **-66.15** | **162.20** | **Bal** | **600** |
| **Bat311** | **Bat311** |  |  | **Camille Moreau** |  |  |  |  | **AdL** |  |
| **Dip031** | **Dip031** | **XXVIII** | **Diplasterias brucei** | **Camille Moreau** |  |  |  |  | **Ross** |  |
| **Dip034** | **Dip034** | **XXVIII** | **Diplasterias brucei** | **Camille Moreau** |  |  |  |  | **Ross** |  |
| **Dip035** | **Dip035** | **XXVIII** | **Diplasterias brucei** | **Camille Moreau** |  |  |  |  | **Ross** |  |
| **Dip036** | **Dip036** | **XXVIII** | **Diplasterias brucei** | **Camille Moreau** |  |  |  |  | **Ross** |  |
| **Dip037** | **Dip037** | **XXVIII** | **Diplasterias brucei** | **Camille Moreau** |  |  |  |  | **Ross** |  |
| **Dip009** | **Dip009** | **Terri** | **Undet** | **Camille Moreau** |  | **2016** |  |  | **AntPen** |  |
| **Und031** | **Und031** | **Terri** | **Lysasterias sp** | **Camille Moreau** |  | **2016** |  |  | **AntPen** |  |
| **Und033** | **Und033** | **Terri** | **Lysasterias sp** | **Camille Moreau** |  | **2016** |  |  | **AntPen** |  |
| **Und034** | **Und034** | **Terri** | **Lysasterias sp** | **Camille Moreau** |  | **2016** |  |  | **AntPen** |  |
| **R1073_Dip2** |  | **REVOLTA** | **Diplasterias sp** | **Camille Moreau** |  | **2017** |  |  |  |  |
| **R1075_Dip2** |  | **REVOLTA** | **Diplasterias sp** | **Camille Moreau** |  | **2017** |  |  |  |  |
| **R1075_Dip3** |  | **REVOLTA** | **Diplasterias sp** | **Camille Moreau** |  | **2017** |  |  |  |  |
| **REVO_1075Diplasp1** |  | **REVOLTA** | **Diplasterias sp** | **Camille Moreau** |  | **2017** |  |  |  |  |

Appendix S1 Associated metadata for each COI sequence together with the Barcode of Life Data System ID. Locations are displayed in decimal degrees and as region. Abbreviations: Museo Nazionale dell’Antartide (MNA); Museum National d’Histoire Naturelle (MNHN); California Academy of Sciences (CAS); Ross Sea (Ross); Kerguelen Island (Ker) ; Elephant Island (Eleph) ; South Georgia (SG) ; Bouvet Island (Bouv) ; South Shetland (Sshet) ; South Orkneys (Sork) ; South Sandwich Islands (Ssand); ADélie Land (AdL); Weddell Sea (WS); Eastern Part of the Antarctic Peninsula (AntPenEast); Shag Rocks (Shag); Bellingshausen Sea (Belli); Balleny Islands (Bal); Magellanic (Mag); Amundsen Sea (Amund); Australia (Aus); New Zealand (NZ).
