# Supplementary material for: Is reproductive strategy a key factor in understanding the evolutionary history of Southern Ocean Asteroidea (Echinodermata)?
Source: Ecol Evol. 2019 Jul 16;9(15):8465–78. doi: 10.1002/ece3.5280 (PMC6686340; doi:10.1002/ece3.5280)

*Journal of Biogeography*

**APPENDICES**

**Reproductive strategy helps resolving the evolutionary history of Southern Ocean Asteroidea (Echinodermata)**

Camille Moreau, Bruno Danis, Quentin Jossart, Marc Eléaume, Chester Sands, Guillaume Achaz, Antonio Agüera, and Thomas Saucède

Appendix 2 Statistical Parsimony network indicating genetic relationships among sampling locations for each genera of interest. Major delineated clades are represented on maps attached to each haplotype network. Inter-specific distance between delineated entities using the K2P model are also presented for each genus.


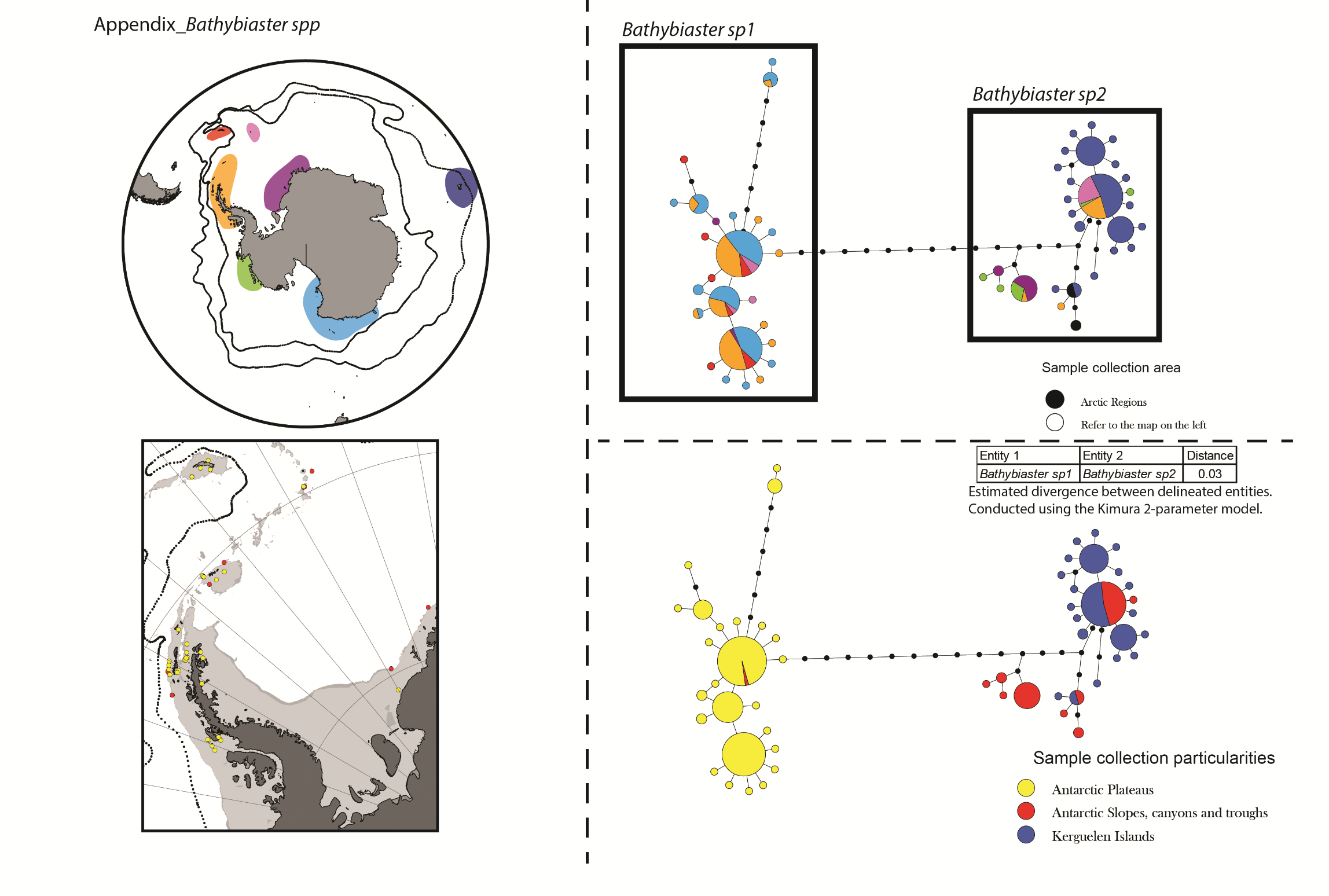


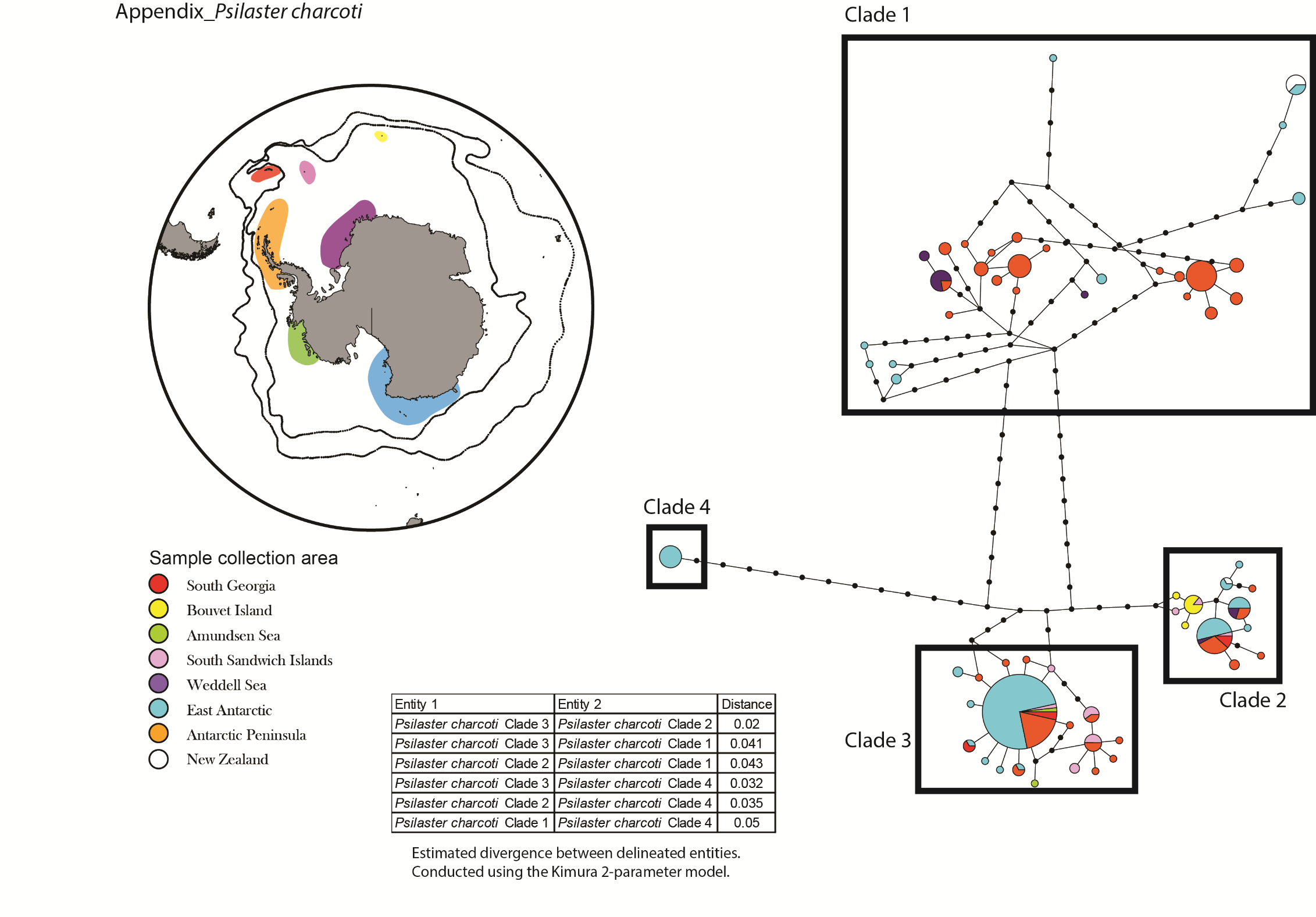


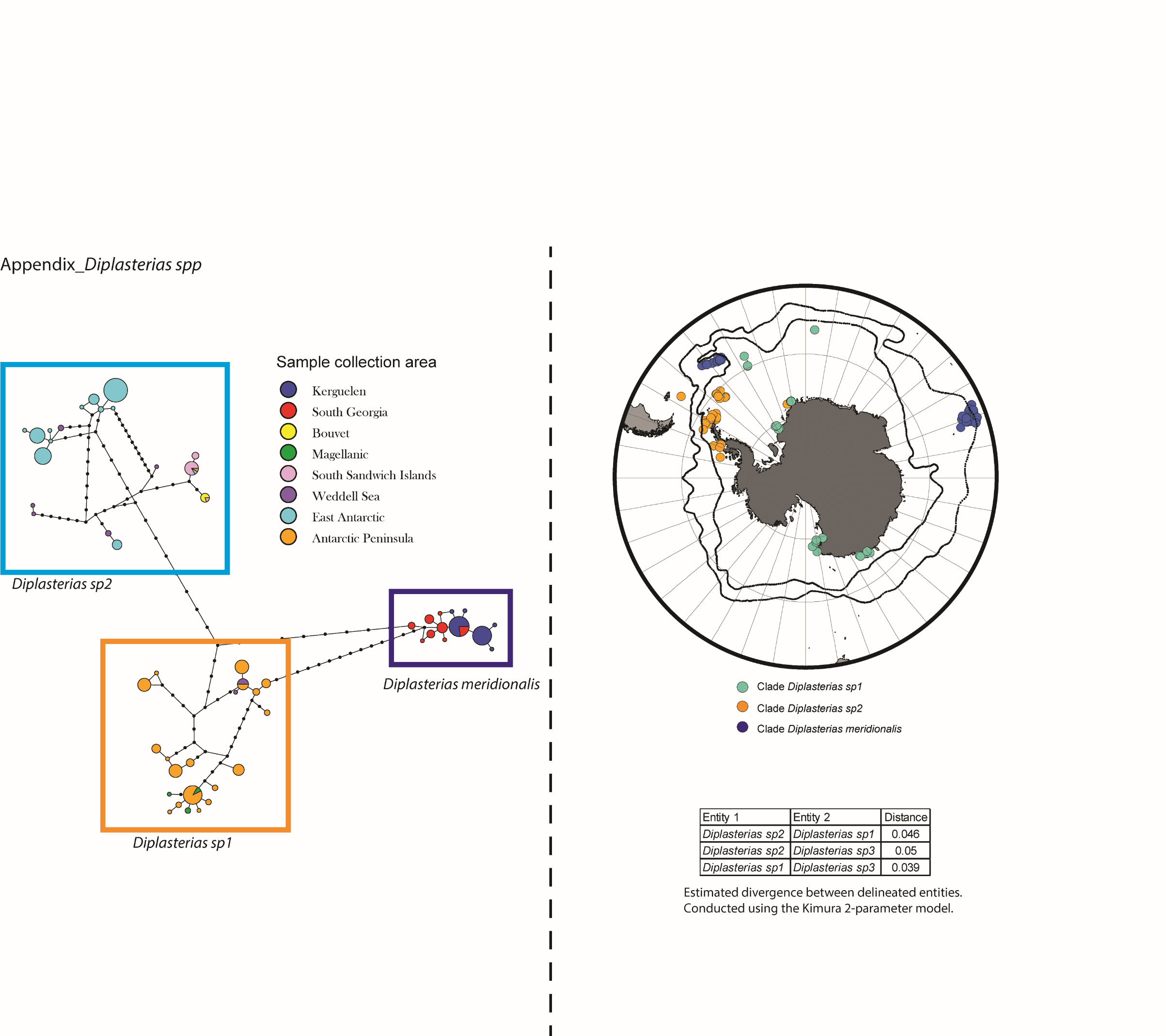


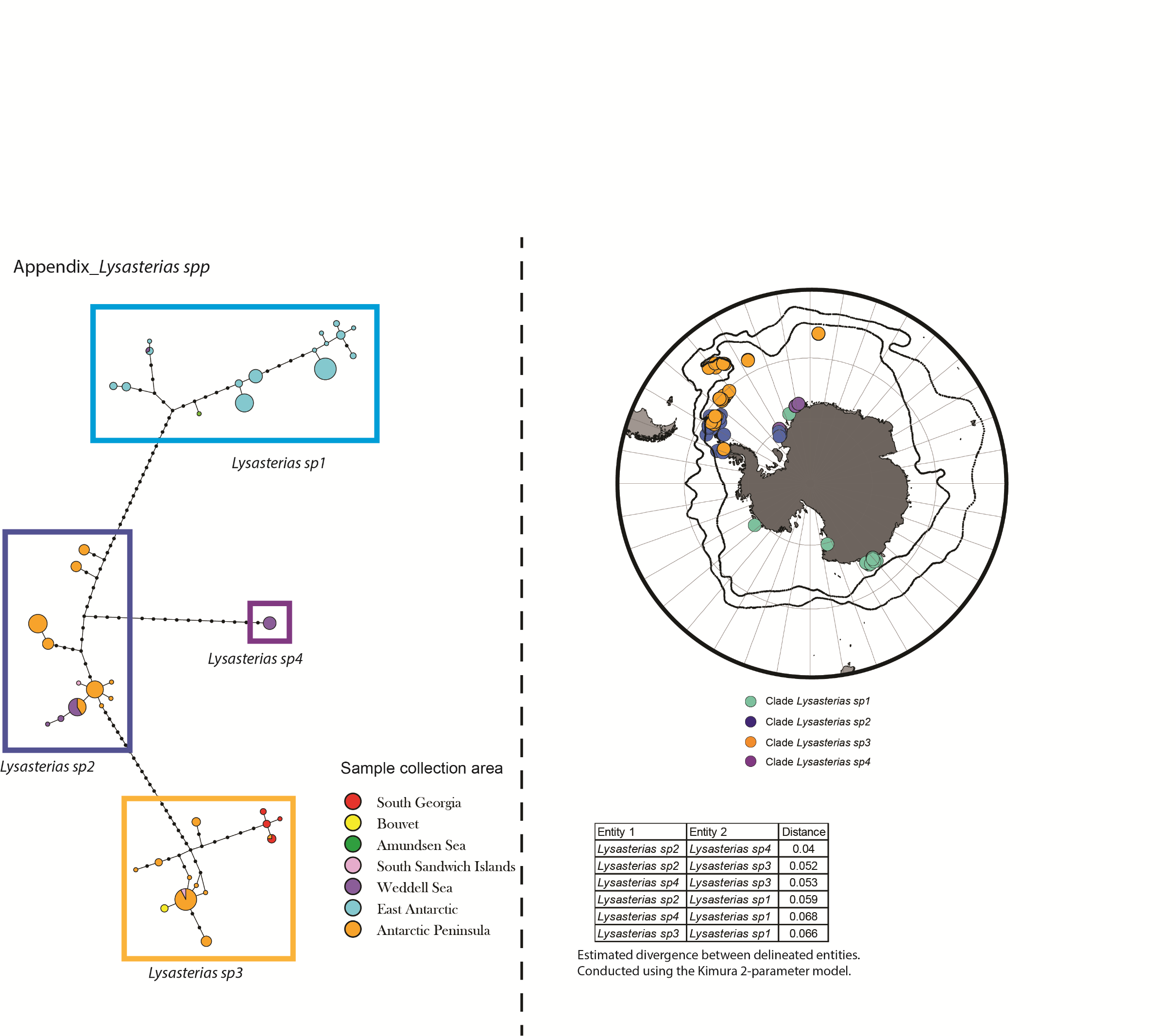


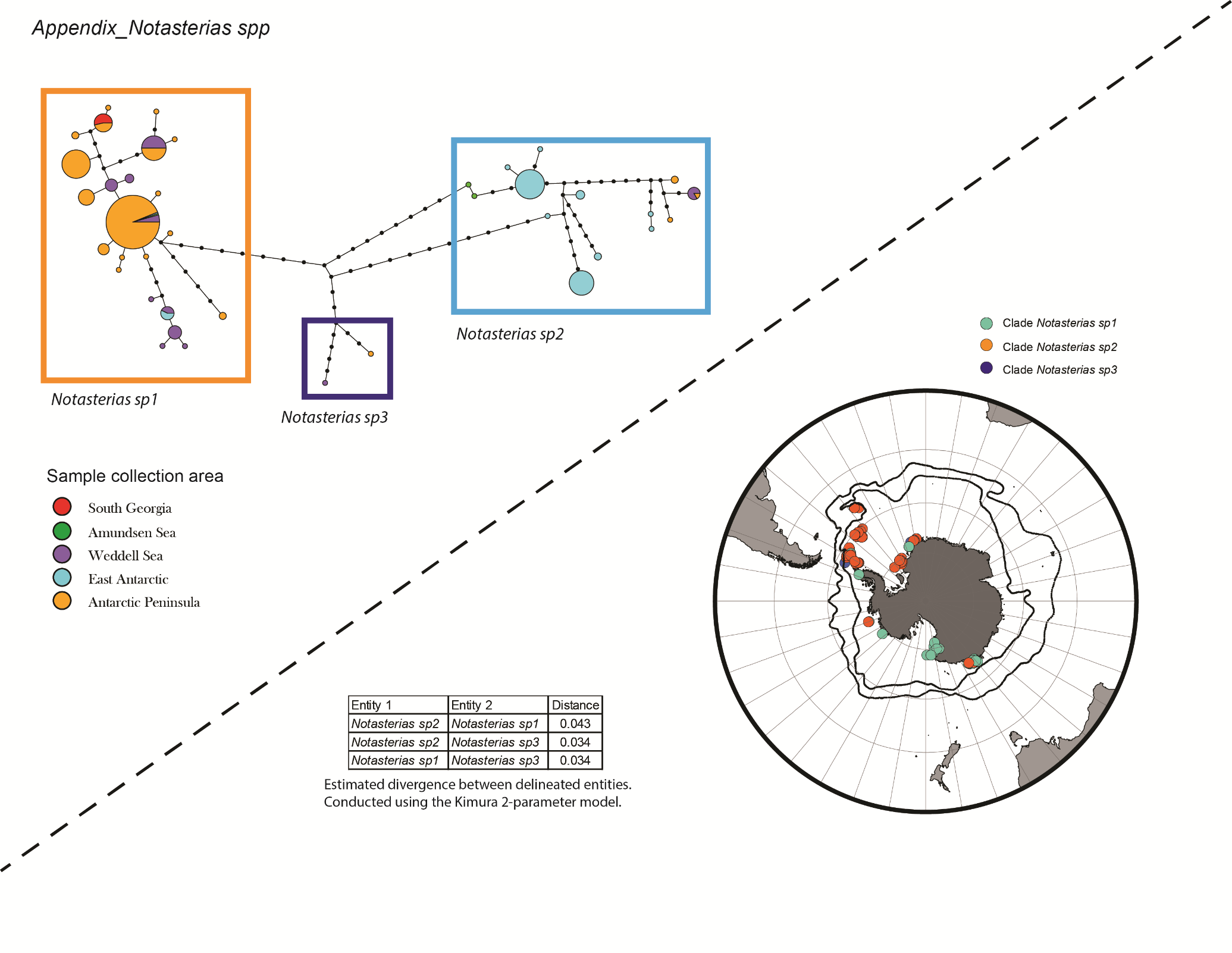

Supplement: Supplementary file 2 [file ECE3-9-8465-s002.docx]
