# Supplementary material for: Is reproductive strategy a key factor in understanding the evolutionary history of Southern Ocean Asteroidea (Echinodermata)?
Source: Ecol Evol. 2019 Jul 16;9(15):8465–78. doi: 10.1002/ece3.5280 (PMC6686340; doi:10.1002/ece3.5280)
Supplement: Supplementary file 3 [file ECE3-9-8465-s003.docx]

*Journal of Biogeography*

**APPENDICES**

**Reproductive strategy helps resolving the evolutionary history of Southern Ocean Asteroidea (Echinodermata)**

Camille Moreau, Bruno Danis, Quentin Jossart, Marc Eléaume, Chester Sands, Guillaume Achaz, Antonio Agüera, and Thomas Saucède

**Appendix 3 Nodes summary statistics for both phylogeny reconstructions**

| Astropectinidae | Node A | Node B | Node C | Node D |  |  |  |  |  |
| --- | --- | --- | --- | --- | --- | --- | --- | --- | --- |
| median (Myr) | 4.45 | 3.01 | 1.78 | 1.29 |  |  |  |  |  |
| 95% HPD interval (Myr) | [3.33, 5.70] | [2.23, 3.91] | [1.27, 2.38] | [0.85, 1.84] |  |  |  |  |  |
|  |  |  |  |  |  |  |  |  |  |
| Asteriidae | Pan-tropical Outgroup | Node I | Node II | Node III | Node IV | Node V | Node VI | Node VII | Node VIII |
| median (Myr) | 20.9 | 5.24 | 3.83 | 2.77 | 2.13 | 2.06 | 1.72 | 1.31 | 1.3 |
| 95% HPD interval (Myr) | [14.3, 29] | [4.05, 6.69] | [2.89, 4.95] | [2.01, 3.61] | [1.53, 2.83] | [1.49, 2.75] | [1.22, 2.31] | [0.90, 1.82] | [0.85, 1.80] |
